# Development of triaryl antimicrobials by scaffold hopping from an aminopropanol hit targeting bacterial RNA polymerase-NusG interactions

**DOI:** 10.1080/14756366.2025.2543923

**Published:** 2025-08-18

**Authors:** Tiankuang Liu, Cheuk Hei Kan, Yingbo Zheng, Tsz Fung Tsang, Yanpeng Liu, Man Wai Tsang, Hantian Fang, Long Yin Lam, Xiao Yang, Cong Ma

**Affiliations:** a State Key Laboratory of Chemical Biology and Drug Discovery, Department of Applied Biology and Chemical Technology, The Hong Kong Polytechnic University, Kowloon, Hong Kong SAR, China; b Marshall Research Centre for Medical Microbial Biotechnology, The Hong Kong Polytechnic University, Kowloon, Hong Kong SAR, China; c Department of Microbiology, The Chinese University of Hong Kong, Prince of Wales Hospital, Shatin, Hong Kong SAR, China

**Keywords:** Scaffold hopping, bacterial transcription, protein–protein interaction, RNA polymerase, NusG

## Abstract

Bacterial RNA polymerase (RNAP) requires the NusG factor to facilitate transcription, with the RNAP clamp-helix domain (CH) serving as the primary binding site for NusG and representing a promising target for antimicrobial intervention. In previous work, we unprecedentedly developed a pharmacophore model based on key clamp-helix residues (R270, R278, R281) at RNAP CH essential for NusG binding, which led to the identification of a hit compound exhibiting modest antimicrobial activity against *Streptococcus pneumoniae*. In this study, we designed a new class of triaryl inhibitors via scaffold hopping, substituting the linear structure of the hit compound with a benzene ring. Antimicrobial testing showed that several newly synthesised lead compounds achieved the minimum inhibitory concentration of 1 µg/mL against drug-resistant *S. pneumoniae*, superior to some marketed antibiotics. The following inhibitory and cell-based assays demonstrated the potential of these triaryl compounds as promising candidates for further development as novel antimicrobial agents.

## GRAPHICAL ABSTRACT

**Figure:**
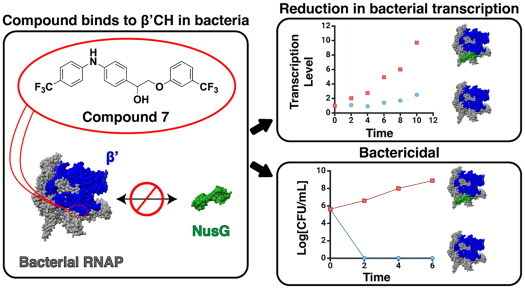

## Introduction

Precise transcriptional control is essential for bacterial survival and proper functioning. In bacteria, transcription can be divided into three stages: initiation, elongation, and termination, which collectively facilitate the synthesis of RNA molecules for protein production^1^. This process is regulated by transcription factors, including σ, NusA, NusB, NusE, and NusG^2^. Among them, NusG plays a key role in modulating various stages of transcription. It is a highly conserved protein found across all three domains of life, with functionally similar counterparts known as Spt5 in archaea and eukaryotes.

**Figure 1.**
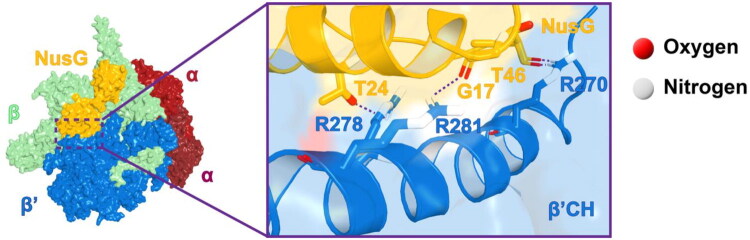
The crystal structure of *E. coli* RNA polymerase core enzyme (subunits ααββ′) bound to NusG (PDB 5TBZ)^10^. Left: the overall structure of the bacterial transcription complex (yellow: RNAP β′ subunit; blue: NusG). Right: the key hydrogen bond interactions between NusG and the β′CH region.

In bacteria, NusG functions as a monomeric transcription factor^3^ that directly interacts with the elongating RNA polymerase (RNAP) and downstream regulatory elements to enhance transcriptional efficiency^4^. Through its N-terminal domain, NusG binds to the clamp-helix (CH) region of the β′ subunit of RNAP (Figure 1)^5^, inducing conformational changes that stabilise the transcription bubble and facilitate the elongation of the RNA transcript^6^. Moreover, the C-terminal Kyrpides-Ouzounis-Woese (KOW) domain of NusG has been implicated in promoting Rho-dependent transcription termination by interacting with the Rho factor^7^^,^^8^.

Apart from its involvement in transcription elongation and termination, NusG also plays a critical role in coordinating the coupling of transcription and translation via the interaction with NusE, thereby ensuring the integrity of the mRNA molecule^9^. This multifaceted functionality of NusG underscores its importance as a key regulator of gene expression in bacterial systems.

**Figure 2.**
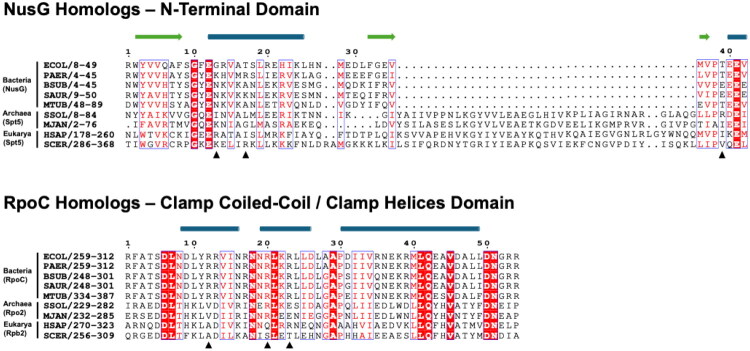
A multiple sequence alignment of selected clamp coiled-coil or clamp helices regions in the RpoC (RNAP β′ subunit) homologs and the N-Terminal Domain of NusG homologs from Bacteria, Archaea, and Eukarya. The alignment was generated using MAFFT^11^^,^^12^, and the amino acids responsible for hydrogen bond formation were marked with π symbols. Arginine residues in the β′CH domain are conserved within the Bacteria domain but not in the Archaea and Eukarya domains. The predicted secondary structure elements by JPred^13^ are depicted as green arrows for beta-strands and blue-grey rods for alpha-helices. The UniProt codes and species abbreviation can be found in the Supporting Information.

Given the central role of NusG in bacterial transcription, this protein emerges as an attractive target for developing novel antimicrobial agents. Its conservation across bacterial species, and its divergence from eukaryotic homologs (Supplementary Table S1) in terms of binding sites and amino acid sequences suggests that NusG is a promising candidate for selective inhibition without adversely affecting human cells (Figure 2). This divergence from eukaryotic counterparts highlights the potential for developing compounds that can selectively target the bacterial version of NusG, thereby offering a pathway to new antimicrobial therapies. Furthermore, there is no previously identified RNAP-NusG PPI inhibitor, highlighting the novelty of this research.

We have identified a hit compound, AW000783, which is capable of inhibiting the interaction between bacterial RNAP and NusG with weak antimicrobial activity (minimum inhibitory concentration, MIC; 128–256 µg/mL). In the initial studies, the synthesis of AW000783 analogues as RNAP-NusG inhibitors has been performed, and the antimicrobial activity was evaluated, leading to a representative compound with a reduced MIC at 1 µg/mL^14^. In this study, we designed and synthesised a new series of inhibitors featuring a triaryl ring structure through scaffold hopping from the original aminopropanol structure, in order to achieve the active conformation of a lead compound without bending its structure. As our primary objective was to disrupt the function of NusG and assess the potential of this novel class of inhibitors as antimicrobial therapeutics, we first screened the synthesised compounds for antimicrobial activity to ensure experimental efficiency, and then selected the representative compounds for subsequent microbiological, mechanistic, docking, and *in silico* ADMET studies. The results demonstrated that the new lead compound **7** can significantly eliminate antibiotic-resistant bacteria, and exhibit drug-like properties.

## Results and discussion

### Design of triaryl inhibitors by scaffold hopping

**Figure 3.**
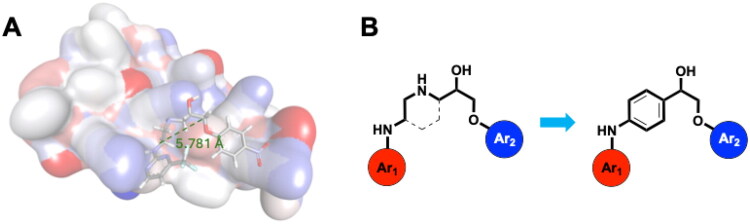
(A) Docking model of hit compound AW000783 with *E. coli* RNAP CH. The distance between 6 middle atoms is 5.781 Å. (B) Scaffold hoppling from the general structure of the linear hit compound to a new triaryl structure with a phenyl linker.

In the previous study, the linear analogues of AW000783 demonstrated the ability to bind to the RNAP β′CH, effectively inhibiting bacterial transcription and exhibiting notable antimicrobial activity. According to the docking model, AW000783 binds to the target protein in a bent conformation, despite its inherently linear structure (Figure 3(A)). The measured distance between the two carbons across six atoms in the central linker, which connects the two aromatic rings of AW000783, is 5.781 Å. Based on this observation, we hypothesise that replacing the linear linker with a ring structure could minimise molecular energy, thereby maintaining the active conformation. A benzene ring is proposed for this modification to validate our design due to its ease of substitution during synthesis and high occurrence in drug molecules (Figure 3(B)). Consequently, a new triaryl scaffold has been designed through scaffold hopping from the original bent linear compound.

### Chemistry

**Scheme 1.**
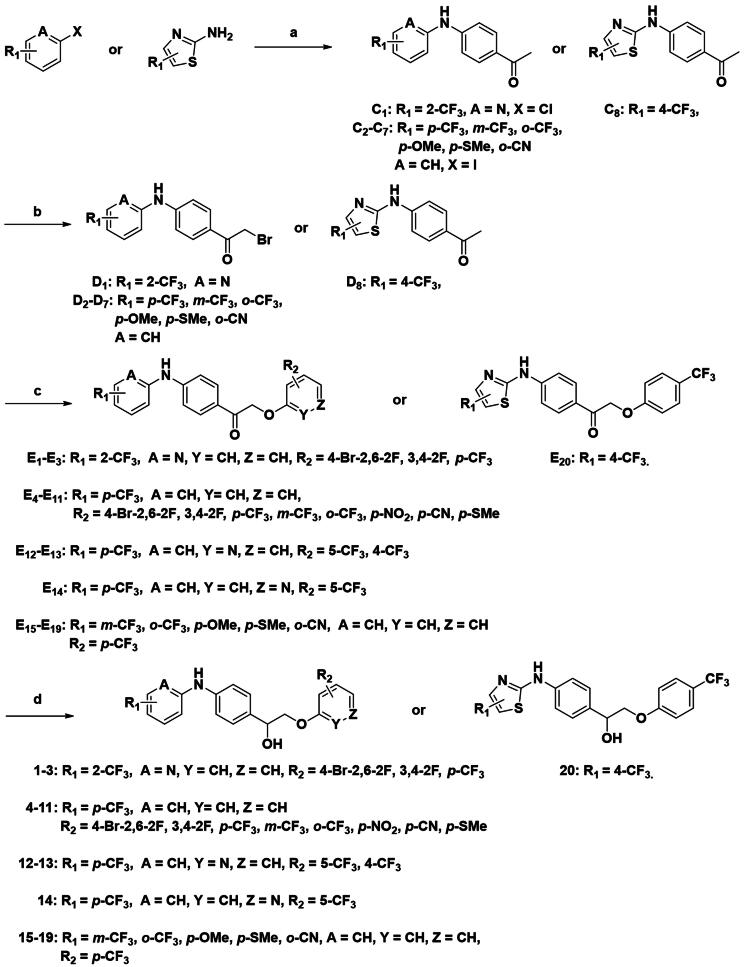
Synthetic route to compounds **1–20**. Reagents and conditions: (a) for **C1**–**C7**: aminoacetophenone, Pd(OAc)_2_, X-Phos, Cs_2_CO_3_, toluene, 120 °C, 8 h; for **C8**: bromoacetophenone, Pd_2_(dba)_3_, Cs_2_CO_3_, X-Phos, toluene, 80 °C, 24 h; (b) CuBr_2_, CHCl_3_: EA = 1: 1, 60 °C, overnight; (c) phenols, K_2_CO_3_, acetone, reflux, 6 h; (d) NaBH_4_, MeOH, rt, 8 h.

Scheme 1 depicts the general synthetic procedure for the preparation of target compounds **1–20** with focus on the modifications of the two terminal aromatic rings. The synthesis of substituted secondary amines **C1**–**C7** was performed by a palladium-catalyzed coupling reaction between substituted chloropyridine or iodobenzene and aminoacetophenone. Substituted amine thiazole reacted with bromoacetophenone under the same coupling reaction conditions to provide amine **C8**. Subsequently, bromination led to the formation of bromides **D1–D8**, followed by substitution reaction with substituted phenols to give ethers **E1–E20**. The ketone group was then reduced by sodium borohydride to furnish the desired target compounds **1–20**.

**Scheme 2.**
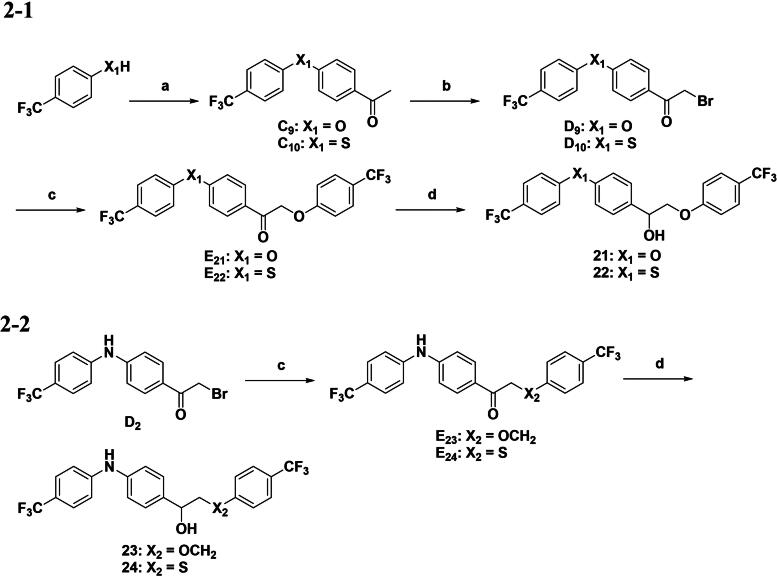
Synthetic route to compounds **21–24**. Reagents and conditions: (a) for **C9**–**C10**: fluoroacetophenone, Cs_2_CO_3_, NMP, 120 °C, overnight; (b) NBS, TsOH, MeCN, 90 °C, 8 h; (c) for **E21**–**E22**: *p*-CF_3_ phenol, K_2_CO_3_, acetone, reflux, 6 h; for **E23**: *p*-CF_3_ benzyl alcohol, K_2_CO_3_, acetone, reflux, 6 h; for **E24**: *p*-CF_3_ thiophenol, K_2_CO_3_, acetone, reflux, 6 h; (d) NaBH_4_, MeOH, rt, 8 h.

The synthetic routes for aromatic derivatives featuring alternative X_1_ and X_2_ linkers are illustrated in Schemes 2-1 and 2-2, respectively. For the X_1_ derivatives, a nucleophilic aromatic substitution reaction was employed, utilising either oxygen or sulphur nucleophiles for substitution at the X_1_ position. The subsequent bromination step was conducted using NBS. Following the formation of intermediates **C9**–**C10**, the subsequent steps adhered to the general synthetic route. In parallel, the synthetic route for derivatives with the alternative X_2_ moiety is depicted in Scheme 2-2. Intermediate **D2** reacted with the substituted phenol or benzyl alcohol reactants to yield intermediates **E23**–**E24**, which were then subjected to the final reduction step to afford the desired products **23** and **24**.

**Scheme 3.**
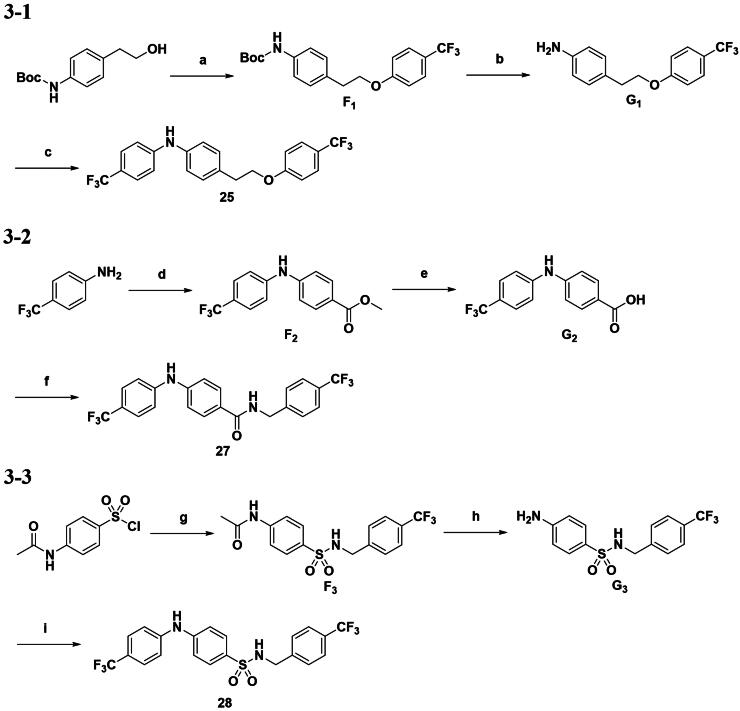
Synthetic route to compounds **25, 27–28**. Reagents and conditions: (a) *p*-CF_3_ phenol, PPh_3_, DEAD, THF, rt, overnight; (b) *p*-CF_3_ iodobenzene, TFA, DCM, reflux, 8 h; (c) iodobenzene, Pd(OAc)_2_, Cs_2_CO_3_, X-Phos, toluene, 120 °C, 8 h; (d) bromobenzoate, Pd(OAc)_2_, K_2_CO_3_, BINAP, toluene, 80 °C, 24 h; (e) NaOH, EtOH, reflux, 4 h; (f) *p*-CF_3_ phenylmethanamine, EDCI, 4-DMAP, DCM, rt, 24 h; (g) *p*-CF_3_ phenylmethanamine, Et_3_N, DCM, rt, 12 h; (h) HCl, EtOH, 70 °C, 4 h; (i) *p*-CF_3_ iodobenzene, Pd_2_(dba)_3_, Xantphos, NaO*t*Bu, toluene, 110 °C, 24 h.

Modifications on the central hydroxy group of the scaffold were conducted via three distinct synthetic routes, as illustrated in Scheme 3. These routes were designed for derivatives that either delete the hydroxy group or feature substitution by an amido or sulfonamino group. In Schemes 3-1 and 3-3, the synthesis of compounds **25** and **28** began with substitution and sulfonamidation reactions, respectively, followed by hydrolysis of the protecting groups, resulting in substituted aniline intermediates **G1** and **G3**. These intermediates served as substrates for the final step: a palladium-catalyzed coupling reaction, yielding the target compounds **25** and **28**. In parallel, a palladium-catalyzed coupling reaction provided amine **F2**, which, after hydrolysis of the methyl ester and subsequent amide synthesis, yielded compound **27**, as depicted in Scheme 3-2. Additionally, the corresponding β-oxy ketone analog was synthesised as intermediate **E6** in Scheme 1, which we have renamed as **26** to facilitate structure-activity relationship (SAR) analysis.

**Scheme 4.**
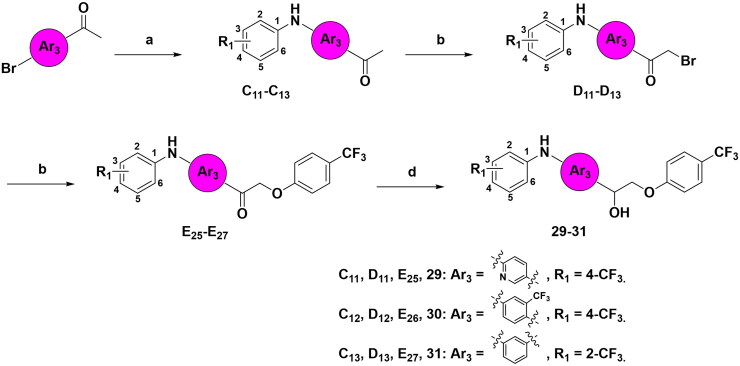
Synthetic route to compounds **29–31**. Reagents and conditions: (a) aromatic amines, Pd_2_(dba)_3_, Cs_2_CO_3_, X-Phos, toluene, 80 °C, 24 h; (b) CuBr_2_, CHCl_3_: EA = 1: 1, 60 °C, overnight; (c) *p*-CF_3_ phenols, K_2_CO_3_, acetone, reflux, 6 h; (d) NaBH_4_, MeOH, rt, 8 h.

The synthetic route for aromatic derivatives with an alternative central Ar_3_ group is depicted in Scheme 4. In this route, *p*-bromo arylethanone was utilised in a palladium-catalyzed coupling reaction to give intermediates **C11–C13**, the subsequent steps followed a pattern similar to that of the general synthetic route described in Scheme 1, leading to the formation of intermediates **D11**–**D13**, **E25**–**E27**, and the final compounds **29**–**31**.

**Scheme 5.**
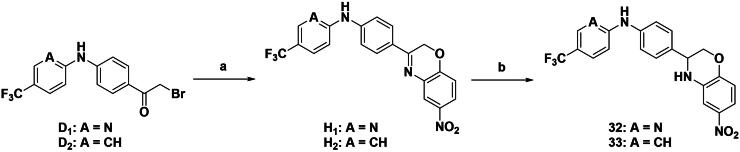
Synthetic route to compounds **32–33**. Reagents and conditions: (a) 2-amino-4-nitrophenol, tetrabutylammonium hydrogen sulphate, K_2_CO_3_, DCM, reflux, 6 h; (b) NaBH_4_, MeOH, rt, 8 h.

Scheme 5 illustrates the synthetic route for aromatic derivatives with a fused bicyclic aryl group. The reaction involved the utilisation of intermediates **D1** or **D2**, which underwent a cyclisation reaction with 2-amino-4-nitrophenol to generate Schiff base intermediates **H1** and **H2**. Following the reduction of the imine to an amine, the final products, **32** and **33**, were obtained.

### Antimicrobial activity against representative bacterial pathogens

A panel of bacteria was used for the antimicrobial activity evaluation on compounds **1**–**39**, including eight pathogens from the “WHO priority pathogens list, 2024”^15^. The panel consisted of four Gram-positive strains: *Enterococcus faecalis* ATCC 19433, *Staphylococcus aureus* ATCC 25923 and 29213, *Streptococcus pneumoniae* ATCC 49619; and five Gram-negative strains: *Klebsiella pneumoniae* ATCC 700603, *Acinetobacter baumannii* ATCC 19606, *Pseudomonas aeruginosa* ATCC 27853, *Enterobacter cloacae* ATCC 13047*, and Escherichia coli* ATCC 25922. Antibiotic susceptibility testing was performed using standard strains based on the Clinical & Laboratory Standards Institute (CLSI) guideline^16^.

In comparison to other hydrogen-bond acceptors such as nitro, cyano, fluoride, and methoxy groups, compounds featuring trifluoromethyl (CF_3_)-substituted aromatic rings exhibited significant activity against Gram-positive bacterial strains, as detailed in Table 1. Notably, compounds **7** and **9** demonstrated the most potent antimicrobial effects across all tested Gram-positive bacteria, with MIC values ranging from 2 to 8 µg/mL. Compounds **3**, **6**, and **15** showed comparable antimicrobial activity against *S. aureus* ATCC 25923 and *S. aureus* ATCC 29213, with slightly reduced efficacy against *E. faecalis* (**3** and **6**) and *S. pneumoniae* (**15**). SAR analysis revealed a preference for a benzene ring as the left aromatic group over heteroaryl groups such as pyridine (**1**–**3** vs. **4**–**6**). The CF_3_ substituent at *para*- and *meta*-positions was found to significantly enhance antimicrobial activity (**5**, **15** vs. **16**–**19**). Examination of the substituent position on the right aryl group indicated that CF_3_ at *meta*-position provided the greatest antimicrobial activity (**3** and **6**), with compound **7** outperforming compound **8**. Only the nitro group at *para*-position showed comparable activity (**9**), while other substituents (**10** and **11**) and heteroaryl groups (**12**–**14**) resulted in decreased activity. Furthermore, the use of a thiazole as the left aryl group (**20**) suggested that a six-membered aromatic ring is essential for antimicrobial efficacy. Interestingly, the triaryl derivatives of hit compound AW000783 exhibited activity exclusively against Gram-positive bacteria, likely due to the presence of an additional central aryl group, which may increase lipophilicity and reduce membrane permeability in Gram-negative bacteria.

**Table 1.** Antimicrobial activity of Compounds **1–20** (MIC µg/mL).

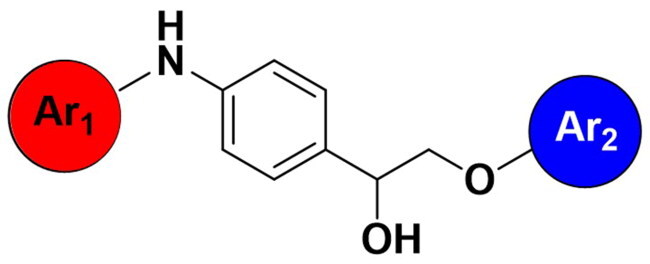											
No	Structure		MIC (µg/mL)								
	Ar_1_	Ar_2_	EFAE	SAUR^a^	SAUR^b^	SPNE	KPNE	ABAU	PAER	ECLO	ECOL
**1**	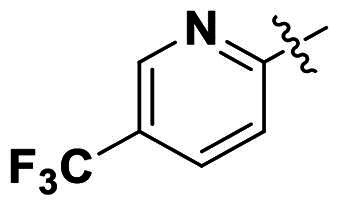	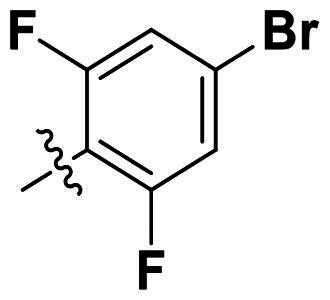	32	>256	8	32	>256	>256	>256	>256	>256
**2**	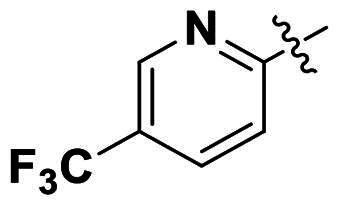	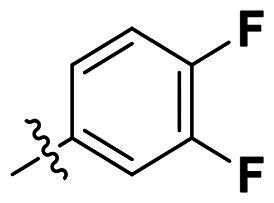	64	32	16	8	>256	>256	>256	>256	>256
**3**	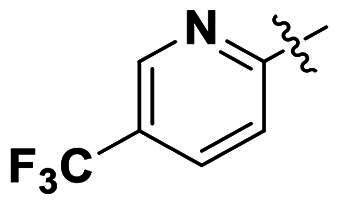	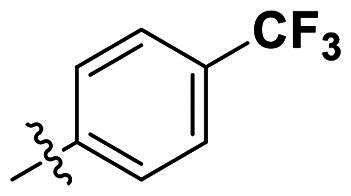	64	8	4	2	>256	>256	>256	>256	>256
**4**	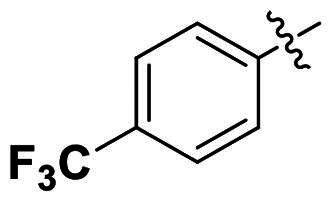	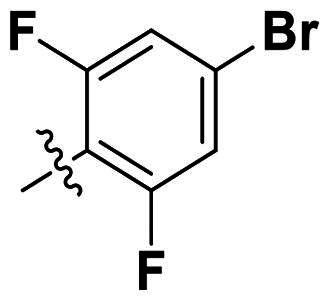	64	>256	64	16	>256	>256	>256	>256	>256
**5**	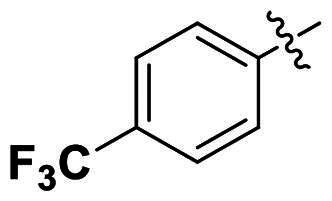	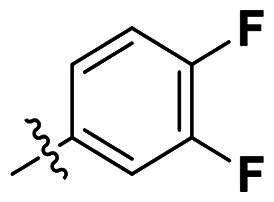	32	32	32	8	>256	>256	>256	>256	>256
**6**	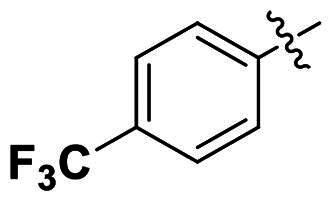	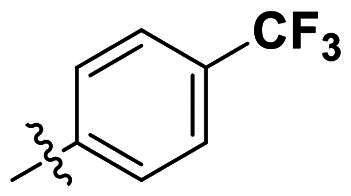	32	8	4	2	>256	>256	>256	>256	>256
**7**	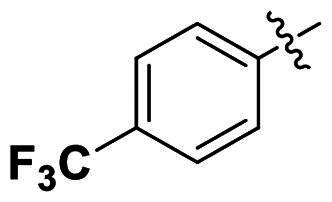	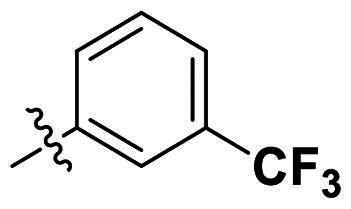	8	8	4	2	>256	>256	>256	>256	>256
**8**	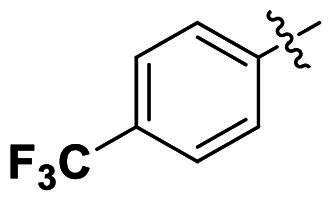	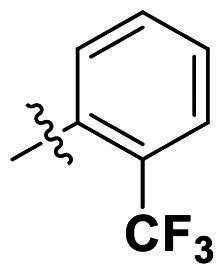	>256	64	32	8	>256	>256	>256	>256	>256
**9**	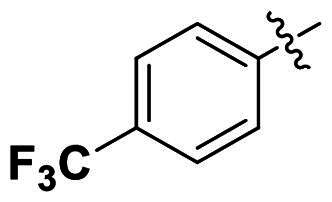	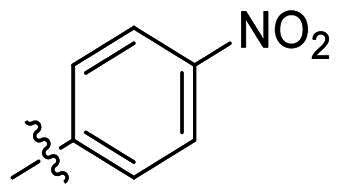	8	8	4	2	>256	>256	>256	>256	>256
**10**	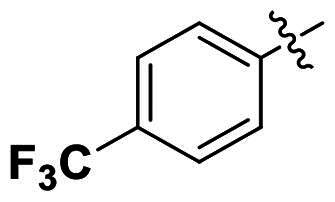	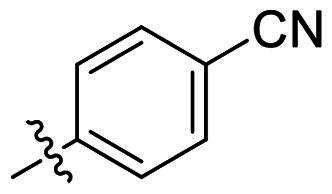	>256	>256	64	8	>256	>256	>256	>256	>256
**11**	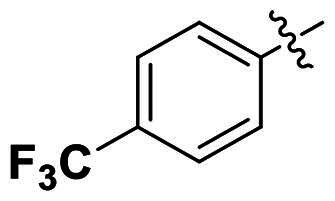	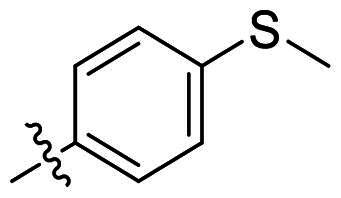	>256	>256	128	16	>256	>256	>256	>256	>256
**12**	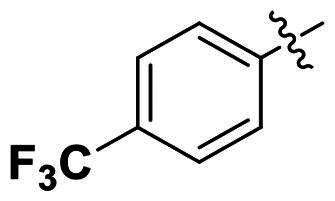	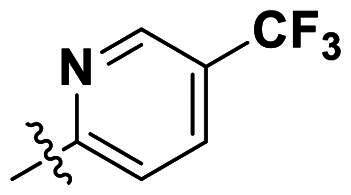	>256	16	8	16	>256	>256	>256	>256	>256
**13**	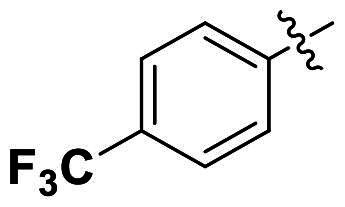	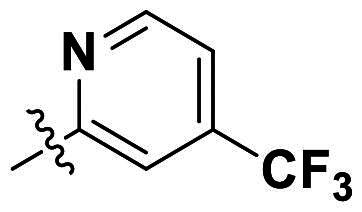	>256	>256	>256	128	>256	>256	>256	>256	>256
**14**	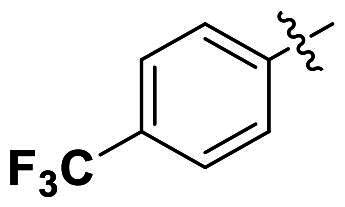	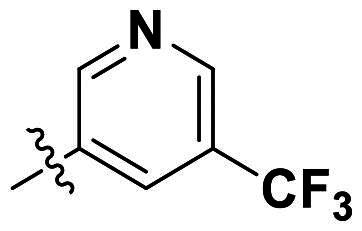	>256	32	16	8	>256	>256	>256	>256	>256
**15**	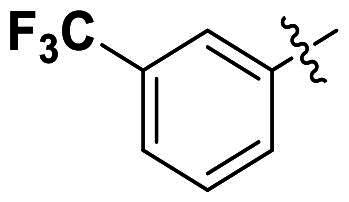	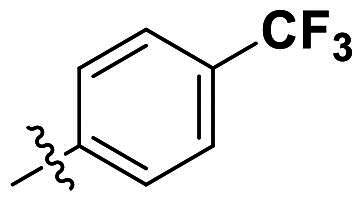	16	8	4	4	>256	>256	>256	>256	>256
**16**	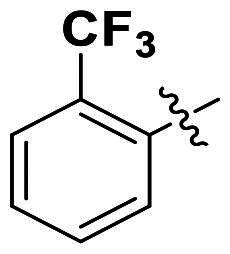	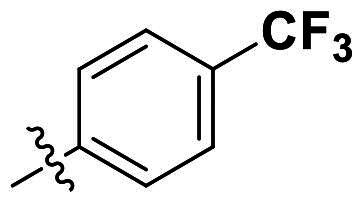	>256	>256	>256	4	>256	>256	>256	>256	>256
**17**	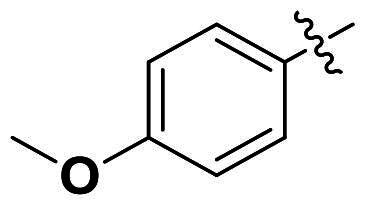	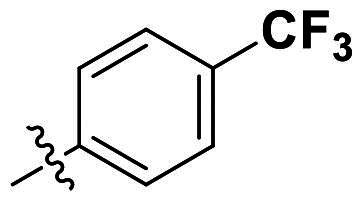	>256	>256	>256	128	>256	>256	>256	>256	>256
**18**	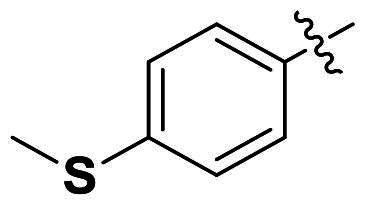	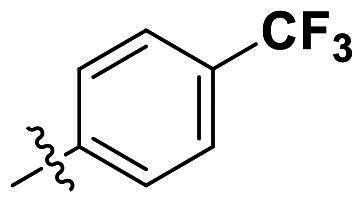	>256	>256	>256	32	>256	>256	>256	>256	>256
**19**	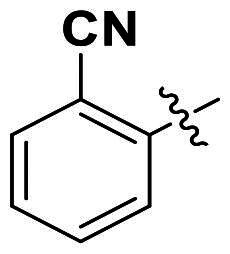	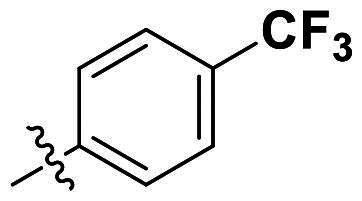	>256	>256	>256	16	>256	>256	>256	>256	>256
**20**	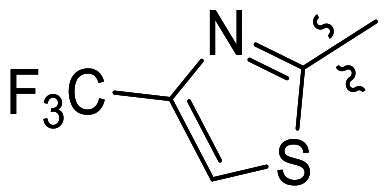	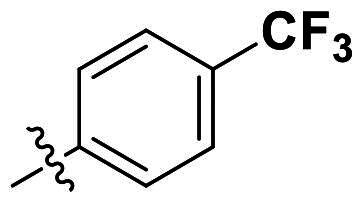	>256	>256	>256	>256	>256	>256	>256	>256	>256

EFAE: *E. faecalis* ATCC 19433, SAUR^a^: *S. aureus* ATCC 25923, SAUR^b^: *S. aureus* ATCC 29213, SPNE: *S. pneumoniae* ATCC 49619, KPNE: *K. pneumoniae* ATCC 700603, ABAU: *A. baumannii* ATCC 19606, PAER: *P. aeruginosa* ATCC 27853, ECLO: *E. cloacae* ATCC 13047, ECOL: *E. coli* ATCC 25922.

We selected compound **6** as the model compound to explore the effects of varying heteroatom linkers between aryl groups, as detailed in Table 2. Following a strategy similar to our previous work^14^, we employed nitrogen, oxygen, sulphur, and oxymethylene as isosteres to replace the heteroatoms in compound **6**. Surprisingly, most derivatives exhibited reduced antimicrobial activity. This suggests that the triaryl derivatives possess a relatively rigid structure, which may not accommodate isosteric modifications effectively. Notably, compound **23**, which features an additional carbon in the right linker, demonstrated the highest activity, with an MIC at 4 µg/ml against *S. pneumoniae.*

**Table 2.** Antimicrobial activity of Compounds **21–24** (MIC µg/mL).

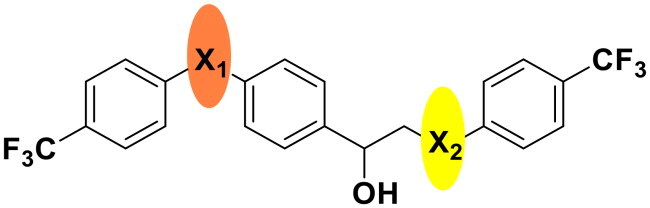											
No	Structure		MIC (µg/mL)								
	X_1_	X_2_	EFAE	SAUR^a^	SAUR^b^	SPNE	KPNE	ABAU	PAER	ECLO	ECOL
**21**	O	O	>256	>256	>256	16	>256	>256	>256	>256	>256
**22**	S	O	>256	>256	>256	32	>256	>256	>256	>256	>256
**23**	NH	OCH_2_	>256	128	16	4	>256	>256	>256	>256	>256
**24**	NH	S	32	128	64	16	>256	>256	>256	>256	>256

EFAE: *E. faecalis* ATCC 19433, SAUR^a^: *S. aureus* ATCC 25923, SAUR^b^: *S. aureus* ATCC 29213, SPNE: *S. pneumoniae* ATCC 49619, KPNE: *K. pneumoniae* ATCC 700603, ABAU: *A. baumannii* ATCC 19606, PAER: *P. aeruginosa* ATCC 27853, ECLO: *E. cloacae* ATCC 13047, ECOL: *E. coli* ATCC 25922.

Modifications were made to the hydroxy group of the right linker in compound **6** to investigate its role in the binding of triaryl derivatives with β′CH, as shown in Table 3. In the docking model of the hit compound, the hydroxy group was observed to fit into the pharmacophore model, although its precise binding function remains unclear. Compounds **25** and **26** exhibited almost no activity, suggesting that the hydroxy group is essential in the right linker for maintaining antimicrobial efficacy. In contrast, when the linking moiety Y was modified to amido and sulfonamino groups, which can function as both hydrogen bond acceptors and donors, compounds **27** and **28** displayed antimicrobial activity. This result supports the docking model, suggesting that the hydroxy group primarily acts as a hydrogen-bond donor, interacting with key residues on the target protein. However, compounds **27** and **28** showed reduced activity compared to **6**, likely due to the altered position of the amino group in these compounds, which may lead to suboptimal binding.

**Table 3.** Antimicrobial activity of Compounds **25–28** (MIC µg/mL).

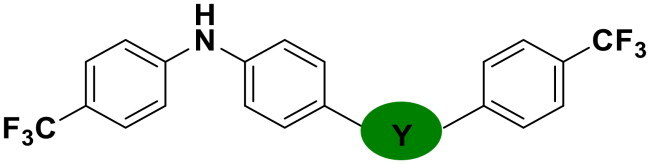										
No	Structure	MIC (µg/mL)								
	Y	EFAE	SAUR^a^	SAUR^b^	SPNE	KPNE	ABAU	PAER	ECLO	ECOL
**25**	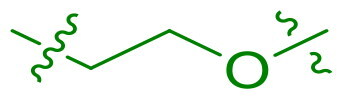	>256	>256	>256	128	>256	>256	>256	>256	>256
**26**	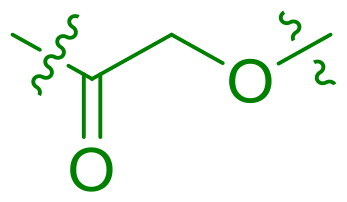	>256	>256	>256	>256	>256	>256	>256	>256	>256
**27**	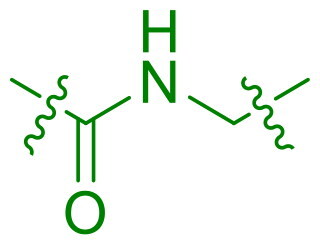	>256	>256	32	32	>256	>256	>256	>256	>256
**28**	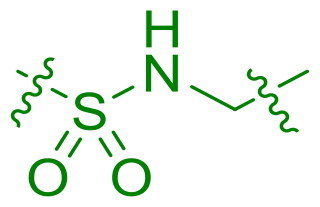	>256	16	64	16	>256	>256	>256	>256	>256

EFAE: *E. faecalis* ATCC 19433, SAUR^a^: *S. aureus* ATCC 25923, SAUR^b^: *S. aureus* ATCC 29213, SPNE: *S. pneumoniae* ATCC 49619, KPNE: *K. pneumoniae* ATCC 700603, ABAU: *A. baumannii* ATCC 19606, PAER: *P. aeruginosa* ATCC 27853, ECLO: *E. cloacae* ATCC 13047, ECOL: *E. coli* ATCC 25922.

Modifications were made on the central aryl group, including replacement with a heteroaromatic ring, addition of substitution groups, and alteration of the bonding position, as shown in Table 4. Among these compounds, compound **29**, which features a central pyridine, exhibited slightly decreased activity compared to compound **6**. However, a significant decline in activity was observed when Ar_3_ was substituted with a CF_3_ group (**30**) or when the bonding position was changed from *para* to *meta* (**31**). These results suggest that the addition of substituents or changes in bonding position on Ar_3_ may alter the compound’s conformation, leading to a complete loss of activity.

**Table 4.** Antimicrobial activity of the Compounds **29–31** (MIC µg/mL).

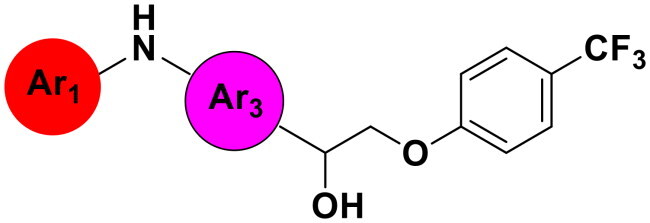											
No	Structure		MIC (µg/mL)								
	Ar_1_	Ar_3_	EFAE	SAUR^a^	SAUR^b^	SPNE	KPNE	ABAU	PAER	ECLO	ECOL
**29**	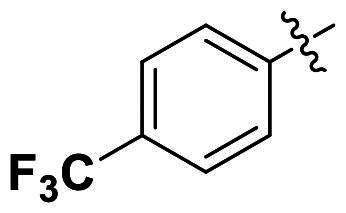	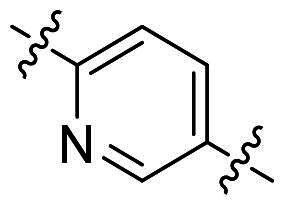	32	32	32	8	>256	>256	>256	>256	>256
**30**	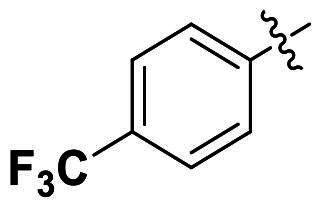	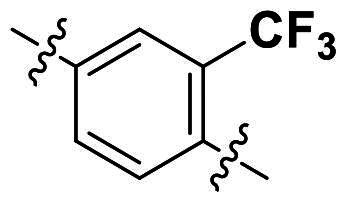	64	>256	>256	32	>256	>256	>256	>256	>256
**31**	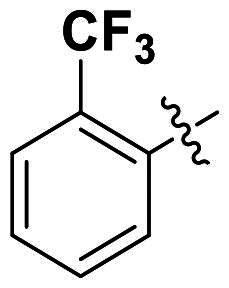	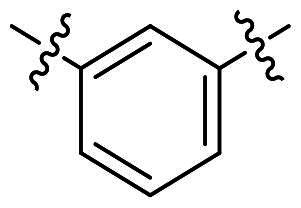	>256	>256	>256	8	>256	>256	>256	>256	>256

EFAE: *E. faecalis* ATCC 19433, SAUR^a^: *S. aureus* ATCC 25923, SAUR^b^: *S. aureus* ATCC 29213, SPNE: *S. pneumoniae* ATCC 49619, KPNE: *K. pneumoniae* ATCC 700603, ABAU: *A. baumannii* ATCC 19606, PAER: *P. aeruginosa* ATCC 27853, ECLO: *E. cloacae* ATCC 13047, ECOL: *E. coli* ATCC 25922.

We also synthesised and evaluated derivatives in which the right linker was fused into a bicyclic structure with the right aryl group. This further rigidified structure demonstrated selective antimicrobial activity against *S. pneumoniae* with an MIC at 4 µg/mL (**33**, Table 5).

**Table 5.** Antimicrobial activity of the Compounds **32–33** (MIC µg/mL).

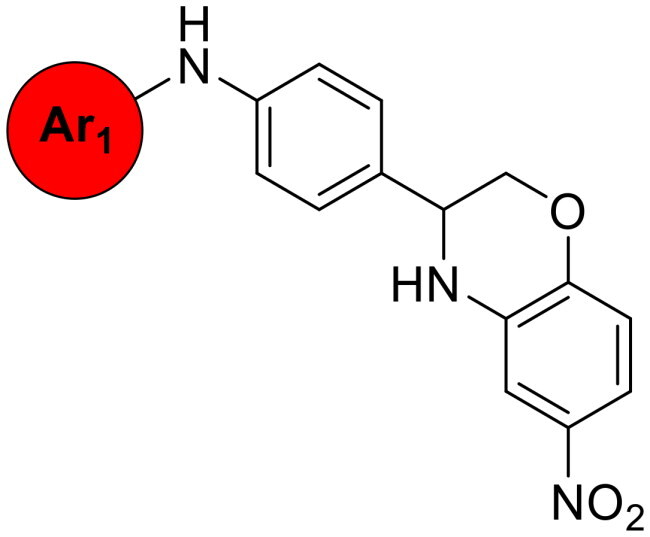										
No	Structure	MIC (µg/mL)								
	Ar_1_	EFAE	SAUR^a^	SAUR^b^	SPNE	KPNE	ABAU	PAER	ECLO	ECOL
**32**	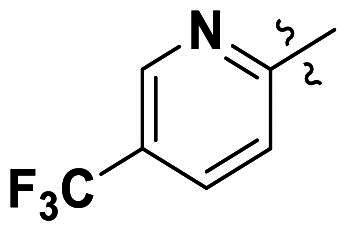	>256	>256	>256	32	>256	>256	>256	>256	>256
**33**	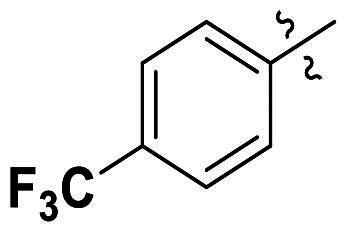	>256	>256	>256	4	>256	>256	>256	>256	>256

EFAE: *E. faecalis* ATCC 19433, SAUR^a^: *S. aureus* ATCC 25923, SAUR^b^: *S. aureus* ATCC 29213, SPNE: *S. pneumoniae* ATCC 49619, KPNE: *K. pneumoniae* ATCC 700603, ABAU: *A. baumannii* ATCC 19606, PAER: *P. aeruginosa* ATCC 27853, ECLO: *E. cloacae* ATCC 13047, ECOL: *E. coli* ATCC 25922.

**Figure 4.**
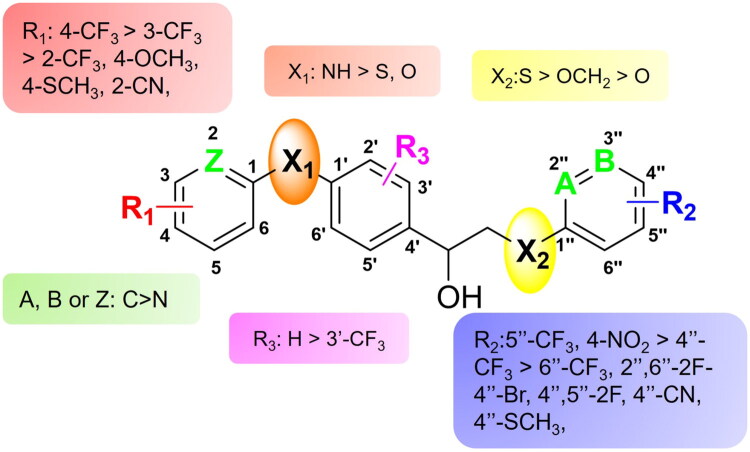
SAR of the synthesised inhibitor compounds as antimicrobials.

The SAR is summarised in Figure 4: Both the left and right aryl groups can be heteroaryl rings, such as pyridine (A and/or B and/or C = N), although a benzene ring is generally more favourable. Substituents R_1_ on the left ring show a preference for electron-withdrawing groups, such as CF_3_, particularly at *para*-position. The left linker X_1_ demonstrates improved antimicrobial activity when NH replaces O or S. Conversely, substituents R_2_ on the right ring favour electron-withdrawing groups at *meta*-position. For the right linker X_2_, O is preferred over S and OCH_2_. For the central ring, it is optimal to have no substitution and *para*-bonding.

Following the preliminary assessment of the antimicrobial potential of our compounds, we proceeded to an extensive screening process against a panel of clinically significant pathogens. Remarkably, the compound series exhibited notable antimicrobial activity against *S. pneumoniae*, prompting us to expand our evaluation to include *Streptococcus pyogenes* (Group A *Streptococcus*), which is known to cause strep throat, localised skin infections, and necrotising fasciitis^17^, and *Streptococcus agalactiae* (Group B *Streptococcus*), the causative agent of neonatal infections^18^. Furthermore, we assessed the antimicrobial activity of our compounds on clinically relevant Gram-positive pathogens, including *Staphylococcus epidermidis* and *Staphylococcus saprophyticus*. To further validate the antimicrobial activity against *S. pneumoniae*, we included a selection of clinical isolates of this bacterium, identified as CUHK-X01-04 (Supplementary Table S2). The identities of these isolates have been confirmed using Bruker MALDI Biotyper^®^ and are listed in the Supporting Information.

The findings in Table 6 show the favourable antimicrobial efficacy of our compounds against clinically demanding pathogens, as evidenced by MICs ranging from 2 µg/mL to 16 µg/mL. Particularly noteworthy is the potent antimicrobial activity of compound **7** against *S. agalactiae*, with an MIC comparable to that of vancomycin (2 µg/mL). In addition, all other compounds exhibited antimicrobial effects against the specified pathogens, with MIC values on par with those observed for other Gram-positive bacteria shown in Tables 1–5.

**Table 6.** Antimicrobial activity (MIC µg/mL) evaluation of NusG derivatives against representative pathogenic Gram-positive bacteria.

Compound	SEPI	SSAP	SPYO	SAGA
**3**	8	8	4	4
**6**	8	8	4	4
**7**	8	8	4	2
**9**	8	8	4	8
**15**	16	16	8	8
**Van**	2	2	0.5	1
**Cip**	0.5	1	1	4
**Oxa**	0.25	2	0.125	1
**Gen**	0.125	≤0.0625	32	64

SEPI: *S. epidermidis* ATCC 12228, SSAP: *S. saprophyticus* ATCC 15305, SPYO: *S. pyogenes* (group A Streptococcus) ATCC 19615, SAGA: *S. agalactiae* (group B Streptococcus) ATCC 12386. Van: Vancomycin, Cip: Ciprofloxacin, Oxa: Oxacillin, Gen: Gentamicin.

Table 7 presents additional observations regarding the inhibitory capabilities of our compounds against *S. pneumoniae*, with MICs as low as 1 µg/mL. Notably, compounds **6** and **9** displayed a broad spectrum of antimicrobial activity against all strains tested. In summary, our findings indicate that the compound series possesses promising antimicrobial potential against Gram-positive bacteria, particularly *S. pneumoniae*.

**Table 7.** Antimicrobial activity (MIC µg/mL) evaluation of NusG derivatives against an array of *S. pneumoniae* strains, including clinical isolates (CUHK-01–04).

Compound	SPNE^a^	SPNE^b^	SPNE^c^	CUHK-X01	CUHK-X02	CUHK-X03	CUHK-X04
**3**	2	4	2	1	2	2	8
**6**	2	2	1	1	2	2	4
**7**	2	4	2	1	2	2	4
**9**	2	2	2	1	2	2	4
**15**	4	4	4	2	4	4	8
**Van**	1	0.5	1	0.5	1	1	0.5
**Cip**	2	1	2	2	2	4	4
**Oxa**	2	16	8	≤0.0625	4	0.125	64
**Gen**	32	8	16	32	64	64	32

Clinical isolates (CUHK-01–04): See the Supporting Information. Van: Vancomycin, Cip: Ciprofloxacin, Oxa: Oxacillin, Gen: Gentamicin.

SPNE^a^: *S. pneumoniae* ATCC 49619, SPNE^b^: *S. pneumoniae* strain TCH8431 (HM-145), SPNE^c^: *S. pneumoniae* strain NP112 (NR-19213).

### Time-kill kinetics

**Figure 5.**
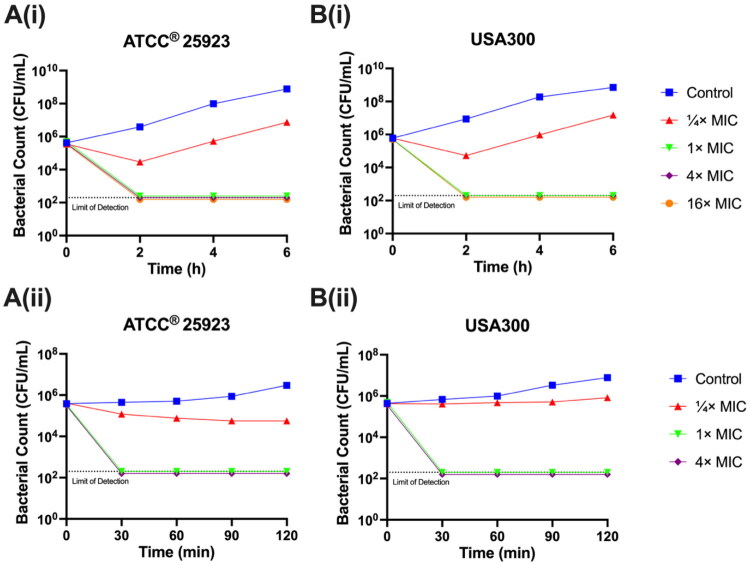
The effect of compound 7 on the time-kill kinetics of (A) *S. aureus* ATCC^®^ 25923 and (B) CA-MRSA strain USA300 over (i) 6 h and (ii) 2 h in cation-adjusted Muller-Hinton broth (CA-MHB). Experiments were performed in triplicates. 25923: *S. aureus* ATCC^®^ 25923; USA300: CA-MRSA strain USA300.

Under the guidelines set by CLSI^19^, we conducted a time-kill kinetic assay to evaluate the *in vitro* activity of the antimicrobial agent against a specific bacterial strain within a defined time frame. In this study, we aimed to assess the time-kill kinetics of compound **7** against two subtypes of *S. aureus*, ATCC 25923 and community-acquired methicillin resistant *S. aureus* (CA-MRSA) strain USA300, in liquid culture (Figure 5). After a 2-h treatment with compound **7**, both subtypes of *S. aureus* exhibited an inability to grow at or above 1× MIC.

Following the potent antimicrobial effects demonstrated by compound **7** within 2 h, a subsequent time-kill kinetic experiment was conducted with a shorter time interval. In this follow-up study, cells were collected every 30 min over a 2-h time course. Notably, as depicted in Figure 5(B), a 30-min treatment with compound **7** completely eradicated the bacterial population below the theoretical detection level, 200 colony-forming unit (CFU)/mL, at or above 1× MIC. Moreover, when the treatment period was extended to 120 min at ¼× MIC concentration, the CFU/mL count remained sustained, suggesting that the growth of bacteria was inhibited by compound **7** even at sub-MIC concentrations. These findings suggest that compound **7** could inhibit bacterial growth at sub-MIC concentrations and eradicate bacteria below the detectable threshold at 1× MIC or above concentrations.

### Bactericidal property

The minimum bactericidal concentration (MBC) of compound **7** was determined in accordance with CLSI guidelines^19^. Briefly, 2 × 10 µL droplets of bacterial cultures, prepared at concentrations ranging from 1× MIC to 16× MIC, were plated on agar and incubated overnight at 37 °C. Colonies were subsequently counted, and the MBC was defined as the lowest concentration at which the colony count dropped below the rejection threshold.

As summarised in Table 8, the majority of the tested compounds exhibited bactericidal activity against *S. aureus* strains, including ATCC^®^ 25923 and CA-MRSA USA300. The MBC values were within two-fold of the MIC values, indicating strong bactericidal activities. In comparison, control antibiotics showed variable activity: vancomycin demonstrated bactericidal effects, whereas linezolid was bacteriostatic, consistent with previous reports^20^^,^^21^. These findings highlight the potential of compound **7** as a bactericidal agent, particularly against drug-resistant *S. aureus* strains.

**Table 8.** MIC and MBC (µg/mL) of compound **7** against *S. aureus* strains.

Compound	*S. aureus* ATCC^®^ 25923		CA-MRSA USA300	
	MIC (µg/mL)	MBC (µg/mL)	MIC (µg/mL)	MBC (µg/mL)
**7**	8	16	16	16
**Vancomycin**	1	1	1	2
**Linezolid**	2	>32	4	>64

### NusG-β′CH PPI inhibition

**Figure 6.**
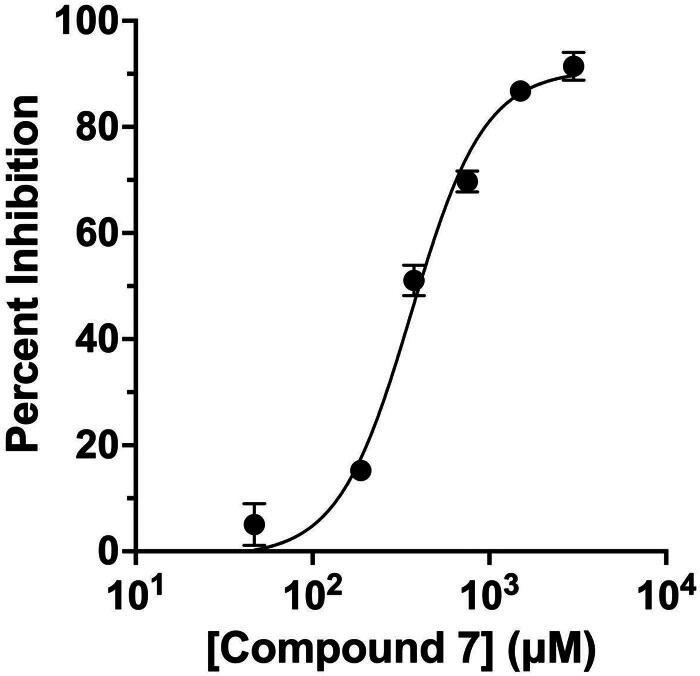
Inhibition of the interaction between RNAP β′CH and NusG by compound **7** measured by the protein complementation assay. The results are presented as *n* = 3, with variability shown as ± SE.

To confirm the impact of compound **7** on the interaction between the CH domain in the β′ subunit and NusG from the *Bacillus subtilis* strain 168, we utilised an internally developed competitive protein complementation assay to establish the 50% inhibition concentration (IC_50_) of the compound^22^. In this assay, NusG and β′CH were combined with two complementary fragments of the Nano-Luc luciferase enzyme. Introducing a specific inhibitor that targets the NusG-β′CH PPI impaired the efficient reformation of the natural luciferase complex. Consequently, the catalytic reaction of the reconstituted luciferase was impeded, leading to alterations in luminescence levels. These changes in luminescence were then used to observe the inhibitory activity induced by the compound. As depicted in Figure 6, compound **7** exhibited inhibitory activity against the NusG-β′CH protein-protein interaction, despite the challenge of experiments obtaining an IC_50_ value due to the compound’s low aqueous solubility. the IC_50_ of compound **7** in inhibiting the interaction between NusG and β′CH was 361 ± 23 µM, comparable to the affinity of β′CH binding to the native residues 19–34 (EGRVATSLREHIKLHN) of the *E. coli* NusG NTD^14^, and superior to the IC_50_ value of the hit compound **AW00783** (666 ± 53 µM)^14^. Note that the high IC_50_ values may be resulted from the nature of PPI inhibitors and the low solubility of lead compounds.

### Live-cell fluorescence imaging

To further confirm that compound **7** is a specific transcription inhibitor through targeting the NusG-β′CH interaction, we conducted fluorescence microscopy assay on a panel of reporter strains of *B. subtilis*, including BS23 (AtpA-GFP)^23^, BS128 (NusG-GFP)^24^, BS1048 (RpoC-GFP)^25^, and BS1049 (RpsB-GFP)^25^, labelling green fluorescence on cell membrane, NusG, RNAP, and ribosomes, respectively. Cells were treated with either control antibiotics or compound **7** at 1× MIC and observed using fluorescence microscopy after a 30-min incubation period. To visualise the nucleoid, 4′,6-diamidino-2-phenylindole (DAPI) was also added to a final concentration of 1 µg/mL.

**Figure 7.**
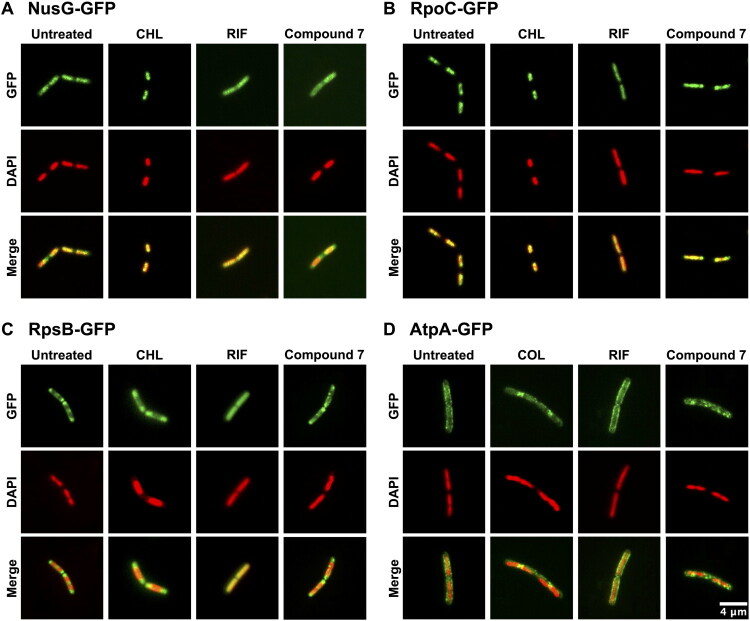
Fluorescent microscopy images of *B. subtilis* cells expressing (A) NusG-GFP, (B) RpoC-GFP, (C) RpsB-GFP, and (D) AtpA-GFP, in the presence of rifampicin (RIF), chloramphenicol (CHL), colistin (COL) and compound **7**. The images show GFP fluorescence (green), nucleoids stained with DAPI (red), and overlays of the GFP and DAPI signals. Scale bar: 4 µm.

Analysis of the green fluorescent protein (GFP) revealed that compound **7** disrupts bacterial transcription, as indicated by the delocalisation of NusG (Figure 7(A)) and RpoC (Figure 7(B)). These observed effects were in line with those observed using rifampicin, a known transcription inhibitor. The delocalisation of the fluorescence suggested that compound **7** interfered with transcription processes.

Interestingly, however, we observed distinct differences between rifampicin and compound **7** treatments in terms of nucleoid and ribosomal protein (RpsB) localisation (Figure 7(C)). Rifampicin treatment resulted in complete nucleoid expansion, causing extensive delocalisation of RpsB, and disrupted the normal mutual exclusivity between the nucleoid and RpsB localisation; this aligns with previous findings^26^. In comparison, chloramphenicol (a translation inhibitor) treatment induced nucleoid condensation, causing RpsB-GFP fluorescence to extend from polar regions towards the mid-cell region (Figure 7(C)). This highlights the distinct effects of transcription versus translation inhibitors on nucleoid architecture and ribosomal protein localisation. Compound **7** caused only mild nucleoid expansion accompanied by irregular nucleoid morphology in some cells. Large degree of delocalisation or redistribution towards cell midpoints was not observed for RpsB upon the treatment of compound **7** (Figure 7(C)).

To assess the potential impact of compound **7** on membrane morphology, we examined cells expressing membrane-localised AtpA-GFP^23^. Neither rifampicin nor compound **7** induced clear membrane invaginations (Figure 7(D)), indicating no direct membrane disruption. However, treatment with compound **7** resulted in mild membrane patches or clustering of AtpA-GFP fluorescence, a pattern not observed with rifampicin treatment. This was likely to be resulted indirectly from transcription inhibition: disruption of transcription elongation could lead to imbalanced expression or mislocalization of membrane-associated proteins, or the disruption could alter the local membrane microenvironment and causing AtpA clustering. In contrast, colistin, a known membrane-targeting antibiotic^27^, caused complete delocalisation of AtpA-GFP, consistent with direct membrane disruption.

Collectively, our microscopy analyses clearly showed that transcription inhibitors (such as rifampicin and compound **7**) induced nucleoid expansion, whereas translation inhibitors (such as chloramphenicol) caused nucleoid condensation (Figure 7). Compound **7** exhibited a mild nucleoid expansion and irregular nucleoid morphology, distinct from rifampicin-induced complete expansion. Furthermore, the unique nucleoid morphology, uneven RpsB distribution, and AtpA membrane patchiness caused by compound **7** suggested a different transcription inhibition mechanism than rifampicin, likely through specific interference with the NusG-RNAP interaction as designed.

### Cell-based transcription assay

**Figure 8.**
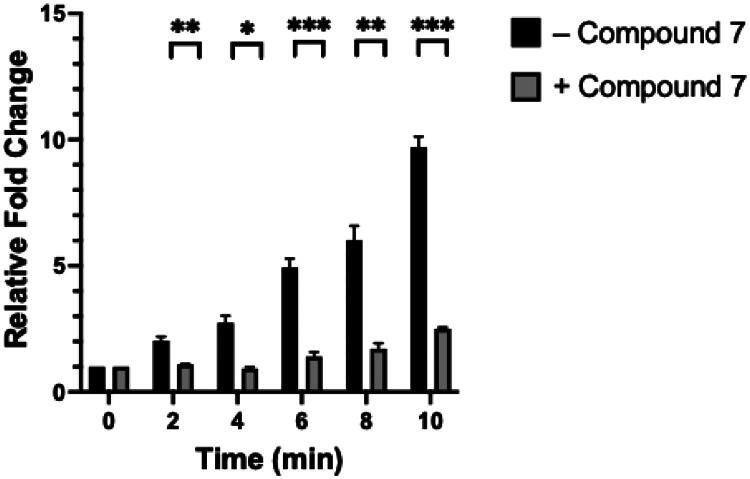
Effect of compound **7** on *Nluc* expression by luciferase-based bacterial reporter system. The results are presented as *n* = 3, with variability shown as ± SE. Statistical significance between treatment group and untreated control was assessed using the unpaired *t*-test, with *p* < 0.05 denoted by *, *p* < 0.01 denoted by **, and *p* < 0.001 denoted by ***.

After the validation of subcellular target by epifluorescence microscopy, we employed a previously established luciferase-based bacterial reporter system to evaluate the impact of compound **7** on transcriptional process^28^. In this assay, *B. subtilis* strain BS2019 carrying the *Nluc* gene under the control of the *P_xyl_* promoter was utilised. Inhibition of bacterial transcription by compound **7** would result in reduced *Nluc* expression. When the bacteria were exposed to compound 7 at ½× MIC, a concentration shown to cause no bacterial stress in a time-kill kinetic assay (Supplementary Figure), we observed a significant reduction in *Nluc* mRNA levels relative to the untreated control over 10 min (Figure 8). These results are consistent with previous findings for compound targeting the RNAP-σ factor interaction^28^, suggesting that compound **7** inhibits bacterial transcription.

### In silico ADME evaluation

Previously, we evaluated the drug-like properties of selected NusG compounds, which demonstrated excellent oral absorption and a low likelihood of cardiotoxicity^14^. In this study, five compounds (**3**, **6**, **7**, **9**, and **15**) with the highest antibiotic activity were subjected to *in silico* ADME evaluation using Schrödinger Maestro. The parameters calculated included the octanol-water partitioning coefficient (QPlogPo/w), aqueous solubility (QPlogS), binding to human serum albumin (QPlogKhsa), number of likely metabolic reactions (#metab), brain/blood partition coefficient (QPlogBB), central nervous system activity (CNS), apparent Caco-2 cell permeability (QPPCaco), human oral absorption, and predicted IC_50_ value for the blockage of HERG potassium channels (QPloghERG) (Table 9).

**Table 9.** *In silico* ADME evaluation of compounds **3**, **6**, **7**, **9**, **15**, and **JMC-38**.

Principle descriptors	JMC-38	3	6	7	9	15	Standard range**a**
QPlogPo/w	5.197	6.183	6.702	6.693	4.965	6.616	−2.0–6.5
QPlogS	−5.506	−7.828	−8.145	−8.191	−6.655	−7.967	−6.5–0.5
QPlogKhsa	0.520	0.853	1.060	1.060	0.760	1.049	−1.5–1.5
#metab	6	3	3	4	4	4	1–8
QPlogBB	0.430	0.014	0.107	0.088	−1.276	0.090	−3.0–1.2
CNS	1	1	1	1	−2	1	−2 (inactivity), +2 (activity)
QPPCaco	866.17	2492.81	3010.53	2905.79	343.24	2726.54	<25 poor, >500 great
(%) Human Oral Absorption	98.16	100.00	100.00	100.00	88.879	100.00	<25% poor, >80% high
QPlogHERG	−7.092	−7.127	−7.116	−7.162	−7.189	−7.105	< −5

^a^ Statistics of 95% of known drugs by Qikprop.

As shown in Table 9, although the lipophilicity of these compounds approaches the high end of drug-like molecules, the number of probable metabolic processes (#metab) and blood protein binding (QPlogKhsa) remained within the normal range, indicating that the selected chemical structures are drug-like. The predicted IC_50_ values for the blockage of HERG potassium channels (QPlogHERG) for all selected compounds were within the reference range, suggesting a low potential for cardiotoxicity. Additionally, the selected compounds demonstrated excellent oral absorption.

### Docking studies

**Figure 9.**
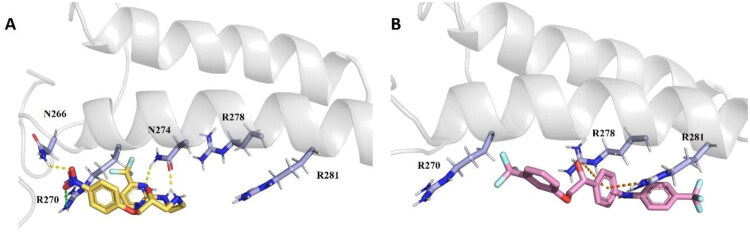
Molecular docking model of RNAP β′CH with A) the hit compound **AW00783** (yellow) and B) compound **7** (pink). Key residues of β′CH are highlighted in light blue. Hydrogen bonding, salt bridge and π-cation interactions are represented in yellow, green, and orange respectively.

*In silico* docking studies were performed using Autodock Vina to simulate the docking model of RNAP β′CH with the hit compound AW00783 and compound **7** (Figure 9). The structure of RNAP β′CH was retrieved from the *E. coli* RNA Polymerase complexed with NusG (PDB ID: 5TBZ). The receptor was prepared by adding hydrogen atoms and Gasteiger charges using the Dock Prep tool in Chimaera. Similarly, polar hydrogen atoms and Gasteiger charges were applied to the ligands. The binding site was defined based on key residues, specifically Arg270, Arg278, and Arg281, which are known to establish essential interactions with NusG. The centre point coordinates for the docking grid were set to X = 367.219, Y = −3.43665, and Z = 133.76, with grid dimensions of 18.52 Å,13.81 Å, 16.46 Å.

Figure 9(A) illustrates that the nitro group of **AW00783** forms a hydrogen bond with Asn266 and a salt bridge with Arg276. Additionally, Asn274 forms hydrogen bonds with the nitrogen atom of the pyrimidine ring and the linker NH group. As shown in Figure 9(B), the middle benzene ring engages a π-cation interaction with Arg278 and Arg281. Compound **7** demonstrates an improved binding affinity of −5.7 kcal/mol, as calculated by AutoDock Vina, compared to **AW00783**, which has a binding affinity of −4.4 kcal/mol. This suggests that Compound **7** has a more stable interaction with the receptor.

## Conclusion

In summary, our study addresses the urgent need for novel antimicrobials with unique mechanisms of action to combat emerging antimicrobial resistance. Building on our previous work with PPI inhibitors of bacteria transcription^29–31^, particularly targeting RNAP-σ^32–37^ and NusB-NusE^38–44^, we have now identified the interaction between the bacterial transcription factor NusG and RNAP β′CH as a viable target for antimicrobial development. Through scaffold hopping, we developed a novel series of triaryl compounds capable of disrupting the RNAP-NusG PPI, effectively inhibiting bacterial growth. Using a rational design approach guided by a pharmacophore model, we synthesised and tested a panel of compounds against Gram-positive bacteria. Notably, compounds **7** and **9** demonstrated significant antimicrobial activity, with MIC values as low as 1 µg/mL. Compound **7**, in particular, showed rapid bacteria-killing effects, reducing bacterial counts to undetectable levels within 30 min at concentrations of 1× MIC or higher. Fluorescent microscopy and cell-based transcription assays confirmed that these compounds share a similar mechanism of action with the established transcription inhibitor rifampicin, disrupting bacterial transcription by targeting the β′CH-NusG interaction. These findings highlight the potential of these triaryl antimicrobials for further drug development, offering a promising avenue for addressing the challenge of antimicrobial resistance. In addition, our findings suggest that other PPIs essential for bacterial transcription could also be explored as potential targets for novel antimicrobial drug discovery^45^^,^^46^. This approach not only broadens the landscape of possible interventions but also reinforces the potential of targeting transcriptional machinery as a viable strategy in the fight against resistant bacterial pathogens.

## Experimental section

### General

Chemicals and reagents utilised in synthesis, unless otherwise stated, were in commercial grade and required no purification. All reactions were monitored by thin-layer chromatography (TLC) on glass sheets (Silica gel F_254_) which were visualised under UV light. And flash chromatography purification was conducted by using silica gel (200–300 mesh) column.^1^H-NMR (400 MHz or 600 MHz),^13^C-NMR (100 MHz or 150 MHz), and ^19^F (565 MHz) spectra were measured on Bruker Advance-III spectrometer with TMS as an internal standard. Chemical shifts were presented in *δ* (ppm) and coupling constants (*J*) in Hz. High resolution mass spectrometry (HRMS) spectra were measured by Agilent 6540 liquid chromatography-electrospray ionisation (LC-EI) QTOF Mass spectrometer. The purity of all product compounds tested for biological activities was >95%, determined by analytical HPLC being performed on Waters HPLC system including 2535 quaternary gradient module, 2707 autosampler and 2998 photodiode array (PDA) detector with a XBridge C18, 4.6 × 100 mm, 5 *µ*M particle size.

General procedure for the synthesis of compound **C_1_**–**C_7_**, **C_13_**. To a solution of 2-chloropyridine or iodobenzene (1 equiv), 1–(4-aminophenyl)ethan-1-one (1.2 equiv) in toluene, Pd(OAc)_2_ (0.2 equiv), X-Phos (0.2 equiv) and Cs_2_CO_3_ (1.5 equiv) was added. The mixture was stirred under nitrogen at 120 °C for 8 h. After cooling to room temperature and filtration with diatomite, water was added and the filtrate was extracted with ethyl acetate for three times and washed with saturated brine. The combined organic layer was dried over anhydrous Na_2_SO_4_ and purified by column chromatography on silica gel to provide the titled compounds.

#### 1-(4-((5-(Trifluoromethyl)pyridin-2-yl)amino)phenyl)ethan-1-one (C1)

The titled compound was prepared from 2-chloro-5-(trifluoromethyl)pyridine (1 g, 5.51 mmol) and 1–(4-aminophenyl)ethan-1-one (893.5 mg, 6.61 mmol), purified by column chromatography on silica gel with the elution fluid of Hexane/EA (100:1–50:1). Pale yellow solid, 1.35 g, 88% yield. ^1^H NMR (600 MHz, Chloroform-*d*) *δ* 8.44 (d, *J* = 2.7 Hz, 1H), 8.02 (d, *J* = 8.6 Hz, 1H), 7.60 (d, *J* = 8.4 Hz, 2H), 7.53 (dd, *J* = 8.7, 2.7 Hz, 1H), 7.25 (d, *J* = 8.3 Hz, 2H), 6.64–6.53 (m, 1H), 2.70 (s, 3H).

#### 1-(4-((4-(Trifluoromethyl)phenyl)amino)phenyl)ethan-1-one (C2)

The titled compound was prepared from 1-iodo-4-(trifluoromethyl)benzene (1 g, 3.68 mmol) and 1–(4-aminophenyl)ethan-1-one (596.3 mg, 4.41 mmol), purified by column chromatography on silica gel with the elution fluid of Hexane/EA (200:1–100:1). Pale yellow solid, 867.3 mg, 84% yield. ^1^H NMR (600 MHz, Chloroform-*d*) *δ* 7.96–7.92 (m, 2H), 7.58 (d, *J* = 8.3 Hz, 2H), 7.24 (d, *J* = 8.3 Hz, 2H), 7.15–7.11 (m, 2H), 6.47 (s, 1H), 2.58 (s, 3H).

#### 1-(4-((3-(Trifluoromethyl)phenyl)amino)phenyl)ethan-1-one (C3)

The titled compound was prepared from 1-iodo-3-(trifluoromethyl)benzene (1 g, 3.68 mmol) and 1–(4-aminophenyl)ethan-1-one (596.3 mg, 4.41 mmol), purified by column chromatography on silica gel with the elution fluid of Hexane/EA (200:1–100:1). Pale yellow solid, 889.9 mg, 86% yield. ^1^H NMR (400 MHz, Chloroform-*d*) *δ* 7.96–7.91 (m, 2H), 7.49–7.42 (m, 2H), 7.38–7.30 (m, 2H), 7.10–7.04 (m, 2H), 6.21 (s, 1H), 2.58 (s, 3H).

#### 1-(4-((2-(Trifluoromethyl)phenyl)amino)phenyl)ethan-1-one (C4)

The titled compound was prepared from 1-iodo-2-(trifluoromethyl)benzene (1 g, 3.68 mmol) and 1–(4-aminophenyl)ethan-1-one (596.3 mg, 4.41 mmol), purified by column chromatography on silica gel with the elution fluid of Hexane/EA (200:1–100:1). Pale yellow solid, 869.3 mg, 84% yield. ^1^H NMR (400 MHz, Chloroform-*d*) *δ* 7.77 (dd, *J* = 8.6, 1.3 Hz, 2H), 7.55 (d, *J* = 7.9 Hz, 1H), 7.42–7.37 (m, 2H), 7.07 (dt, *J* = 8.4, 4.1 Hz, 1H), 6.90 (dd, *J* = 8.6, 1.3 Hz, 2H), 6.56 (s, 1H), 2.43 (d, *J* = 1.2 Hz, 3H).

#### 1-(4-((4-methoxyphenyl)amino)phenyl)ethan-1-one (C5)

The titled compound was prepared from 1-iodo-4-methoxybenzene (1 g, 4.27 mmol) and 1–(4-aminophenyl)ethan-1-one (693.1 mg, 5.13 mmol), purified by column chromatography on silica gel with the elution fluid of Hexane/EA (200:1–100:1). Pale yellow solid, 787.9 mg, 77% yield. ^1^H NMR (400 MHz, Chloroform-*d*) *δ* 7.89–7.81 (m, 2H), 7.19–7.14 (m, 2H), 6.96–6.91 (m, 2H), 6.86–6.81 (m, 2H), 6.05–5.97 (m, 1H), 3.85 (s, 3H), 2.53 (s, 3H).

#### 1-(4-((4-(Methylthio)phenyl)amino)phenyl)ethan-1-one (C6)

The titled compound was prepared from (4-iodophenyl)(methyl)sulfane (1 g, 4 mmol) and 1–(4-aminophenyl)ethan-1-one (648.5 mg, 4.8 mmol), purified by column chromatography on silica gel with the elution fluid of Hexane/EA (200:1–100:1). Pale yellow solid, 748.8 mg, 73% yield. ^1^H NMR (400 MHz, Chloroform-*d*) *δ* 7.88–7.83 (m, 2H), 7.29–7.24 (m, 2H), 7.14–7.10 (m, 2H), 7.01 (s, 1H), 6.98–6.94 (m, 2H), 6.73 (s, 1H), 2.54 (s, 3H), 2.49 (s, 3H).

#### 2-((4-Acetylphenyl)amino)benzonitrile (C7)

The titled compound was prepared from 2-iodobenzonitrile (1 g, 4.37 mmol) and 1–(4-aminophenyl)ethan-1-one (708.2 mg, 5.24 mmol), purified by column chromatography on silica gel with the elution fluid of Hexane/EA (100:1–50:1). Pale yellow solid, 672.6 mg, 65% yield. ^1^H NMR (400 MHz, Chloroform-*d*) *δ* 8.00–7.96 (m, 2H), 7.61 (dd, *J* = 7.7, 1.5 Hz, 1H), 7.54–7.44 (m, 2H), 7.20 (d, *J* = 8.7 Hz, 2H), 7.07–7.02 (m, 1H), 6.55 (s, 1H), 2.60 (s, 3H).

#### 1-(3-((2-(Trifluoromethyl)phenyl)amino)phenyl)ethan-1-one (C13)

The titled compound was prepared from 1-iodo-2-(trifluoromethyl)benzene (1 g, 3.68 mmol) and 1–(3-aminophenyl)ethan-1-one (596.3 mg, 4.41 mmol), purified by column chromatography on silica gel with the elution fluid of Hexane/EA (200:1–100:1). Pale yellow solid, 855.9 mg, 83% yield. ^1^H NMR (400 MHz, Chloroform-*d*) *δ* 7.69 (t, *J* = 2.0 Hz, 1H), 7.61 (m, *J* = 6.3, 4.4, 3.1 Hz, 2H), 7.46–7.39 (m, 2H), 7.36 (d, *J* = 8.3 Hz, 1H), 7.31 (m, *J* = 8.0, 2.4, 1.1 Hz, 1H), 7.04 (t, *J* = 7.6 Hz, 1H), 6.15 (s, 1H), 2.62 (s, 3H).

General procedure for the synthesis of compound **D_1_**–**D_7_, D_13_**. To a solution of selected compound **C_1_–C_7_, C_13_** (1 equiv) in a mixture of CHCl_3_ and EA (1:1), CuBr_2_ (1.1 equiv) was added. The mixture was stirred at 60 °C and monitored to react completely. After cooling to room temperature and filtration with diatomite, the filtrate was concentrated and purified by column chromatography on silica gel to provide the titled compounds.

#### 2-Bromo-1-(4-((5-(trifluoromethyl)pyridin-2-yl)amino)phenyl)ethan-1-one (D1)

The titled compound was prepared from **C_1_** (500 mg, 1.78 mmol) and purified by column chromatography on silica gel with the elution fluid of Hexane/EA (100:1–50:1). Yellow solid, 316.5 mg, 49% yield. ^1^H NMR (600 MHz, Chloroform-*d*) *δ* 8.57 (s, 1H), 8.04–8.01 (m, 2H), 7.80 (dd, *J* = 8.8, 2.4 Hz, 1H), 7.65–7.62 (m, 2H), 7.16 (s, 1H), 6.97 (dd, *J* = 8.9, 4.3 Hz, 1H), 4.45 (s, 2H).

#### 2-Bromo-1-(4-((4-(trifluoromethyl)phenyl)amino)phenyl)ethan-1-one (D2)

The titled compound was prepared from **C_2_** (500 mg, 1.79 mmol) and purified by column chromatography on silica gel with the elution fluid of Hexane/EA (200:1–150:1). Yellow solid, 284.1 mg, 44% yield. ^1^H NMR (400 MHz, Chloroform-*d*) *δ* 7.97 (d, *J* = 8.4 Hz, 2H), 7.61 (d, *J* = 8.3 Hz, 2H), 7.27 (d, *J* = 8.0 Hz, 2H), 7.14 (d, *J* = 8.5 Hz, 2H), 6.34 (s, 1H), 4.41 (s, 2H).

#### 2-Bromo-1-(4-((3-(trifluoromethyl)phenyl)amino)phenyl)ethan-1-one (D3)

The titled compound was prepared from **C_3_** (500 mg, 1.79 mmol) and purified by column chromatography on silica gel with the elution fluid of Hexane/EA (200:1–150:1). Yellow solid, 262.3 mg, 41% yield. ^1^H NMR (400 MHz, Chloroform-*d*) *δ* 7.97 (d, *J* = 2.0 Hz, 1H), 7.95 (d, *J* = 2.1 Hz, 1H), 7.52–7.44 (m, 2H), 7.41–7.34 (m, 2H), 7.07 (d, *J* = 2.0 Hz, 1H), 7.06 (d, *J* = 2.0 Hz, 1H), 6.27 (s, 1H), 4.41 (s, 2H).

#### 2-Bromo-1-(4-((2-(trifluoromethyl)phenyl)amino)phenyl)ethan-1-one (D4)

The titled compound was prepared from **C_4_** (500 mg, 1.79 mmol) and purified by column chromatography on silica gel with the elution fluid of Hexane/EA (200:1–150:1). Yellow solid, 278.9 mg, 44% yield. ^1^H NMR (400 MHz, Chloroform-*d*) *δ* 8.24 (d, *J* = 2.1 Hz, 1H), 7.81 (dt, *J* = 8.7, 2.1 Hz, 1H), 7.75 (dd, *J* = 8.0, 1.5 Hz, 1H), 7.63–7.57 (m, 1H), 7.54 (d, *J* = 8.2 Hz, 1H), 7.35–7.30 (m, 1H), 7.07 (dd, *J* = 8.6, 1.9 Hz, 1H), 6.89 (s, 1H), 4.36 (d, *J* = 2.0 Hz, 2H).

#### 2-Bromo-1-(4-((4-methoxyphenyl)amino)phenyl)ethan-1-one (D5)

The titled compound was prepared from **C_5_** (500 mg, 2.07 mmol) and purified by column chromatography on silica gel with the elution fluid of Hexane/EA (200:1–150:1). Yellow solid, 244.2 mg, 37% yield. ^1^H NMR (600 MHz, Chloroform-*d*) *δ* 7.78 (d, *J* = 8.7 Hz, 2H), 7.10–7.06 (m, 2H), 6.87–6.83 (m, 2H), 6.76–6.72 (m, 2H), 4.28 (s, 2H), 3.76 (s, 3H).

#### 2-Bromo-1-(4-((4-(methylthio)phenyl)amino)phenyl)ethan-1-one (D6)

The titled compound was prepared from **C_6_** (500 mg, 1.94 mmol), and it was directly used in the next step without further purification.

#### 2-((4-(2-Bromoacetyl)phenyl)amino)benzonitrile (D7)

The titled compound was prepared from **C_7_** (500 mg, 2.12 mmol) and purified by column chromatography on silica gel with the elution fluid of Hexane/EA (100:1–50:1). Yellow solid, 259.4 mg, 39% yield. ^1^H NMR (400 MHz, Chloroform-*d*) *δ* 7.99–7.94 (m, 2H), 7.78–7.75 (m, 1H), 7.67–7.64 (m, 1H), 7.47 (td, *J* = 7.7, 1.2 Hz, 1H), 7.39–7.35 (m, 3H), 4.43 (s, 2H).

#### 2-Bromo-1-(3-((2-(trifluoromethyl)phenyl)amino)phenyl)ethan-1-one (D13)

The titled compound was prepared from **C_13_** (500 mg, 1.79 mmol) and purified by column chromatography on silica gel with the elution fluid of Hexane/EA (200:1–150:1). Yellow solid, 283.4 mg, 44% yield. ^1^H NMR (600 MHz, Chloroform-*d*) *δ* 7.71 (t, *J* = 2.0 Hz, 1H), 7.65–7.60 (m, 2H), 7.45 (q, *J* = 8.1 Hz, 2H), 7.39 (d, *J* = 8.3 Hz, 1H), 7.33 (dd, *J* = 8.1, 2.4 Hz, 1H), 7.07 (t, *J* = 7.6 Hz, 1H), 6.16 (s, 1H), 4.45 (s, 2H).

General procedure for the synthesis of compound **E_1_**–**E_19_**, **E_23_**–**E_24_**, **E_27_**. To a solution of phenol or pyridinol (1.5 equiv) in acetone, K_2_CO_3_ (2 equiv) was added. The mixture was stirred at room temperature for 30 min. With the following adding of selected compound **D_1_–D_7_, D_13_** (1 equiv), it was stirred at reflux for 6 h and monitored to react completely. After cooling to room temperature and filtration, the filtrate was concentrated and purified by column chromatography on silica gel to provide the titled compounds.

#### 2-(4-Bromo-2,6-difluorophenoxy)-1-(4-((5-(trifluoromethyl)pyridin-2-yl)amino)phenyl)ethan-1-one (E1)

The titled compound was prepared from **D_1_** (100 mg, 0.28 mmol) and 4-bromo-2,6-difluorophenol (87.3 mg, 0.42 mmol), purified by column chromatography on silica gel with the elution fluid of Hexane/EA (20:1–10:1). Pale yellow solid, 122.4 mg, 90% yield. ^1^H NMR (600 MHz, DMSO-*d*_6_) *δ* 10.13 (s, 1H), 8.62–8.58 (m, 1H), 7.97 (dd, *J* = 8.9, 2.4 Hz, 1H), 7.91 (q, *J* = 9.0 Hz, 4H), 7.50 (dd, *J* = 8.4, 1.4 Hz, 2H), 7.07 (d, *J* = 8.9 Hz, 1H), 5.64 (s, 2H).

#### 2-(3,4-Difluorophenoxy)-1-(4-((5-(trifluoromethyl)pyridin-2-yl)amino)phenyl)ethan-1-one (E2)

The titled compound was prepared from **D_1_** (100 mg, 0.28 mmol) and 3,4-difluorophenol (54.3 mg, 0.42 mmol), purified by column chromatography on silica gel with the elution fluid of Hexane/EA (20:1–10:1). Pale yellow solid, 103.8 mg, 91% yield. ^1^H NMR (600 MHz, DMSO-*d*_6_) *δ* 10.12 (s, 1H), 8.61 (d, *J* = 2.4 Hz, 1H), 8.01–7.96 (m, 3H), 7.91 (d, *J* = 8.6 Hz, 2H), 7.35 (q, *J* = 9.6 Hz, 1H), 7.15 (m, *J* = 12.7, 6.7, 3.0 Hz, 1H), 7.07 (d, *J* = 8.9 Hz, 1H), 6.82 (dd, *J* = 8.6, 4.2 Hz, 1H), 5.54 (s, 2H).

#### 2-(4-(Trifluoromethyl)phenoxy)-1-(4-((5-(trifluoromethyl)pyridin-2-yl)amino)phenyl)ethan-1-one (E3)

The titled compound was prepared from **D_1_** (100 mg, 0.28 mmol) and 4-(trifluoromethyl)phenol (67.7 mg, 0.42 mmol), purified by column chromatography on silica gel with the elution fluid of Hexane/EA (20:1–10:1). Pale yellow solid, 111.1 mg, 91% yield. ^1^H NMR (600 MHz, DMSO-*d*_6_) *δ* 10.13 (s, 1H), 8.60 (d, *J* = 2.5 Hz, 1H), 8.02–7.95 (m, 3H), 7.92 (d, *J* = 8.8 Hz, 2H), 7.64 (d, *J* = 8.5 Hz, 2H), 7.14 (d, *J* = 8.5 Hz, 2H), 7.08 (d, *J* = 8.9 Hz, 1H), 5.65 (s, 2H).

#### 2-(4-Bromo-2,6-difluorophenoxy)-1-(4-((4-(trifluoromethyl)phenyl)amino)phenyl)ethan-1-one (E4)

The titled compound was prepared from **D_2_** (100 mg, 0.28 mmol) and 4-bromo-2,6-difluorophenol (87.5 mg, 0.42 mmol), purified by column chromatography on silica gel with the elution fluid of Hexane/EA (50:1–20:1). Pale yellow solid, 123.3 mg, 91% yield. ^1^H NMR (400 MHz, Chloroform-*d*) *δ* 7.94–7.89 (m, 2H), 7.60 (d, *J* = 8.5 Hz, 2H), 7.25 (d, *J* = 8.4 Hz, 2H), 7.16–7.08 (m, 4H), 6.37 (s, 1H), 5.40 (s, 2H).

#### 2-(3,4-Difluorophenoxy)-1-(4-((4-(trifluoromethyl)phenyl)amino)phenyl)ethan-1-one (E5)

The titled compound was prepared from **D_2_** (100 mg, 0.28 mmol) and 3,4-difluorophenol (54.5 mg, 0.42 mmol), purified by column chromatography on silica gel with the elution fluid of Hexane/EA (50:1–20:1). Pale yellow solid, 100.8 mg, 89% yield. ^1^H NMR (400 MHz, Chloroform-*d*) *δ* 7.96 (d, *J* = 8.5 Hz, 2H), 7.61 (d, *J* = 8.3 Hz, 2H), 7.26 (d, *J* = 8.4 Hz, 2H), 7.15 (d, *J* = 8.5 Hz, 2H), 7.08 (q, *J* = 9.4 Hz, 1H), 6.79 (m, *J* = 11.8, 6.4, 3.0 Hz, 1H), 6.68–6.63 (m, 1H), 6.42 (s, 1H), 5.21 (s, 2H).

#### 2-(4-(Trifluoromethyl)phenoxy)-1-(4-((4-(trifluoromethyl)phenyl)amino)phenyl)ethan-1-one (E6/26)

The titled compound was prepared from **D_2_** (100 mg, 0.28 mmol) and 4-(trifluoromethyl)phenol (67.9 mg, 0.42 mmol), purified by column chromatography on silica gel with the elution fluid of Hexane/EA (50:1–20:1). Pale yellow solid, 111.6 mg, 91% yield, mp 126–127 °C. ^1^H NMR (400 MHz, Chloroform-*d*) *δ* 7.99 (d, *J* = 8.7 Hz, 2H), 7.59 (dd, *J* = 17.4, 8.4 Hz, 4H), 7.27 (d, *J* = 8.0 Hz, 2H), 7.18–7.12 (m, 2H), 7.02 (d, *J* = 8.5 Hz, 2H), 6.35 (s, 1H), 5.30 (s, 2H). ^13^C NMR (151 MHz, Chloroform-*d*) *δ* 191.72 160.5, 147.5, 143.7, 130.5, 127.2, 127.0, 127.0, 127.0, 126.9, 126.9, 125.2, 123.8, 123.6, 123.4, 118.9, 118.8, 116.0, 116.0, 114.8, 114.7, 70.5, 29.7. HRMS(ESI): calcd for C_22_H_15_F_6_NO, [M - H]^−^ 438.0929; found, 438.0938. HPLC purity: 99.06%.

#### 2-(3-(Trifluoromethyl)phenoxy)-1-(4-((4-(trifluoromethyl)phenyl)amino)phenyl)ethan-1-one (E7)

The titled compound was prepared from **D_2_** (100 mg, 0.28 mmol) and 3-(trifluoromethyl)phenol (67.9 mg, 0.42 mmol), purified by column chromatography on silica gel with the elution fluid of Hexane/EA (50:1–20:1). Pale yellow solid, 110 mg, 90% yield. ^1^H NMR (400 MHz, Chloroform-*d*) *δ* 8.04–7.96 (m, 2H), 7.61 (d, *J* = 8.3 Hz, 2H), 7.42 (t, *J* = 8.0 Hz, 1H), 7.27 (d, *J* = 8.3 Hz, 3H), 7.24–7.10 (m, 4H), 6.36 (s, 1H), 5.28 (s, 2H).

#### 2-(2-(Trifluoromethyl)phenoxy)-1-(4-((4-(trifluoromethyl)phenyl)amino)phenyl)ethan-1-one (E8)

The titled compound was prepared from **D_2_** (100 mg, 0.28 mmol) and 2-(trifluoromethyl)phenol (67.9 mg, 0.42 mmol), purified by column chromatography on silica gel with the elution fluid of Hexane/EA (50:1–20:1). Pale yellow solid, 109.4 mg, 89% yield. ^1^H NMR (400 MHz, Chloroform-*d*) *δ* 8.05–7.99 (m, 2H), 7.63–7.57 (m, 3H), 7.49–7.43 (m, 1H), 7.25 (d, *J* = 8.3 Hz, 2H), 7.15–7.09 (m, 2H), 7.05 (t, *J* = 7.6 Hz, 1H), 6.94 (d, *J* = 8.4 Hz, 1H), 6.40 (d, *J* = 5.0 Hz, 1H), 5.29 (s, 2H).

#### 2-(4-Nitrophenoxy)-1-(4-((4-(trifluoromethyl)phenyl)amino)phenyl)ethan-1-one (E9)

The titled compound was prepared from **D_2_** (100 mg, 0.28 mmol) and 4-nitrophenol (58.3 mg, 0.42 mmol), purified by column chromatography on silica gel with the elution fluid of Hexane/EA (50:1–20:1). Yellow solid, 108.6 mg, 93% yield. ^1^H NMR (400 MHz, Chloroform-*d*) *δ* 8.27–8.18 (m, 2H), 7.97 (d, *J* = 8.7 Hz, 2H), 7.62 (d, *J* = 8.3 Hz, 2H), 7.26 (s, 2H), 7.20–7.11 (m, 2H), 7.08–6.96 (m, 2H), 6.36 (s, 1H), 5.38 (s, 2H).

#### 4-(2-Oxo-2-(4-((4-(trifluoromethyl)phenyl)amino)phenyl)ethoxy)benzonitrile (E10)

The titled compound was prepared from **D_2_** (100 mg, 0.28 mmol) and 4-hydroxybenzonitrile (49.9 mg, 0.42 mmol), purified by column chromatography on silica gel with the elution fluid of Hexane/EA (20:1–10:1). Pale yellow solid, 104.3 mg, 94% yield. ^1^H NMR (600 MHz, Chloroform-*d*) *δ* 7.96 (d, *J* = 8.2 Hz, 2H), 7.61 (dd, *J* = 8.3, 5.2 Hz, 4H), 7.27 (d, *J* = 8.1 Hz, 2H), 7.15 (d, *J* = 8.4 Hz, 2H), 7.00 (d, *J* = 8.6 Hz, 2H), 6.45 (s, 1H), 5.33 (s, 2H).

#### 2-(4-(Methylthio)phenoxy)-1-(4-((4-(trifluoromethyl)phenyl)amino)phenyl)ethan-1-one (E11)

The titled compound was prepared from **D_2_** (100 mg, 0.28 mmol) and 4-(methylthio)phenol (58.7 mg, 0.42 mmol), purified by column chromatography on silica gel with the elution fluid of Hexane/EA (50:1–20:1). Pale yellow solid, 104.1 mg, 89% yield. ^1^H NMR (400 MHz, Chloroform-*d*) *δ* 8.02–7.97 (m, 2H), 7.60 (d, *J* = 8.4 Hz, 2H), 7.28–7.23 (m, 4H), 7.16–7.12 (m, 2H), 6.94–6.89 (m, 2H), 6.33 (s, 1H), 5.21 (s, 2H), 2.46 (s, 3H).

#### 1-(4-((4-(Trifluoromethyl)phenyl)amino)phenyl)-2-((5-(trifluoromethyl)pyridin-2-yl)oxy)ethan-1-one (E12)

The titled compound was prepared from **D_2_** (100 mg, 0.28 mmol) and 5-(trifluoromethyl)pyridin-2-ol (68.3 mg, 0.42 mmol), purified by column chromatography on silica gel with the elution fluid of Hexane/EA (20:1–10:1). Pale yellow solid, 112.5 mg, 92% yield. ^1^H NMR (400 MHz, DMSO-*d*_6_) *δ* 9.38 (s, 1H), 8.38 (s, 1H), 7.99 (d, *J* = 8.6 Hz, 2H), 7.76 (d, *J* = 7.7 Hz, 1H), 7.66 (d, *J* = 8.4 Hz, 2H), 7.37 (d, *J* = 8.4 Hz, 2H), 7.27 (d, *J* = 8.5 Hz, 2H), 6.60 (d, *J* = 9.7 Hz, 1H), 5.50 (s, 2H).

#### 1-(4-((4-(Trifluoromethyl)phenyl)amino)phenyl)-2-((4-(trifluoromethyl)pyridin-2-yl)oxy)ethan-1-one (E13)

The titled compound was prepared from **D_2_** (100 mg, 0.28 mmol) and 4-(trifluoromethyl)pyridin-2-ol (68.3 mg, 0.42 mmol), purified by column chromatography on silica gel with the elution fluid of Hexane/EA (20:1–10:1). Pale yellow solid, 114.7 mg, 93% yield. ^1^H NMR (600 MHz, DMSO-*d*_6_) *δ* 9.39 (s, 1H), 8.01–7.98 (m, 2H), 7.93 (d, *J* = 7.0 Hz, 1H), 7.66 (d, *J* = 8.4 Hz, 2H), 7.36 (d, *J* = 8.4 Hz, 2H), 7.29–7.26 (m, 2H), 6.85 (d, *J* = 2.1 Hz, 1H), 6.58 (dd, *J* = 7.1, 2.0 Hz, 1H), 5.51 (s, 2H).

#### 1-(4-((4-(Trifluoromethyl)phenyl)amino)phenyl)-2-((5-(trifluoromethyl)pyridin-3-yl)oxy)ethan-1-one (E14)

The titled compound was prepared from **D_2_** (100 mg, 0.28 mmol) and 5-(trifluoromethyl)pyridin-3-ol (68.3 mg, 0.42 mmol), purified by column chromatography on silica gel with the elution fluid of Hexane/EA (20:1–10:1). Pale yellow solid, 110.5 mg, 90% yield. ^1^H NMR (400 MHz, Chloroform-*d*) *δ* 8.54 (d, *J* = 2.3 Hz, 2H), 7.95 (d, *J* = 8.5 Hz, 2H), 7.61 (d, *J* = 8.3 Hz, 2H), 7.45–7.41 (m, 1H), 7.26 (s, 2H), 7.16 (d, *J* = 8.6 Hz, 2H), 6.61 (t, *J* = 8.3 Hz, 1H), 5.39 (s, 2H).

#### 2-(4-(Trifluoromethyl)phenoxy)-1-(4-((3-(trifluoromethyl)phenyl)amino)phenyl)ethan-1-one (E15)

The titled compound was prepared from **D_3_** (100 mg, 0.28 mmol) and 4-(trifluoromethyl)phenol (67.9 mg, 0.42 mmol), purified by column chromatography on silica gel with the elution fluid of Hexane/EA (50:1–20:1). Pale yellow solid, 110.5 mg, 90% yield. ^1^H NMR (400 MHz, Chloroform-*d*) *δ* 7.94 (dd, *J* = 14.8, 8.6 Hz, 2H), 7.54 (d, *J* = 8.5 Hz, 1H), 7.45 (dd, *J* = 15.2, 7.5 Hz, 2H), 7.40–7.28 (m, 2H), 7.11–6.98 (m, 3H), 5.31 (s, 1H), 2.58 (s, 2H).

#### 2-(4-(Trifluoromethyl)phenoxy)-1-(4-((2-(trifluoromethyl)phenyl)amino)phenyl)ethan-1-one (E16)

The titled compound was prepared from **D_4_**(100 mg, 0.28 mmol) and 4-(trifluoromethyl)phenol (67.9 mg, 0.42 mmol), purified by column chromatography on silica gel with the elution fluid of Hexane/EA (50:1–20:1). Pale yellow solid, 113.6 mg, 93% yield. ^1^H NMR (400 MHz, Chloroform-*d*) *δ* 7.99–7.93 (m, 2H), 7.70 (d, *J* = 7.8 Hz, 1H), 7.59–7.52 (m, 4H), 7.23 (m, *J* = 8.3, 4.4 Hz, 1H), 7.03 (dd, *J* = 11.1, 8.5 Hz, 4H), 6.34 (s, 1H), 5.29 (s, 2H).

#### 1-(4-((4-Methoxyphenyl)amino)phenyl)-2-(4-(trifluoromethyl)phenoxy)ethan-1-one (E17)

The titled compound was prepared from **D_5_**(100 mg, 0.31 mmol) and 4-(trifluoromethyl)phenol (75.9 mg, 0.47 mmol), purified by column chromatography on silica gel with the elution fluid of Hexane/EA (50:1–20:1). Pale yellow solid, 111 mg, 89% yield. ^1^H NMR (400 MHz, Chloroform-*d*) *δ* 7.90–7.83 (m, 2H), 7.53 (d, *J* = 8.5 Hz, 2H), 7.18–7.12 (m, 2H), 6.99 (d, *J* = 8.5 Hz, 2H), 6.95–6.90 (m, 2H), 6.85–6.79 (m, 2H), 6.01 (s, 1H), 5.24 (s, 2H), 3.83 (s, 3H).

#### 1-(4-((4-(Methylthio)phenyl)amino)phenyl)-2-(4-(trifluoromethyl)phenoxy)ethan-1-one (E18)

The titled compound was prepared from **D_6_**(100 mg, 0.3 mmol) and 4-(trifluoromethyl)phenol (72.3 mg, 0.45 mmol), purified by column chromatography on silica gel with the elution fluid of Hexane/EA (50:1–20:1). Yellow solid, 115.1 mg, 93% yield. ^1^H NMR (400 MHz, Chloroform-*d*) *δ* 7.95–7.90 (m, 2H), 7.56 (d, *J* = 8.5 Hz, 2H), 7.31 (d, *J* = 8.5 Hz, 2H), 7.16 (d, *J* = 8.4 Hz, 2H), 7.00 (dd, *J* = 13.8, 8.6 Hz, 4H), 6.13 (s, 1H), 5.28 (s, 2H), 2.52 (s, 3H).

#### 2-((4-(2-(4-(Trifluoromethyl)phenoxy)acetyl)phenyl)amino)benzonitrile (E19)

The titled compound was prepared from **D_7_**(100 mg, 0.32 mmol) and 4-(trifluoromethyl)phenol (77.2 mg, 0.47 mmol), purified by column chromatography on silica gel with the elution fluid of Hexane/EA (20:1–10:1). Pale yellow solid, 116.1 mg, 92% yield. ^1^H NMR (400 MHz, Chloroform-*d*) *δ* 8.04–7.98 (m, 2H), 7.64 (dd, *J* = 7.9, 1.5 Hz, 1H), 7.59–7.48 (m, 4H), 7.23–7.19 (m, 2H), 7.13–7.07 (m, 1H), 7.02 (d, *J* = 8.6 Hz, 2H), 6.66 (s, 1H), 5.31 (s, 2H).

#### 2-((4-(Trifluoromethyl)benzyl)oxy)-1-(4-((4-(trifluoromethyl)phenyl)amino)phenyl)ethan-1-one (E23)

The titled compound was prepared from **D_2_**(100 mg, 0.28 mmol) and (4-(trifluoromethyl)phenyl)methanol (73.8 mg, 0.42 mmol), purified by column chromatography on silica gel with the elution fluid of Hexane/EA (50:1–20:1). Pale yellow solid, 114.8 mg, 91% yield. ^1^H NMR (600 MHz, Chloroform-*d*) *δ* 7.85–7.81 (m, 2H), 7.55 (d, *J* = 8.0 Hz, 2H), 7.50 (d, *J* = 8.3 Hz, 2H), 7.45 (d, *J* = 8.0 Hz, 2H), 7.15 (d, *J* = 8.3 Hz, 2H), 7.04–7.02 (m, 2H), 6.22 (s, 1H), 4.68 (d, *J* = 2.5 Hz, 4H).

#### 1-(4-((4-(Trifluoromethyl)phenyl)amino)phenyl)-2-((4-(trifluoromethyl)phenyl)thio)ethan-1-one (E24)

The titled compound was prepared from **D_2_**(100 mg, 0.28 mmol) and 4-(trifluoromethyl)benzenethiol (74.6 mg, 0.42 mmol), purified by column chromatography on silica gel with the elution fluid of Hexane/EA (50:1–20:1). Pale yellow solid, 103.2 mg, 81% yield. ^1^H NMR (400 MHz, Chloroform-*d*) *δ* 7.98–7.93 (m, 2H), 7.61 (d, *J* = 8.4 Hz, 2H), 7.54 (d, *J* = 8.4 Hz, 2H), 7.48 (d, *J* = 8.3 Hz, 2H), 7.27–7.24 (m, 2H), 7.15–7.12 (m, 2H), 6.32 (s, 1H), 4.33 (s, 2H).

#### 2-(4-(Trifluoromethyl)phenoxy)-1-(3-((2-(trifluoromethyl)phenyl)amino)phenyl)ethan-1-one (E27)

The titled compound was prepared from **D_13_**(100 mg, 0.28 mmol) and 4-(trifluoromethyl)phenol (67.9 mg, 0.42 mmol), purified by column chromatography on silica gel with the elution fluid of Hexane/EA (50:1–20:1). Pale yellow solid, 113.1 mg, 92% yield. ^1^H NMR (400 MHz, Chloroform-*d*) *δ* 7.70 (t, *J* = 2.0 Hz, 1H), 7.66–7.55 (m, 4H), 7.45 (td, *J* = 7.8, 2.9 Hz, 2H), 7.40–7.34 (m, 2H), 7.08 (t, *J* = 7.5 Hz, 1H), 7.01 (d, *J* = 8.5 Hz, 2H), 6.16 (s, 1H), 5.34 (s, 2H).

General procedure for the synthesis of compound **1** to **19, 23, 24, 31**. To a solution of selected compounds **E_1_–E_19_**, **E_23_–E_24_**, **E_27_** (1 equiv) in MeOH, NaBH_4_ (3 equiv) was added in ice bath. After warming to room temperature, the mixture was stirred for 8 h and monitored to react completely. It was concentrated and purified by column chromatography on silica gel to provide the titled compounds.

#### 2-(4-Bromo-2,6-difluorophenoxy)-1-(4-((5-(trifluoromethyl)pyridin-2-yl)amino)phenyl)ethan-1-ol (1)

The titled compound was prepared from **E_1_** (50 mg, 0.1 mmol) and purified by column chromatography on silica gel with the elution fluid of Hexane/EA (2:1–1:1). Pale yellow solid, 38.3 mg, 76% yield, mp 90–92 °C. ^1^H NMR (400 MHz, Chloroform-*d*) *δ* 8.46 (s, 1H), 7.69 (dd, *J* = 9.0, 2.5 Hz, 1H), 7.47–7.36 (m, 4H), 7.15 (t, *J* = 6.1 Hz, 2H), 6.94 (s, 1H), 6.85 (d, *J* = 8.6 Hz, 1H), 5.14–5.05 (m, 1H), 4.30 (dd, *J* = 10.3, 3.2 Hz, 1H), 4.13 (t, *J* = 9.5 Hz, 1H), 3.04 (s, 1H). ^13^C NMR (101 MHz, Chloroform-*d*) *δ* 158.0, 157.0, 154.5, 154.5, 139.0, 134.9, 134.6, 127.5, 121.4, 116.3, 116.3, 116.2, 116.1, 114.7, 114.6, 114.5, 107.4, 79.5, 72.4. ^19^F NMR (565 MHz, Chloroform-d) δ −61.41, −125.96. HRMS(ESI): calcd for C_20_H_14_BrF_5_N_2_O_2_, [M + H]^+^ 489.0237; found, 489.0235. HPLC purity: 99.72%.

#### 2-(3,4-Difluorophenoxy)-1-(4-((5-(trifluoromethyl)pyridin-2-yl)amino)phenyl)ethan-1-ol (2)

The titled compound was prepared from **E_2_** (50 mg, 0.12 mmol) and purified by column chromatography on silica gel with the elution fluid of Hexane/EA (2:1–1:1). Pale yellow solid, 39 mg, 78% yield, mp 107–108 °C. ^1^H NMR (400 MHz, Chloroform-*d*) *δ* 8.47 (s, 1H), 7.70 (dd, *J* = 8.8, 2.4 Hz, 1H), 7.50–7.39 (m, 4H), 7.09 (q, *J* = 9.3 Hz, 1H), 6.97 (s, 1H), 6.87 (d, *J* = 8.8 Hz, 1H), 6.77 (m, *J* = 11.8, 6.5, 3.0 Hz, 1H), 6.69–6.61 (m, 1H), 5.13 (dd, *J* = 8.4, 3.4 Hz, 1H), 4.09–3.98 (m, 2H), 2.88 (s, 1H). ^13^C NMR (101 MHz, Chloroform-*d*) *δ* 158.0, 154.7, 154.6, 151.8, 151.6, 149.2, 146.6, 146.5, 144.2, 144.1, 139.1, 135.1, 134.9, 134.9, 127.4, 125.5, 122.8, 121.4, 117.4, 117.2, 109.9, 109.9, 109.9, 109.9, 104.5, 104.3, 73.9, 72.1. HRMS(ESI): calcd for C_20_H_15_F_5_N_2_O_2_, [M + H]^+^ 411.1132; found, 411.1131. HPLC purity: 99.85%.

#### 2-(4-(Trifluoromethyl)phenoxy)-1-(4-((5-(trifluoromethyl)pyridin-2-yl)amino)phenyl)ethan-1-ol (3)

The titled compound was prepared from **E_3_** (50 mg, 0.11 mmol) and purified by column chromatography on silica gel with the elution fluid of Hexane/EA (2:1–1:1). Pale yellow solid, 37.6 mg, 75% yield, mp 129–131 °C. ^1^H NMR (400 MHz, Chloroform-*d*) *δ* 8.45 (s, 1H), 7.69 (dd, *J* = 8.8, 2.4 Hz, 1H), 7.57 (d, *J* = 8.5 Hz, 2H), 7.48 (d, *J* = 8.2 Hz, 2H), 7.40 (d, *J* = 8.5 Hz, 2H), 7.00 (d, *J* = 8.5 Hz, 2H), 6.87 (d, *J* = 8.8 Hz, 1H), 5.17 (dd, *J* = 8.2, 3.6 Hz, 1H), 4.19–4.08 (m, 2H), 3.22 (s, 1H). ^13^C NMR (101 MHz, Chloroform-*d*) *δ* 160.8, 158.1, 139.2, 135.3, 135.0, 135.0, 134.9, 134.9, 128.2, 127.5, 127.1, 127.0, 127.0, 127.0, 125.7, 125.5, 123.7, 123.4, 123.0, 122.8, 121.5, 117.9, 117.5, 114.6, 107.5, 73.3, 72.0. HRMS(ESI): calcd for C_21_H_16_F_6_N_2_O_2_, [M + H]^+^ 443.1194; found, 443.1187. HPLC purity: 99.59%.

#### 2-(4-Bromo-2,6-difluorophenoxy)-1-(4-((4-(trifluoromethyl)phenyl)amino)phenyl)ethan-1-ol (4)

The titled compound was prepared from **E_4_** (50 mg, 0.1 mmol) and purified by column chromatography on silica gel with the elution fluid of Hexane/EA (5:1–2:1). Dark yellow oil, 46.8 mg, 93% yield. ^1^H NMR (400 MHz, Chloroform-*d*) *δ* 7.50 (d, *J* = 8.4 Hz, 2H), 7.42–7.35 (m, 2H), 7.20–7.10 (m, 4H), 7.06 (d, *J* = 8.4 Hz, 2H), 5.99 (s, 1H), 5.10–5.03 (m, 1H), 4.30 (dd, *J* = 10.2, 3.1 Hz, 1H), 4.13 (t, *J* = 9.6 Hz, 1H), 2.95 (s, 1H). ^13^C NMR (101 MHz, Chloroform-*d*) *δ* 157.1, 157.0, 154.6, 154.5, 146.4, 141.3, 135.0, 134.9, 134.8, 133.0, 127.6, 126.8, 126.7, 126.7, 126.7, 125.9, 123.2, 122.1, 121.8, 121.5, 119.7, 116.3, 116.3, 116.2, 116.1, 115.6, 114.7, 114.6, 114.5, 79.6, 72.4. ^19^F NMR (565 MHz, Chloroform-d) δ −61.49, −125.93. HRMS(ESI): calcd for C_21_H_15_BrF_5_NO_2_, [M + H]^+^ 488.0285; found, 488.0273. HPLC purity: 99.19%.

#### 2-(3,4-Difluorophenoxy)-1-(4-((4-(trifluoromethyl)phenyl)amino)phenyl)ethan-1-ol (5)

The titled compound was prepared from **E_5_** (50 mg, 0.12 mmol) and purified by column chromatography on silica gel with the elution fluid of Hexane/EA (5:1–2:1). Dark yellow oil, 45.4 mg, 90% yield. ^1^H NMR (600 MHz, Chloroform-*d*) *δ* 7.51 (d, *J* = 8.4 Hz, 2H), 7.44–7.40 (m, 2H), 7.21–7.17 (m, 2H), 7.12–7.07 (m, 3H), 6.78 (m, *J* = 11.8, 6.5, 3.0 Hz, 1H), 6.65 (m, *J* = 8.6, 3.2, 1.7 Hz, 1H), 6.04–5.99 (m, 1H), 5.11 (dd, *J* = 8.7, 3.2 Hz, 1H), 4.08–3.98 (m, 2H), 2.76–2.69 (m, 1H). ^13^C NMR (151 MHz, Chloroform-*d*) *δ* 154.7, 154.7, 154.7, 154.6, 151.3, 151.3, 149.7, 149.6, 146.4, 146.2, 146.1, 144.6, 144.5, 141.4, 133.5, 127.6, 126.8, 126.8, 126.7, 126.7, 125.4, 123.6, 122.1, 121.9, 119.7, 117.4, 117.3, 115.6, 110.0, 109.9, 109.9, 109.9, 104.5, 104.3, 74.0, 72.1. ^19^F NMR (565 MHz, CDCl3) δ −61.49, −135.10 (d, J = 21.5 Hz), −147.44 (d, J = 21.5 Hz). HRMS(ESI): calcd for C_21_H_16_F_5_NO_2_, [M + H]^+^ 410.1179; found, 410.1178. HPLC purity: 98.75%.

#### 2-(4-(Trifluoromethyl)phenoxy)-1-(4-((4-(trifluoromethyl)phenyl)amino)phenyl)ethan-1-ol (6)

The titled compound was prepared from **E_6_** (50 mg, 0.11 mmol) and purified by column chromatography on silica gel with the elution fluid of Hexane/EA (5:1–2:1). Dark yellow oil, 46.6 mg, 93% yield. ^1^H NMR (400 MHz, Chloroform-*d*) *δ* 7.58 (d, *J* = 8.5 Hz, 2H), 7.51 (d, *J* = 8.3 Hz, 2H), 7.44 (d, *J* = 8.4 Hz, 2H), 7.22–7.16 (m, 2H), 7.09 (d, *J* = 8.4 Hz, 2H), 7.02 (d, *J* = 8.5 Hz, 2H), 6.06 (s, 1H), 5.15 (dd, *J* = 8.5, 3.4 Hz, 1H), 4.19–4.08 (m, 2H), 2.88 (d, *J* = 7.0 Hz, 1H). ^13^C NMR (101 MHz, Chloroform-*d*) *δ* 160.8, 146.4, 141.5, 133.5, 127.6, 127.1, 127.0, 127.0, 127.0, 126.8, 126.8, 126.7, 126.7, 125.9, 125.7, 123.7, 123.3, 123.2, 123.0, 122.2, 121.9, 121.5, 119.7, 115.7, 114.6, 73.3, 72.1. ^19^F NMR (565 MHz, Chloroform-d) δ −61.49, −61.54. HRMS(ESI): calcd for C_22_H_17_F_6_NO_2_, [M - H]^−^ 440.1085; found, 440.1080. HPLC purity: 98.04%.

#### 2-(3-(Trifluoromethyl)phenoxy)-1-(4-((4-(trifluoromethyl)phenyl)amino)phenyl)ethan-1-ol (7)

The titled compound was prepared from **E_7_** (50 mg, 0.11 mmol) and purified by column chromatography on silica gel with the elution fluid of Hexane/EA (5:1–2:1). Dark yellow oil, 46.3 mg, 92% yield. ^1^H NMR (400 MHz, Chloroform-*d*) *δ* 7.51 (d, *J* = 8.4 Hz, 2H), 7.44 (d, *J* = 8.2 Hz, 3H), 7.28 (d, *J* = 2.0 Hz, 1H), 7.19 (dd, *J* = 9.0, 2.4 Hz, 3H), 7.13 (dd, *J* = 8.3, 2.5 Hz, 1H), 7.09 (d, *J* = 8.4 Hz, 2H), 6.03 (s, 1H), 5.14 (dd, *J* = 8.6, 3.3 Hz, 1H), 4.18–4.07 (m, 2H), 2.81 (s, 1H). ^13^C NMR (101 MHz, Chloroform-*d*) *δ* 158.5, 146.4, 141.4, 133.6, 132.4, 132.1, 131.8, 131.5, 130.1, 127.6, 126.8, 126.8, 126.7, 126.7, 125.9, 125.2, 123.2, 122.5, 122.2, 121.8, 121.5, 119.7, 118.2, 118.1, 118.0, 115.6, 111.4, 111.3, 73.4, 72.2. ^19^F NMR (565 MHz, Chloroform-d) δ −61.48, −62.70. HRMS(ESI): calcd for C_22_H_17_F_6_NO_2_, [M - H]^−^ 440.1085; found, 440.1093. HPLC purity: 99.44%.

#### 2-(2-(Trifluoromethyl)phenoxy)-1-(4-((4-(trifluoromethyl)phenyl)amino)phenyl)ethan-1-ol (8)

The titled compound was prepared from **E_8_** (50 mg, 0.11 mmol) and purified by column chromatography on silica gel with the elution fluid of Hexane/EA (5:1–2:1). Pale yellow solid, 46.4 mg, 92% yield, mp 152–154 °C. ^1^H NMR (600 MHz, Chloroform-*d*) *δ* 7.63 (dd, *J* = 7.8, 1.6 Hz, 1H), 7.50 (d, *J* = 8.3 Hz, 3H), 7.46–7.43 (m, 2H), 7.20–7.17 (m, 2H), 7.08 (dd, *J* = 8.0, 4.9 Hz, 3H), 7.00 (d, *J* = 8.3 Hz, 1H), 6.01 (s, 1H), 5.17 (dd, *J* = 8.6, 3.3 Hz, 1H), 4.25 (dd, *J* = 9.1, 3.3 Hz, 1H), 4.09 (t, *J* = 8.9 Hz, 1H), 2.93 (s, 1H). ^13^C NMR (151 MHz, Chloroform-*d*) *δ* 156.2, 146.5, 141.3, 133.4, 127.7, 127.3, 127.2, 127.2, 127.2, 126.8, 126.7, 126.7, 126.7, 125.5, 124.7, 123.7, 122.9, 122.0, 121.8, 120.8, 119.8, 119.0, 118.8, 115.6, 113.1, 74.0, 71.9. ^19^F NMR (565 MHz, Chloroform-d) δ −61.48, −62.07. HRMS(ESI): calcd for C_22_H_17_F_6_NO_2_, [M - H]^−^ 440.1085; found, 440.1092. HPLC purity: 99.58%.

#### 2-(4-Nitrophenoxy)-1-(4-((4-(trifluoromethyl)phenyl)amino)phenyl)ethan-1-ol (9)

The titled compound was prepared from **E_9_** (50 mg, 0.12 mmol) and purified by column chromatography on silica gel with the elution fluid of Hexane/EA (5:1–2:1). Yellow solid, 37.4 mg, 75% yield, mp 151–153 °C. ^1^H NMR (600 MHz, Chloroform-*d*) *δ* 8.24 (d, *J* = 9.0 Hz, 2H), 7.51 (d, *J* = 8.4 Hz, 2H), 7.44 (d, *J* = 8.1 Hz, 2H), 7.20 (d, *J* = 8.1 Hz, 2H), 7.10 (d, *J* = 8.4 Hz, 2H), 7.02 (d, *J* = 8.9 Hz, 2H), 6.03 (s, 1H), 5.17 (dd, *J* = 8.4, 3.4 Hz, 1H), 4.22–4.13 (m, 2H), 2.68 (s, 1H). ^13^C NMR (151 MHz, Chloroform-*d*) *δ* 163.4, 146.2, 141.9, 141.6, 133.2, 127.6, 127.2, 126.8, 126.8, 126.8, 126.7, 126.0, 125.4, 123.6, 122.5, 122.3, 122.1, 119.6, 115.8, 114.6, 73.7, 72.1. HRMS(ESI): calcd for C_21_H_17_F_3_N_2_O_4_, [M - H]^−^ 417.1062; found, 417.1067. HPLC purity: 100%.

#### 4-(2-Hydroxy-2-(4-((4-(trifluoromethyl)phenyl)amino)phenyl)ethoxy)benzonitrile (10)

The titled compound was prepared from **E_10_** (50 mg, 0.13 mmol) and purified by column chromatography on silica gel with the elution fluid of Hexane/EA (2:1–1:1). White solid, 42.1 mg, 84% yield, mp 151–153 °C. ^1^H NMR (600 MHz, DMSO-*d*_6_) *δ* 8.72 (s, 1H), 7.77–7.73 (m, 2H), 7.51 (d, *J* = 8.6 Hz, 2H), 7.42–7.38 (m, 2H), 7.18–7.15 (m, 2H), 7.14–7.10 (m, 4H), 5.66 (d, *J* = 4.6 Hz, 1H), 4.91 (dt, *J* = 7.3, 4.6 Hz, 1H), 4.15–4.09 (m, 2H). ^13^C NMR (151 MHz, DMSO-*d*_6_) *δ* 162.5, 148.1, 141.1, 135.8, 134.6, 128.0, 127.0, 127.0, 127.0, 126.9, 126.3, 124.5, 119.6, 119.3, 119.1, 118.9, 118.7, 118.4, 116.2, 114.9, 103.2, 73.7, 70.8. ^19^F NMR (565 MHz, Chloroform-d) δ −61.51. HRMS(ESI): calcd for C_22_H_17_F_3_N_2_O_2_, [M - H]^−^ 397.1164; found, 397.1173. HPLC purity: 97.74%.

#### 2-(4-(Methylthio)phenoxy)-1-(4-((4-(trifluoromethyl)phenyl)amino)phenyl)ethan-1-ol (11)

The titled compound was prepared from **E_11_** (50 mg, 0.12 mmol) and purified by column chromatography on silica gel with the elution fluid of Hexane/EA (5:1–2:1). Pale yellow solid, 45.3 mg, 90% yield, mp 43–45 °C. ^1^H NMR (600 MHz, Chloroform-*d*) *δ* 7.50 (d, *J* = 8.4 Hz, 2H), 7.44–7.40 (m, 2H), 7.30–7.27 (m, 2H), 7.19–7.16 (m, 2H), 7.07 (d, *J* = 8.4 Hz, 2H), 6.92–6.89 (m, 2H), 6.06 (s, 1H), 5.11 (dd, *J* = 8.8, 3.1 Hz, 1H), 4.10 (dd, *J* = 9.5, 3.3 Hz, 1H), 4.03 (t, *J* = 9.1 Hz, 1H), 2.95 (s, 1H), 2.47 (s, 3H). ^13^C NMR (151 MHz, Chloroform-*d*) *δ* 156.9, 146.5, 141.3, 133.8, 129.9, 129.7, 127.6, 126.8, 126.8, 126.7, 126.7, 125.5, 123.7, 122.0, 121.7, 119.7, 115.6, 115.4, 73.4, 72.2, 17.8. ^19^F NMR (565 MHz, Chloroform-d) δ −61.49. HRMS(ESI): calcd for C_22_H_20_F_3_NO_2_S, [M - H]^−^ 418.1089; found, 418.1097. HPLC purity: 96.22%.

#### 1-(4-((4-(Trifluoromethyl)phenyl)amino)phenyl)-2-((5-(trifluoromethyl)pyridin-2-yl)oxy)ethan-1-ol (12)

The titled compound was prepared from **E_12_** (50 mg, 0.11 mmol) and purified by column chromatography on silica gel with the elution fluid of Hexane/EA (2:1–1:1). Pale yellow solid, 46.4 mg, 92% yield, mp 148–149 °C. ^1^H NMR (400 MHz, DMSO-*d*_6_) *δ* 8.72 (s, 1H), 8.15 (d, *J* = 2.7 Hz, 1H), 7.67 (dd, *J* = 9.6, 2.8 Hz, 1H), 7.50 (d, *J* = 8.4 Hz, 2H), 7.33 (d, *J* = 8.5 Hz, 2H), 7.15 (dd, *J* = 18.0, 8.3 Hz, 4H), 6.57 (d, *J* = 9.5 Hz, 1H), 5.68 (d, *J* = 4.9 Hz, 1H), 4.80 (dt, *J* = 8.9, 4.2 Hz, 1H), 4.24 (dd, *J* = 12.9, 3.6 Hz, 1H), 3.90 (dd, *J* = 12.9, 9.2 Hz, 1H). ^13^C NMR (101 MHz, DMSO-*d*_6_) *δ* 161.6, 148.0, 141.3, 136.0, 135.7, 127.4, 126.9, 126.9, 126.7, 125.7, 124.0, 123.0, 120.4, 119.4, 119.1, 118.7, 115.0, 107.0, 106.7, 79.7, 79.4, 79.1, 69.7, 56.7. ^19^F NMR (565 MHz, Chloroform-d) δ −61.52, −62.32. HRMS(ESI): calcd for C_21_H_16_F_6_N_2_O_2_, [M - H]^−^ 441.1038; found, 441.1047. HPLC purity: 99.56%.

#### 1-(4-((4-(Trifluoromethyl)phenyl)amino)phenyl)-2-((4-(trifluoromethyl)pyridin-2-yl)oxy)ethan-1-ol (13)

The titled compound was prepared from **E_13_** (50 mg, 0.11 mmol) and purified by column chromatography on silica gel with the elution fluid of Hexane/EA (2:1–1:1). Pale yellow solid, 46 mg, 92% yield, mp 157–159 °C. ^1^H NMR (400 MHz, Chloroform-*d*) *δ* 7.50 (d, *J* = 8.4 Hz, 2H), 7.39 (dd, *J* = 10.2, 7.7 Hz, 3H), 7.17 (s, 2H), 7.07 (d, *J* = 8.4 Hz, 2H), 6.88 (s, 1H), 6.31 (dd, *J* = 7.1, 1.9 Hz, 1H), 6.06 (s, 1H), 5.12 (d, *J* = 8.3 Hz, 1H), 4.49 (dd, *J* = 13.4, 3.0 Hz, 1H), 3.88 (dd, *J* = 13.4, 8.4 Hz, 1H), 3.40 (d, *J* = 3.6 Hz, 1H). ^13^C NMR (101 MHz, Chloroform-*d*) *δ* 162.5, 146.3, 141.9, 141.6, 141.4, 140.6, 134.9, 127.0, 126.8, 126.7, 123.4, 123.2, 122.2, 121.9, 119.7, 118.1, 115.6, 101.1, 101.1, 72.1, 58.0. ^19^F NMR (565 MHz, Chloroform-d) δ −61.53, −66.63. HRMS(ESI): calcd for C_21_H_16_F_6_N_2_O_2_, [M - H]^−^ 441.1038; found, 441.1050. HPLC purity: 99.45%.

#### 1-(4-((4-(Trifluoromethyl)phenyl)amino)phenyl)-2-((5-(trifluoromethyl)pyridin-3-yl)oxy)ethan-1-ol (14)

The titled compound was prepared from **E_14_** (50 mg, 0.11 mmol) and purified by column chromatography on silica gel with the elution fluid of Hexane/EA (2:1–1:1). Pale yellow solid, 46.7 mg, 93% yield, mp 147–148 °C. ^1^H NMR (600 MHz, Chloroform-*d*) *δ* 8.51 (q, *J* = 3.0 Hz, 2H), 7.50 (d, *J* = 8.4 Hz, 2H), 7.45–7.41 (m, 3H), 7.19 (d, *J* = 8.1 Hz, 2H), 7.09 (d, *J* = 8.3 Hz, 2H), 6.11 (q, *J* = 4.3, 3.8 Hz, 1H), 5.16 (dd, *J* = 7.6, 4.2 Hz, 1H), 4.21–4.16 (m, 2H), 3.20–3.02 (m, 1H). ^13^C NMR (151 MHz, Chloroform-*d*) *δ* 154.5, 146.2, 141.7, 139.0, 138.9, 138.9, 138.9, 133.3, 127.6, 127.3, 127.1, 126.8, 126.8, 126.7, 126.7, 125.4, 124.1, 123.6, 122.3, 122.2, 122.0, 121.8, 119.6, 117.9, 117.9, 117.8, 117.8, 115.8, 73.7, 72.1. ^19^F NMR (565 MHz, Chloroform-d) δ −61.54, −62.34. HRMS(ESI): calcd for C_21_H_16_F_6_N_2_O_2_, [M + H]^+^ 443.1194; found, 443.1191. HPLC purity: 99.11%.

#### 2-(4-(Trifluoromethyl)phenoxy)-1-(4-((3-(trifluoromethyl)phenyl)amino)phenyl)ethan-1-ol (15)

The titled compound was prepared from **E_15_** (50 mg, 0.11 mmol) and purified by column chromatography on silica gel with the elution fluid of Hexane/EA (5:1–2:1). Dark yellow oil, 46.1 mg, 92% yield. ^1^H NMR (400 MHz, Chloroform-*d*) *δ* 7.58 (d, *J* = 8.4 Hz, 2H), 7.39 (dd, *J* = 18.9, 8.1 Hz, 3H), 7.30 (d, *J* = 10.9 Hz, 1H), 7.24–7.12 (m, 4H), 7.02 (d, *J* = 8.4 Hz, 2H), 5.99 (s, 1H), 5.13 (dd, *J* = 8.4, 3.5 Hz, 1H), 4.17–4.10 (m, 2H), 2.91 (s, 1H). ^13^C NMR (101 MHz, Chloroform-*d*) *δ* 171.4, 160.9, 143.7, 142.1, 132.9, 132.3, 131.9, 131.6, 131.3, 129.9, 128.4, 127.7, 127.1, 127.0, 127.0, 126.9, 125.7, 125.4, 123.9, 123.6, 123.3, 123.0, 122.7, 120.3, 120.1, 118.7, 117.3, 117.3, 117.3, 117.2, 114.6, 113.5, 113.5, 113.5, 113.4, 73.3, 72.2. ^19^F NMR (565 MHz, Chloroform-d) δ −61.49, −62.80. HRMS(ESI): calcd for C_21_H_16_F_6_N_2_O_2_, [M - H]^−^ 440.1085; found, 440.1095. HPLC purity: 96.82%.

#### 2-(4-(Trifluoromethyl)phenoxy)-1-(4-((2-(trifluoromethyl)phenyl)amino)phenyl)ethan-1-ol (16)

The titled compound was prepared from **E_16_** (50 mg, 0.11 mmol) and purified by column chromatography on silica gel with the elution fluid of Hexane/EA (5:1–2:1). Pale yellow solid, 45.4 mg, 91% yield, mp 88–89 °C. ^1^H NMR (400 MHz, Chloroform-*d*) *δ* 7.60 (dd, *J* = 10.4, 8.1 Hz, 3H), 7.45–7.36 (m, 4H), 7.16 (d, *J* = 8.5 Hz, 2H), 7.04–6.98 (m, 3H), 6.12 (s, 1H), 5.14 (dd, *J* = 8.5, 3.4 Hz, 1H), 4.18–4.07 (m, 2H), 2.75 (s, 1H). ^13^C NMR (101 MHz, Chloroform-*d*) *δ* 160.8, 142.0, 141.7, 133.3, 132.7, 127.6, 127.1, 127.0, 127.0, 127.0, 126.0, 125.7, 123.6, 123.3, 123.0, 120.3, 119.8, 118.3, 118.2, 117.9, 114.6, 73.3, 72.1. HRMS(ESI): calcd for C_21_H_16_F_6_N_2_O_2_, [M - H]^−^ 440.1085; found, 440.1097. HPLC purity: 98.82%.

#### 1-(4-((4-Methoxyphenyl)amino)phenyl)-2-(4-(trifluoromethyl)phenoxy)ethan-1-ol (17)

The titled compound was prepared from **E_17_** (50 mg, 0.12 mmol) and purified by column chromatography on silica gel with the elution fluid of Hexane/EA (5:1–2:1). Pale yellow solid, 44.1 mg, 88% yield, mp 121–122 °C. ^1^H NMR (400 MHz, Chloroform-*d*) *δ* 7.57 (d, *J* = 8.6 Hz, 2H), 7.31 (d, *J* = 8.1 Hz, 2H), 7.15–6.98 (m, 4H), 6.92 (dd, *J* = 13.0, 8.9 Hz, 4H), 5.08 (d, *J* = 6.4 Hz, 1H), 4.14–4.07 (m, 2H), 3.83 (s, 3H). ^13^C NMR (101 MHz, Chloroform-*d*) *δ* 160.9, 155.6, 145.6, 135.3, 130.1, 127.5, 127.0, 127.0, 127.0, 126.9, 125.7, 123.5, 123.2, 123.0, 122.9, 122.6, 115.4, 114.7, 114.6, 73.4, 72.2, 55.6. HRMS(ESI): calcd for C_22_H_20_F_3_NO_3_, [M + H]^+^ 404.1474; found, 404.1466. HPLC purity: 98.89%.

#### 1-(4-((4-(Methylthio)phenyl)amino)phenyl)-2-(4-(trifluoromethyl)phenoxy)ethan-1-ol (18)

The titled compound was prepared from **E_18_** (50 mg, 0.12 mmol) and purified by column chromatography on silica gel with the elution fluid of Hexane/EA (5:1–2:1). Pale yellow solid, 44.8 mg, 89% yield, mp 95–97 °C. ^1^H NMR (600 MHz, Chloroform-*d*) *δ* 7.58 (d, *J* = 8.4 Hz, 2H), 7.36 (d, *J* = 8.2 Hz, 2H), 7.28–7.25 (m, 2H), 7.09–6.99 (m, 6H), 5.77 (s, 1H), 5.11 (dd, *J* = 8.6, 3.3 Hz, 1H), 4.16–4.07 (m, 2H), 2.69 (s, 1H), 2.49 (s, 3H). ^13^C NMR (151 MHz, Chloroform-*d*) *δ* 160.9, 143.4, 140.8, 131.6, 129.7, 129.7, 127.5, 127.0, 127.0, 127.0, 127.0, 126.4, 125.2, 123.5, 123.4, 123.3, 120.1, 119.0, 117.4, 114.6, 73.4, 72.2, 17.7. ^19^F NMR (565 MHz, Chloroform-d) δ −61.52. HRMS(ESI): calcd for C_22_H_20_F_3_NO_2_S, [M - H]^−^ 418.1089; found, 418.1094. HPLC purity: 95.01%.

#### 2-((4-(1-Hydroxy-2-(4-(trifluoromethyl)phenoxy)ethyl)phenyl)amino)benzonitrile (19)

The titled compound was prepared from **E_19_** (50 mg, 0.12 mmol) and purified by column chromatography on silica gel with the elution fluid of Hexane/EA (2:1–1:1). White solid, 47.5 mg, 95% yield, mp 158–160 °C. ^1^H NMR (600 MHz, Chloroform-*d*) *δ* 9.61 (s, 1H), 7.58 (d, *J* = 8.5 Hz, 2H), 7.50 (dd, *J* = 7.9, 1.4 Hz, 1H), 7.42 (d, *J* = 8.2 Hz, 2H), 7.37 (d, *J* = 8.3 Hz, 1H), 7.33 (dd, *J* = 8.3, 6.7 Hz, 1H), 7.27 (d, *J* = 8.2 Hz, 2H), 7.02 (d, *J* = 8.5 Hz, 2H), 6.81 (t, *J* = 7.5 Hz, 1H), 5.14 (d, *J* = 8.6 Hz, 1H), 4.17–4.09 (m, 2H), 2.74 (d, *J* = 2.5 Hz, 1H). ^13^C NMR (151 MHz, Chloroform-*d*) *δ* 171.6, 160.9, 146.1, 141.5, 133.4, 133.0, 129.9, 129.9, 128.3, 127.4, 127.0, 127.0, 127.0, 127.0, 126.5, 125.2, 123.7, 123.5, 123.4, 123.3, 121.2, 117.9, 116.1, 115.4, 114.6, 73.4, 72.2. HRMS(ESI): calcd for C_22_H_17_F_3_N_2_O_2_, [M + H]^+^ 399.1320; found, 399.1320. HPLC purity: 96.16%.

#### 2-((4-(Trifluoromethyl)benzyl)oxy)-1-(4-((4-(trifluoromethyl)phenyl)amino)phenyl)ethan-1-ol (23)

The titled compound was prepared from **E_20_** (50 mg, 0.11 mmol) and purified by column chromatography on silica gel with the elution fluid of Hexane/EA (5:1–2:1). Pale yellow solid, 43.7 mg, 87% yield, mp 89–91 °C. ^1^H NMR (600 MHz, Chloroform-*d*) *δ* 7.64 (d, *J* = 8.0 Hz, 2H), 7.51–7.47 (m, 4H), 7.38–7.35 (m, 2H), 7.17–7.13 (m, 2H), 7.06 (d, *J* = 8.4 Hz, 2H), 5.96 (s, 1H), 4.95 (dd, *J* = 8.8, 3.2 Hz, 1H), 4.73–4.66 (m, 2H), 3.68 (dd, *J* = 9.7, 3.3 Hz, 1H), 3.58 (t, *J* = 9.3 Hz, 1H), 2.77 (s, 1H). ^13^C NMR (151 MHz, Chloroform-*d*) *δ* 146.5, 141.8, 141.0, 134.4, 129.9, 127.7, 127.5, 126.7, 126.7, 125.5, 119.8, 115.4, 76.0, 72.6. HRMS(ESI): calcd for C_23_H_19_F_6_NO_2_, [M - H]^−^ 454.1242; found, 454.1248. HPLC purity: 99.03%.

#### 1-(4-((4-(Trifluoromethyl)phenyl)amino)phenyl)-2-((4-(trifluoromethyl)phenyl)thio)ethan-1-ol (24)

The titled compound was prepared from **E_21_** (50 mg, 0.11 mmol) and purified by column chromatography on silica gel with the elution fluid of Hexane/EA (5:1–2:1). Dark yellow oil, 46.7 mg, 93% yield. ^1^H NMR (400 MHz, Chloroform-*d*) *δ* 7.56 (d, *J* = 8.1 Hz, 2H), 7.49 (dd, *J* = 15.5, 8.3 Hz, 4H), 7.35 (d, *J* = 8.2 Hz, 2H), 7.15 (d, *J* = 8.2 Hz, 2H), 7.07 (d, *J* = 8.3 Hz, 2H), 6.00 (s, 1H), 4.82 (dd, *J* = 8.8, 4.0 Hz, 1H), 3.40 (dd, *J* = 13.7, 4.1 Hz, 1H), 3.27 (dd, *J* = 13.7, 8.7 Hz, 1H), 2.71 (s, 1H). ^13^C NMR (101 MHz, Chloroform-*d*) *δ* 146.3, 141.3, 140.8, 136.1, 128.6, 128.4, 128.3, 128.1, 128.0, 127.7, 127.2, 126.8, 126.8, 126.7, 126.7, 125.9, 125.9, 125.8, 125.8, 125.4, 123.2, 122.7, 122.5, 122.2, 121.9, 121.5, 119.7, 115.6, 71.8, 42.3. ^19^F NMR (565 MHz, Chloroform-d) δ −61.47, −62.45. HRMS(ESI): calcd for C_22_H_17_F_6_NOS, [M - H]^−^ 456.0857; found, 456.0862. HPLC purity: 98.74%.

#### 2-(4-(Trifluoromethyl)phenoxy)-1-(3-((2-(trifluoromethyl)phenyl)amino)phenyl)ethan-1-ol (31)

The titled compound was prepared from **E_27_** (50 mg, 0.11 mmol) and purified by column chromatography on silica gel with the elution fluid of Hexane/EA (5:1–2:1). Dark yellow oil, 46.4 mg, 92% yield. ^1^H NMR (400 MHz, Chloroform-*d*) *δ* 7.59 (dd, *J* = 11.5, 8.2 Hz, 3H), 7.44–7.32 (m, 3H), 7.24 (t, *J* = 1.9 Hz, 1H), 7.14–7.09 (m, 2H), 7.01 (d, *J* = 8.4 Hz, 3H), 6.13 (s, 1H), 5.14 (dd, *J* = 8.6, 3.2 Hz, 1H), 4.20–4.07 (m, 2H), 2.77 (s, 1H). ^13^C NMR (101 MHz, Chloroform-*d*) *δ* 160.8, 142.3, 141.6, 141.0, 132.7, 129.8, 127.1, 127.0, 127.0, 127.0, 126.0, 125.7, 123.7, 123.4, 123.3, 123.0, 120.3, 120.3, 119.5, 118.3, 118.2, 117.9, 117.5, 114.6, 73.4, 72.3. HRMS(ESI): calcd for C_22_H_17_F_6_NO_2_, [M - H]^−^ 440.1085; found, 440.1093. HPLC purity: 98.59%.

General procedure for the synthesis of compound **C_9_** and **C_10_**. To a solution of phenol or benzenethiol (1 equiv) in NMP, 1–(4-fluorophenyl)ethan-1-one (1 equiv) and Cs_2_CO_3_ (2 equiv) was added. The mixture was stirred at 120 °C under nitrogen for overnight and monitored to react completely. After cooling to room temperature and filtration with diatomite, water was added and the filtrate was extracted with ethyl acetate for three times and washed with saturated brine. The combined organic layer was dried over anhydrous Na_2_SO_4_ and purified by column chromatography on silica gel to provide the titled compounds.

#### 1-(4-(4-(Trifluoromethyl)phenoxy)phenyl)ethan-1-one (C9)

The titled compound was prepared from 4-(trifluoromethyl)phenol (1 g, 6.17 mmol) and 1–(4-fluorophenyl)ethan-1-one (852.1 mg, 6.17 mmol), purified by column chromatography on silica gel with the elution fluid of Hexane/EA (200:1–100:1). Pale yellow solid, 1.54 g, 89% yield. ^1^H NMR (400 MHz, Chloroform-*d*) *δ* 8.03–7.99 (m, 2H), 7.66 (d, *J* = 8.5 Hz, 2H), 7.15 (d, *J* = 8.3 Hz, 2H), 7.11–7.07 (m, 2H), 2.62 (s, 3H).

#### 1-(4-((4-(Trifluoromethyl)phenyl)thio)phenyl)ethan-1-one (C10)

The titled compound was prepared from 4-(trifluoromethyl)benzenethiol (1 g, 5.61 mmol) and 1–(4-fluorophenyl)ethan-1-one (775.3 mg, 5.61 mmol), purified by column chromatography on silica gel with the elution fluid of Hexane/EA (200:1–100:1). Yellow solid, 1.03 g, 62% yield. ^1^H NMR (400 MHz, Chloroform-*d*) *δ* 7.94–7.90 (m, 2H), 7.61 (d, *J* = 8.5 Hz, 2H), 7.49 (d, *J* = 8.2 Hz, 2H), 7.42–7.38 (m, 2H), 2.61 (s, 3H).

General procedure for the synthesis of compound **D_9_** and **D_10_**. To a solution of compound **C_9_** or **C_10_** (1 equiv) and TsOH (1 equiv) in acetonitrile, NBS (1.1 equiv) was added in ice bath. After warming to room temperature, the mixture was stirred at 90 °C for 8 h and monitored to react completely. After cooling to room temperature, the solution was concentrated and purified by column chromatography on silica gel to provide the titled compounds.

#### 2-Bromo-1-(4-(4-(trifluoromethyl)phenoxy)phenyl)ethan-1-one (D9)

The titled compound was prepared from **C_9_**(100 mg, 0.28 mmol), purified by column chromatography on silica gel with the elution fluid of Hexane/EA (200:1–150:1). Yellow solid, 437.6 mg, 68% yield. ^1^H NMR (400 MHz, Chloroform-*d*) *δ* 8.08–8.02 (m, 2H), 7.69 (d, *J* = 8.5 Hz, 2H), 7.18 (d, *J* = 8.4 Hz, 2H), 7.12–7.08 (m, 2H), 4.44 (s, 2H).

#### 2-Bromo-1-(4-((4-(trifluoromethyl)phenyl)thio)phenyl)ethan-1-one (D10)

The titled compound was prepared from **C_10_**(500 mg, 1.69 mmol), purified by column chromatography on silica gel with the elution fluid of Hexane/EA (200:1–150:1). Yellow solid, 410.3 mg, 65% yield. ^1^H NMR (600 MHz, Chloroform-*d*) *δ* 7.48 (d, *J* = 8.2 Hz, 2H), 7.43–7.40 (m, 2H), 7.32 (d, *J* = 8.1 Hz, 2H), 7.28 (d, *J* = 2.0 Hz, 2H), 4.14 (s, 2H).

General procedure for the synthesis of compound **E_21_** and **E_22_**. To a solution of 4-(trifluoromethyl)phenol (1.5 equiv) in acetone, K_2_CO_3_ (2 equiv) was added. The mixture was stirred at room temperature for 30 min. With the following adding of selected compound **D_9_** or **D_10_** (1 equiv), it was stirred at reflux for overnight and monitored to react completely. After cooling to room temperature and filtration, the filtrate was concentrated and purified by column chromatography on silica gel to provide the titled compounds.

#### 2-(4-(Trifluoromethyl)phenoxy)-1-(4-(4-(trifluoromethyl)phenoxy)phenyl)ethan-1-one (E21)

The titled compound was prepared from **D_9_**(100 mg, 0.28 mmol) and 4-(trifluoromethyl)phenol (67.7 mg, 0.42 mmol), purified by column chromatography on silica gel with the elution fluid of Hexane/EA (50:1–20:1). Pale yellow solid, 106.3 mg, 87% yield. ^1^H NMR (400 MHz, Chloroform-*d*) *δ* 7.99 (d, *J* = 8.6 Hz, 2H), 7.55 (d, *J* = 8.5 Hz, 2H), 7.42 (t, *J* = 7.8 Hz, 2H), 7.11–6.97 (m, 6H), 5.29 (s, 2H).

#### 2-(4-(Trifluoromethyl)phenoxy)-1-(4-((4-(trifluoromethyl)phenyl)thio)phenyl)ethan-1-one (E22)

The titled compound was prepared from **D_10_**(100 mg, 0.26 mmol) and 4-(trifluoromethyl)phenol (64.8 mg, 0.39 mmol), purified by column chromatography on silica gel with the elution fluid of Hexane/EA (50:1–20:1). Pale yellow solid, 111.4 mg, 92% yield. ^1^H NMR (400 MHz, Chloroform-*d*) *δ* 7.97–7.93 (m, 2H), 7.65 (d, *J* = 8.2 Hz, 2H), 7.56 (dd, *J* = 8.4, 5.8 Hz, 4H), 7.41–7.37 (m, 2H), 7.01 (d, *J* = 8.5 Hz, 2H), 5.30 (s, 2H).

General procedure for the synthesis of compound **C_8_**_,_ **C_11_**–**C_12_**. To a solution of aniline (1 equiv) and phenylethanone (1 equiv) in toluene, Pd_2_(dba)_3_ (0.2 equiv), X-Phos (0.4 equiv) and Cs_2_CO_3_ (1.5 equiv) was added. The mixture was stirred under nitrogen at 80 °C for 24 h. After cooling to room temperature and filtration with diatomite, water was added and the filtrate was extracted with ethyl acetate for three times and washed with saturated brine. The combined organic layer was dried over anhydrous Na_2_SO_4_ and purified by column chromatography on silica gel to provide the titled compounds.

#### 1-(4-((4-(Trifluoromethyl)thiazol-2-yl)amino)phenyl)ethan-1-one (C8)

The titled compound was prepared from 4-(trifluoromethyl)thiazol-2-amine (1 g, 5.95 mmol) and 1–(4-bromophenyl)ethan-1-one (1.18 g, 5.95 mmol), purified by column chromatography on silica gel with the elution fluid of Hexane/EA (100:1–50:1). Pale yellow solid, 999.6 mg, 59% yield. ^1^H NMR (400 MHz, Chloroform-*d*) *δ* 8.05–8.01 (m, 2H), 7.55 (s, 1H), 7.50–7.46 (m, 2H), 7.21 (s, 1H), 2.61 (s, 3H).

#### 1-(6-((4-(Trifluoromethyl)phenyl)amino)pyridin-3-yl)ethan-1-one (C11)

The titled compound was prepared from 4-(trifluoromethyl)aniline (1 g, 6.21 mmol) and 1–(6-bromopyridin-3-yl)ethan-1-one (1.24 g, 6.21 mmol), purified by column chromatography on silica gel with the elution fluid of Hexane/EA (100:1–50:1). Pale yellow solid, 1.48 g, 86% yield. ^1^H NMR (400 MHz, Chloroform-*d*) *δ* 8.88 (s, 1H), 8.15 (dd, *J* = 8.8, 2.2 Hz, 1H), 7.63 (s, 4H), 7.18 (s, 1H), 6.88 (d, *J* = 8.8 Hz, 1H), 2.59 (s, 3H).

#### 1-(2-(Trifluoromethyl)-4-((4-(trifluoromethyl)phenyl)amino)phenyl)ethan-1-one (C12)

The titled compound was prepared from 4-(trifluoromethyl)aniline (1 g, 6.21 mmol) and 1–(4-bromo-2-(trifluoromethyl)phenyl)ethan-1-one (1.66 g, 6.21 mmol), purified by column chromatography on silica gel with the elution fluid of Hexane/EA (200:1–100:1). White solid, 1.98 g, 92% yield. ^1^H NMR (400 MHz, DMSO-*d*_6_) *δ* 9.39 (s, 1H), 7.89 (d, *J* = 8.4 Hz, 1H), 7.66 (d, *J* = 8.4 Hz, 2H), 7.47 (d, *J* = 7.5 Hz, 2H), 7.33 (d, *J* = 8.4 Hz, 2H).

General procedure for the synthesis of compound **D_8_**_,_ **D_11_**–**D_12_**. To a solution of selected compound **C_10_–C_12_** (1 equiv) in a mixture of CHCl_3_ and EA (1:1), CuBr_2_ (1.1 equiv) was added. The mixture was stirred at 60 °C and monitored to react completely. After cooling to room temperature and filtration with diatomite, the filtrate was concentrated and purified by column chromatography on silica gel to provide the titled compounds.

#### 2-Bromo-1-(4-((4-(trifluoromethyl)thiazol-2-yl)amino)phenyl)ethan-1-one (D8)

The titled compound was prepared from **C_8_** (500 mg, 1.44 mmol) and purified by column chromatography on silica gel with the elution fluid of Hexane/EA (200:1–150:1). Yellow solid, 190.1 mg, 30% yield. ^1^H NMR (600 MHz, DMSO-*d*_6_) *δ* 11.11 (d, *J* = 2.1 Hz, 1H), 8.03 (d, *J* = 8.6 Hz, 2H), 7.81 (d, *J* = 4.6 Hz, 1H), 7.74 (dd, *J* = 8.7, 1.5 Hz, 2H), 4.84 (d, *J* = 1.6 Hz, 2H).

#### 2-Bromo-1-(6-((4-(trifluoromethyl)phenyl)amino)pyridin-3-yl)ethan-1-one (D11)

The titled compound was prepared from **C_11_** (500 mg, 1.78 mmol) and purified by column chromatography on silica gel with the elution fluid of Hexane/EA (100:1–50:1). Pale yellow solid, 228.1 mg, 36% yield. ^1^H NMR (600 MHz, Chloroform-*d*) *δ* 8.87 (d, *J* = 2.3 Hz, 1H), 8.14 (dd, *J* = 8.8, 2.3 Hz, 1H), 7.65–7.59 (m, 4H), 7.19 (s, 1H), 6.89 (d, *J* = 8.8 Hz, 1H), 4.61 (s, 2H).

#### 2-Bromo-1-(2-(trifluoromethyl)-4-((4-(trifluoromethyl)phenyl)amino)phenyl)ethan-1-one (D12)

The titled compound was prepared from **C_12_** (500 mg, 1.44 mmol) and purified by column chromatography on silica gel with the elution fluid of Hexane/EA (200:1–150:1). Pale yellow solid, 279.2 mg, 46% yield. ^1^H NMR (600 MHz, Chloroform-*d*) *δ* 7.62 (dd, *J* = 13.1, 8.4 Hz, 3H), 7.42 (d, *J* = 2.3 Hz, 1H), 7.31–7.28 (m, 1H), 7.25 (d, *J* = 8.3 Hz, 2H), 6.47 (s, 1H), 4.39 (s, 2H).

General procedure for the synthesis of compound **E_20_**_,_ **E_25_**–**E_26_**. To a solution of 4-(trifluoromethyl)phenol (1.5 equiv) in acetone, K_2_CO_3_ (2 equiv) was added. The mixture was stirred at room temperature for 30 min. With the following adding of selected compound **D_8_**_,_ **D_11_**–**D_12_** (1 equiv), it was stirred at reflux for overnight and monitored to react completely. After cooling to room temperature and filtration, the filtrate was concentrated and purified by column chromatography on silica gel to provide the titled compounds.

#### 2-(4-(Trifluoromethyl)phenoxy)-1-(4-((4-(trifluoromethyl)thiazol-2-yl)amino)phenyl)ethan-1-one (E20)

The titled compound was prepared from **D_8_**(100 mg, 0.27 mmol) and 4-(trifluoromethyl)phenol (66.6 mg, 0.41 mmol), purified by column chromatography on silica gel with the elution fluid of Hexane/EA (50:1–20:1). Pale yellow solid, 108.4 mg, 89% yield. ^1^H NMR (400 MHz, Chloroform-*d*) *δ* 8.07 (d, *J* = 8.7 Hz, 2H), 7.68 (s, 1H), 7.57 (dd, *J* = 8.8, 5.2 Hz, 4H), 7.24 (s, 1H), 7.02 (d, *J* = 8.5 Hz, 2H), 5.33 (s, 2H).

#### 2-(4-(Trifluoromethyl)phenoxy)-1-(6-((4-(trifluoromethyl)phenyl)amino)pyridin-3-yl)ethan-1-one (E25)

The titled compound was prepared from **D_11_**(100 mg, 0.28 mmol) and 4-(trifluoromethyl)phenol (67.7 mg, 0.42 mmol), purified by column chromatography on silica gel with the elution fluid of Hexane/EA (20:1–10:1). Pale yellow solid, 96.4 mg, 79% yield. ^1^H NMR (400 MHz, Chloroform-*d*) *δ* 8.95 (s, 1H), 8.20 (dd, *J* = 8.9, 2.0 Hz, 1H), 7.69–7.55 (m, 7H), 7.03 (d, *J* = 8.6 Hz, 2H), 6.93 (d, *J* = 8.9 Hz, 1H), 5.24 (s, 2H).

#### 1-(2-(Trifluoromethyl)-4-((4-(trifluoromethyl)phenyl)amino)phenyl)-2-(4-(trifluoromethyl)phenoxy)ethan-1-one (E26)

The titled compound was prepared from **D_12_**(100 mg, 0.23 mmol) and 4-(trifluoromethyl)phenol (57.1 mg, 0.35 mmol), purified by column chromatography on silica gel with the elution fluid of Hexane/EA (50:1–20:1). White solid, 96.8 mg, 81% yield. ^1^H NMR (400 MHz, Chloroform-*d*) *δ* 7.61 (dd, *J* = 8.4, 6.1 Hz, 3H), 7.55 (d, *J* = 8.6 Hz, 2H), 7.42 (d, *J* = 2.3 Hz, 1H), 7.29 (dd, *J* = 8.6, 2.3 Hz, 1H), 7.24 (d, *J* = 8.4 Hz, 2H), 6.97 (d, *J* = 8.6 Hz, 2H), 6.51 (s, 1H), 5.12 (s, 2H).

General procedure for the synthesis of compound **20** to **22, 29** and **30**. To a solution of selected compounds **E_20_**–**E_22_**, **E_25_**–**E_26_** (1 equiv) in MeOH, NaBH_4_ (3 equiv) was added in ice bath. After warming to room temperature, the mixture was stirred for 8 h and monitored to react completely. It was concentrated and purified by column chromatography on silica gel to provide the titled compounds.

#### 2-(4-(Trifluoromethyl)phenoxy)-1-(4-((4-(trifluoromethyl)thiazol-2-yl)amino)phenyl)ethan-1-ol (20)

The titled compound was prepared from **E_20_** (50 mg, 0.11 mmol) and purified by column chromatography on silica gel with the elution fluid of Hexane/EA (5:1–2:1). White solid, 45.3 mg, 90% yield, mp 168–169 °C. ^1^H NMR (400 MHz, DMSO-*d*_6_) *δ* 10.59 (s, 1H), 7.66–7.60 (m, 3H), 7.59–7.54 (m, 2H), 7.44 (d, *J* = 8.5 Hz, 2H), 7.13 (d, *J* = 8.6 Hz, 2H), 5.67 (d, *J* = 4.5 Hz, 1H), 4.91 (q, *J* = 5.5 Hz, 1H), 4.09 (d, *J* = 5.8 Hz, 2H). ^13^C NMR (101 MHz, DMSO-*d*_6_) *δ* 165.8, 161.9, 140.1, 136.3, 127.7, 127.4, 117.5, 115.5, 73.6, 70.8. ^19^F NMR (565 MHz, Chloroform-d) δ −61.57, −65.08. HRMS(ESI): calcd for C_19_H_14_F_6_N_2_O_2_S, [M - H]^−^ 447.0602; found, 447.0611. HPLC purity: 99.27%.

#### 2-(4-(Trifluoromethyl)phenoxy)-1-(4-(4-(trifluoromethyl)phenoxy)phenyl)ethan-1-ol (21)

The titled compound was prepared from **E_21_** (50 mg, 0.11 mmol) and purified by column chromatography on silica gel with the elution fluid of Hexane/EA (5:1–2:1). Pale yellow oil, 46 mg, 92% yield. ^1^H NMR (400 MHz, Chloroform-*d*) *δ* 7.64–7.46 (m, 6H), 7.14–6.98 (m, 6H), 5.19 (dd, *J* = 8.5, 3.3 Hz, 1H), 4.20–4.07 (m, 2H), 2.81 (s, 1H). ^13^C NMR (101 MHz, Chloroform-*d*) *δ* 160.7, 160.2, 155.8, 135.4, 128.1, 127.2, 127.2, 127.2, 127.1, 127.1, 127.1, 127.0, 125.6, 125.5, 125.3, 125.0, 123.8, 123.4, 122.9, 122.8, 120.0, 118.0, 114.6, 73.3, 72.0. ^19^F NMR (565 MHz, Chloroform-d) δ −61.56, −61.77. HRMS(ESI): calcd for C_22_H_16_F_6_O_3_, [M - H]^−^ 441.0925; found, 441.0926. HPLC purity: 95.68%.

#### 2-(4-(Trifluoromethyl)phenoxy)-1-(4-((4-(trifluoromethyl)phenyl)thio)phenyl)ethan-1-ol (22)

The titled compound was prepared from **E_22_** (50 mg, 0.11 mmol) and purified by column chromatography on silica gel with the elution fluid of Hexane/EA (5:1–2:1). Pale yellow solid, 45 mg, 90% yield, mp 79–80 °C. ^1^H NMR (600 MHz, Chloroform-*d*) *δ* 7.58 (d, *J* = 8.6 Hz, 2H), 7.52 (d, *J* = 9.1 Hz, 6H), 7.32 (d, *J* = 8.2 Hz, 2H), 7.01 (d, *J* = 8.5 Hz, 2H), 5.20 (dt, *J* = 8.6, 2.6 Hz, 1H), 4.18 (dd, *J* = 9.4, 3.3 Hz, 1H), 4.09 (t, *J* = 9.0 Hz, 1H), 2.82 (d, *J* = 6.6 Hz, 1H). ^13^C NMR (151 MHz, Chloroform-*d*) *δ* 160.7, 142.3, 139.8, 133.4, 132.9, 128.7, 128.4, 128.2, 127.6, 127.1, 127.1, 127.1, 127.0, 127.0, 125.9, 125.9, 125.9, 125.9, 125.2, 124.9, 123.8, 123.6, 123.4, 123.1, 114.6, 73.2, 72.0. ^19^F NMR (565 MHz, Chloroform-d) δ −61.59, −62.50. HRMS(ESI): calcd for C_22_H_16_F_6_O_2_S, [M - H]^−^ 457.0697; found, 457.0707. HPLC purity: 98.94%.

#### 2-(4-(Trifluoromethyl)phenoxy)-1-(6-((4-(trifluoromethyl)phenyl)amino)pyridin-3-yl)ethan-1-ol (29)

The titled compound was prepared from **E_25_** (50 mg, 0.11 mmol) and purified by column chromatography on silica gel with the elution fluid of Hexane/EA (2:1–1:1). Pale yellow solid, 27.8 mg, 55% yield, mp 46–48 °C. ^1^H NMR (600 MHz, Chloroform-*d*) *δ* 8.33 (s, 1H), 7.72 (dd, *J* = 8.6, 2.2 Hz, 1H), 7.61–7.55 (m, 4H), 7.52–7.48 (m, 2H), 6.98 (dd, *J* = 27.0, 8.5 Hz, 4H), 5.13 (dd, *J* = 8.0, 3.8 Hz, 1H), 4.16–4.10 (m, 2H). ^13^C NMR (151 MHz, Chloroform-*d*) *δ* 160.6, 154.8, 146.5, 143.5, 136.3, 127.2, 127.1, 127.1, 127.1, 127.0, 127.0, 126.6, 126.6, 126.5, 126.5, 125.2, 125.2, 124.0, 123.8, 123.6, 123.4, 123.4, 118.4, 114.6, 109.8, 72.9, 70.2. ^19^F NMR (565 MHz, Chloroform-d) δ −61.58, −61.77. HRMS(ESI): calcd for C_21_H_16_F_6_N_2_O_2_, [M + H]^+^ 443.1194; found, 473.1198. HPLC purity: 97.69%.

#### 1-(2-(Trifluoromethyl)-4-((4-(trifluoromethyl)phenyl)amino)phenyl)-2-(4-(trifluoromethyl)phenoxy)ethan-1-ol (30)

The titled compound was prepared from **E_26_** (50 mg, 0.09 mmol) and purified by column chromatography on silica gel with the elution fluid of Hexane/EA (5:1–2:1). White solid, 38.2 mg, 76% yield, mp 121–122 °C. ^1^H NMR (600 MHz, Chloroform-*d*) *δ* 7.83 (d, *J* = 8.4 Hz, 1H), 7.58 (t, *J* = 9.2 Hz, 4H), 7.43–7.38 (m, 2H), 7.14 (d, *J* = 8.3 Hz, 2H), 7.02 (d, *J* = 8.4 Hz, 2H), 6.13 (s, 1H), 5.53 (d, *J* = 8.7 Hz, 1H), 4.18 (dd, *J* = 9.6, 2.7 Hz, 1H), 4.03 (t, *J* = 9.2 Hz, 1H), 2.85 (s, 1H). ^13^C NMR (151 MHz, Chloroform-*d*) *δ* 160.7, 145.0, 141.9, 130.8, 130.0, 129.9, 129.0, 128.8, 128.6, 127.1, 127.1, 127.0, 127.0, 127.0, 127.0, 126.9, 125.2, 125.2, 124.8, 123.9, 123.7, 123.5, 123.5, 123.4, 123.4, 123.3, 123.0, 121.6, 116.7, 115.6, 115.6, 115.5, 115.5, 114.6, 72.9, 67.9. ^19^F NMR (565 MHz, Chloroform-d) δ −58.78, −61.58, −61.73. HRMS(ESI): calcd for C_23_H_16_F_9_NO_2_, [M - H]^−^ 508.0959; found, 508.0975. HPLC purity: 99.25%.

### Synthetic procedures for Scheme 3

#### Tert-butyl (4-(2-(4-(trifluoromethyl)phenoxy)ethyl)phenyl)carbamate (F1)

To a solution of *tert*-butyl (4–(2-hydroxyethyl)phenyl)carbamate (1 g, 4.21 mmol), 4-(trifluoromethyl)phenol (683.2 mg, 4.21 mmol) and triphenylphosphine (1.33 g, 5.06 mmol) in anhydrous THF, DEAD (880.7 mg, 5.06 mmol) was added in ice bath. The mixture was stirred in ice bath for 1 h and warmed to room temperature for overnight. When being monitored to react completely, it was concentrated and purified by column chromatography on silica gel with the elution fluid of Hexane/EA (20:1–10:1) to provide the titled compound. White solid, 1.11 g, 69% yield. ^1^H NMR (600 MHz, Chloroform-*d*) *δ* 7.52 (d, *J* = 8.4 Hz, 2H), 7.31 (d, *J* = 8.1 Hz, 2H), 7.20 (d, *J* = 8.0 Hz, 2H), 6.93 (d, *J* = 8.4 Hz, 2H), 4.16 (t, *J* = 7.0 Hz, 2H), 3.05 (t, *J* = 7.0 Hz, 2H), 1.52 (s, 9H).

#### 4-(2-(4-(Trifluoromethyl)phenoxy)ethyl)aniline (G1)

To a solution of **F_1_** (500 mg, 1.31 mmol) in DCM, TFA (1 ml, 13.11 mmol) was added. The mixture was stirred at reflux for overnight and monitored to react completely. After cooling to room temperature, 1 M NaOH aqueous solution was added in ice bath to regulate the pH value ranging from 8 to 10. The mixture was extracted with DCM for three times and washed with saturated brine. The combined organic layer was dried over anhydrous Na_2_SO_4_ and purified by column chromatography on silica gel with the elution fluid of Hexane/EA (5:1–2:1, with additional drops of aqueous ammonia) to provide the titled compound. Pale yellow oil, 352.9 mg, 96% yield. ^1^H NMR (400 MHz, Chloroform-*d*) *δ* 7.56 (d, *J* = 8.6 Hz, 2H), 7.14–7.07 (m, 2H), 6.97 (d, *J* = 8.6 Hz, 2H), 6.71–6.66 (m, 2H), 4.17 (t, *J* = 7.2 Hz, 2H), 3.66 (s, 2H), 3.04 (t, *J* = 7.2 Hz, 2H).

#### 4-(Trifluoromethyl)-N-(4-(2-(4-(trifluoromethyl)phenoxy)ethyl)phenyl)aniline (25)

To a solution of 1-iodo-4-(trifluoromethyl)benzene (48.3 mg, 0.18 mmol) and **G_1_** (50 mg, 0.18 mmol) in toluene, Pd(OAc)_2_ (7.9 mg, 0.04 mmol), X-Phos (16.9 mg, 0.04 mmol) and Cs_2_CO_3_ (86.9 mg, 0.27 mmol) was added. The mixture was stirred under nitrogen at 120 °C for 8 h. After cooling to room temperature and filtration with diatomite, water was added and the filtrate was extracted with ethyl acetate for three times and washed with saturated brine. The combined organic layer was dried over anhydrous Na_2_SO_4_ and purified by column chromatography on silica gel with the elution fluid of Hexane/EA (20:1–10:1) to provide the titled compound. Pale yellow oil, 67.4 mg, 89% yield. ^1^H NMR (400 MHz, Chloroform-*d*) *δ* 7.57 (d, *J* = 8.5 Hz, 2H), 7.50 (d, *J* = 8.4 Hz, 2H), 7.29 (d, *J* = 4.8 Hz, 2H), 7.15 (d, *J* = 8.1 Hz, 2H), 7.05 (d, *J* = 8.4 Hz, 2H), 7.00 (d, *J* = 8.5 Hz, 2H), 5.92 (s, 1H), 4.24 (t, *J* = 6.9 Hz, 2H), 3.13 (t, *J* = 6.9 Hz, 2H). ^13^C NMR (101 MHz, Chloroform-*d*) *δ* 161.3, 146.9, 139.7, 132.6, 130.1, 128.5, 127.0, 126.9, 126.9, 126.8, 126.8, 126.7, 126.7, 126.6, 126.0, 125.8, 123.4, 123.3, 123.1, 123.1, 122.8, 122.0, 121.7, 121.4, 120.5, 115.1, 114.5, 68.9, 35.0. ^19^F NMR (565 MHz, Chloroform-d) δ −61.41, −61.45. HRMS(ESI): calcd for C_22_H_17_F_6_NO, [M - H]^−^ 424.1136; found, 424.1144. HPLC purity: 100%.

#### Methyl 4-((4-(trifluoromethyl)phenyl)amino)benzoate (F2)

To a solution of 4-(trifluoromethyl)aniline (1 g, 6.21 mmol) and methyl 4-bromobenzoate (1.6 g, 7.45 mmol) in toluene, Pd(OAc)_2_ (83.6 mg, 0.37 mmol), BINAP (772.9 mg, 1.24 mmol) and K_2_CO_3_ (2.57 g, 18.62 mmol) was added. The mixture was stirred under nitrogen at 80 °C for 24 h. After cooling to room temperature and filtration with diatomite, water was added and the filtrate was extracted with ethyl acetate for three times and washed with saturated brine. The combined organic layer was dried over anhydrous Na_2_SO_4_ and purified by column chromatography on silica gel with the elution fluid of Hexane/EA (200:1–100:1) to provide the titled compound. White solid, 1.38 mg, 76% yield. ^1^H NMR (400 MHz, Chloroform-*d*) *δ* 8.01–7.97 (m, 2H), 7.57 (d, *J* = 8.4 Hz, 2H), 7.22 (d, *J* = 8.4 Hz, 2H), 7.15–7.10 (m, 2H), 6.31 (s, 1H), 3.92 (s, 3H).

#### 4-((4-(Trifluoromethyl)phenyl)amino)benzoic acid (G2)

To a solution of **F_2_** (500 mg, 1.69 mmol) in EtOH, NaOH (338.6 mg, 8.47 mmol) was added. The mixture was stirred at reflux for 4 h and monitored to react completely. After cooling to room temperature and being concentrated under vacuum, dilute hydrochloric acid (∼10%) was added in ice bath to regulate the pH value ranging from 3 to 5. The mixture was extracted with DCM for three times and washed with saturated brine. The combined organic layer was dried over anhydrous Na_2_SO_4_ and purified by column chromatography on silica gel with the elution fluid of Hexane/EA (10:1–5:1, with additional drops of acetic acid) to provide the titled compound. Pale yellow solid, 465.3 mg, 98% yield. ^1^H NMR (400 MHz, DMSO-*d*_6_) *δ* 12.48 (s, 1H), 9.13 (s, 1H), 7.85 (d, *J* = 8.6 Hz, 2H), 7.58 (d, *J* = 8.4 Hz, 2H), 7.29 (d, *J* = 8.4 Hz, 2H), 7.19 (d, *J* = 8.6 Hz, 2H).

#### N-(4-(Trifluoromethyl)benzyl)-4-((4-(trifluoromethyl)phenyl)amino)benzamide (27)

To a solution of **G_2_** (50 mg, 0.18 mmol), EDCI (82.8 mg, 0.53 mmol) and 4-DMAP (2.2 mg, 0.02 mmol) in DCM, (4-(trifluoromethyl)phenyl)methanamine (31.1 mg, 0.18 mmol) was added dropwise in ice bath. After warming to room temperature, the mixture was stirred for 24 h and monitored to react completely. It was concentrated and purified by column chromatography on silica gel with the elution fluid of Hexane/EA (50:1–20:1) to provide the titled compound. Pale yellow solid, 69.1 mg, 89% yield, mp 51–52 °C. ^1^H NMR (400 MHz, Chloroform-*d*) *δ* 7.80–7.74 (m, 2H), 7.56 (dd, *J* = 22.6, 8.2 Hz, 4H), 7.45 (d, *J* = 8.0 Hz, 2H), 7.19–7.10 (m, 4H), 6.70 (t, *J* = 5.9 Hz, 1H), 6.43 (s, 1H), 4.69 (d, *J* = 5.9 Hz, 2H). ^13^C NMR (101 MHz, Chloroform-*d*) *δ* 167.0, 145.2, 144.8, 142.5, 129.9, 129.6, 129.3, 128.8, 127.9, 126.8, 126.8, 126.8, 126.7, 126.6, 125.7, 125.7, 125.6, 125.6, 125.4, 123.6, 123.2, 123.0, 122.7, 117.4, 117.1, 43.5. ^19^F NMR (565 MHz, Chloroform-d) δ −61.75, −62.51. HRMS(ESI): calcd for C_22_H_16_F_6_N_2_O, [M + H]^+^ 439.1245; found, 439.1245. HPLC purity: 99.26%.

#### N-(4-(N-(4-(Trifluoromethyl)benzyl)sulfamoyl)phenyl)acetamide (F3)

To a solution of (4-(trifluoromethyl)phenyl)methanamine (749.6 mg, 4.28 mmol) and TEA (1.78 ml, 12.84 mmol) in anhydrous DCM, 4-acetamidobenzenesulfonyl chloride (1 g, 4.28 mmol) was added in ice bath. After warming to room temperature, the mixture was stirred for 12 h and monitored to react completely. It was concentrated and purified by column chromatography on silica gel with the elution fluid of Hexane/EA (10:1–5:1) to provide the titled compound. Pale yellow solid, 1.31 g, 83% yield. ^1^H NMR (400 MHz, Chloroform-*d*) *δ* 7.82 (d, *J* = 8.8 Hz, 2H), 7.67 (d, *J* = 8.5 Hz, 2H), 7.56 (d, *J* = 8.1 Hz, 2H), 7.36 (d, *J* = 8.2 Hz, 3H), 4.75 (t, *J* = 6.5 Hz, 1H), 4.24 (d, *J* = 6.4 Hz, 2H), 2.26 (s, 3H).

#### 4-Amino-N-(4-(trifluoromethyl)benzyl)benzenesulfonamide (G3)

To a solution of **F_3_** (500 mg, 1.34 mmol) in EtOH, dilute hydrochloric acid (∼10%, 170 ml) was added in ice bath. After warming to the room temperature, the mixture was stirred at 70 °C for 4 h and monitored to react completely. After cooling to room temperature and being concentrated under vacuum, 1 M NaOH aqueous solution was added in ice bath to regulate the pH value ranging from 8 to 10. The mixture was extracted with DCM for three times and washed with saturated brine. The combined organic layer was dried over anhydrous Na_2_SO_4_ and purified by column chromatography on silica gel with the elution fluid of Hexane/EA (5:1–2:1, with additional drops of aqueous ammonia) to provide the titled compound. Pale yellow solid, 421.8 mg, 95% yield. ^1^H NMR (400 MHz, Chloroform-*d*) *δ* 7.68–7.62 (m, 2H), 7.56 (d, *J* = 8.0 Hz, 2H), 7.37 (d, *J* = 7.9 Hz, 2H), 6.72–6.66 (m, 2H), 4.67 (d, *J* = 6.4 Hz, 1H), 4.20 (d, *J* = 6.5 Hz, 2H).

#### N-(4-(Trifluoromethyl)benzyl)-4-((4-(trifluoromethyl)phenyl)amino)benzenesulfonamide (28)

To a solution of **G_3_** (50 mg, 0.15 mmol) and 1-iodo-4-(trifluoromethyl)benzene (41.2 mg, 0.15 mmol) in toluene, Pd_2_(dba)_3_ (13.9 mg, 0.02 mmol), XantPhos (17.5 mg, 0.03 mmol) and NaO*t*Bu (29.1 mg, 0.3 mmol) was added. The mixture was stirred under nitrogen at 110 °C for 24 h. After cooling to room temperature and filtration with diatomite, water was added and the filtrate was extracted with ethyl acetate for three times and washed with saturated brine. The combined organic layer was dried over anhydrous Na_2_SO_4_ and purified by column chromatography on silica gel with the elution fluid of Hexane/EA (20:1–10:1) to provide the titled compound. White solid, 49.8 mg, 69% yield, mp 163–164 °C. ^1^H NMR (400 MHz, DMSO-*d*_6_) *δ* 9.21 (s, 1H), 8.11 (t, *J* = 6.4 Hz, 1H), 7.69–7.59 (m, 6H), 7.48 (d, *J* = 8.1 Hz, 2H), 7.29 (d, *J* = 8.4 Hz, 2H), 7.25–7.20 (m, 2H), 4.10–4.05 (m, 2H). ^13^C NMR (151 MHz, DMSO-*d*_6_) *δ* 146.2, 145.7, 142.9, 131.4, 128.9, 128.7, 128.3, 128.1, 127.3, 127.01, 127.0, 127.0, 125.9, 125.5, 125.5, 125.5, 125.4, 125.4, 124.1, 123.7, 121.4, 121.2, 117.6, 116.6, 45.9. ^19^F NMR (565 MHz, Chloroform-d) δ −61.91, −62.58. HRMS(ESI): calcd for C_21_H_16_F_6_N_2_O_2_S, [M - H]^−^ 473.0758; found, 473.0762. HPLC purity: 97.15%.

### Synthetic procedures for Scheme 4

#### *N-(4-(6-Nitro-2H-benzo[b][*1,4*]oxazin-3-yl)phenyl)-5-(trifluoromethyl)pyridin-2-amine (H1)*

To a solution of 2-amino-4-nitrophenol (64.4 mg, 0.42 mmol) in DCM, tetrabutylammonium hydrogen sulphate (18.9 mg, 0.06 mmol) and K_2_CO_3_ (76.9 mg, 0.56 mmol) was added. The mixture was stirred at room temperature for 2 h. With the following adding of **D_1_** (100 mg, 0.28 mmol), the solution was stirred at reflux for 6 h and monitored to react completely. After cooling to room temperature and filtration, it was concentrated and purified by column chromatography on silica gel with the elution fluid of Hexane/EA (20:1–10:1) to provide **H_1_**. Orange solid, 90.2 mg, 78% yield. ^1^H NMR (600 MHz, DMSO-*d*_6_) *δ* 10.09 (s, 1H), 8.59 (d, *J* = 3.0 Hz, 1H), 8.08 (t, *J* = 2.5 Hz, 1H), 8.06–8.00 (m, 3H), 7.96–7.91 (m, 3H), 7.14 (dd, *J* = 9.0, 3.2 Hz, 1H), 7.07 (t, *J* = 7.7 Hz, 1H), 5.41 (d, *J* = 3.2 Hz, 2H).

#### *4-(6-Nitro-2H-benzo[b][*1,4*]oxazin-3-yl)-N-(4-(trifluoromethyl)phenyl)aniline (H2)*

The titled compound was prepared from **D_2_**(100 mg, 0.28 mmol) and 2-amino-4-nitrophenol (64.6 mg, 0.42 mmol) in a similar manner as described for compound **H_1_**. The elution fluid of Hexane/EA (50:1–20:1) was used for chromatography. Orange solid, 89.8 mg, 78% yield. ^1^H NMR (600 MHz, DMSO-*d*_6_) *δ* 9.27 (s, 1H), 8.08–7.98 (m, 4H), 7.63 (d, *J* = 8.4 Hz, 2H), 7.32 (d, *J* = 8.4 Hz, 2H), 7.28 (d, *J* = 8.6 Hz, 2H), 7.14 (d, *J* = 8.8 Hz, 1H), 5.39 (s, 2H).

#### *N-(4-(6-Nitro-3,4-dihydro-2H-benzo[b][*1,4*]oxazin-3-yl)phenyl)-5-(trifluoromethyl)pyridin-2-amine (32)*

To a solution of **H_1_** (50 mg, 0.12 mmol) in MeOH, NaBH_4_ (9.13 mg, 0.24 mmol) was added in ice bath. After warming to room temperature, the mixture was stirred for overnight and monitored to react completely. It was concentrated and purified by column chromatography on silica gel with the elution fluid of Hexane/EA (10:1–5:1) to provide the titled compound. Yellow solid, 32.5 mg, 65% yield, mp 173–174 °C. ^1^H NMR (400 MHz, Chloroform-*d*) *δ* 8.49 (s, 1H), 7.72 (dd, *J* = 8.7, 2.4 Hz, 1H), 7.66 (dt, *J* = 8.5, 2.5 Hz, 1H), 7.60 (d, *J* = 2.6 Hz, 1H), 7.48 (d, *J* = 8.4 Hz, 2H), 7.41 (d, *J* = 8.3 Hz, 2H), 6.94–6.81 (m, 3H), 4.58–4.52 (m, 1H), 4.41 (dt, *J* = 10.7, 2.5 Hz, 1H), 4.33 (s, 1H), 4.08 (dd, *J* = 10.8, 8.5 Hz, 1H). ^13^C NMR (151 MHz, Chloroform-*d*) *δ* 157.7, 148.8, 146.1, 146.1, 142.1, 139.7, 134.9, 134.9, 133.9, 133.0, 129.9, 129.9, 128.2, 125.0, 121.3, 118.1, 117.9, 116.5, 115.2, 110.2, 107.9, 71.1, 53.1. ^19^F NMR (565 MHz, Chloroform-d) δ −61.45. HRMS(ESI): calcd for C_20_H_15_F_3_N_4_O_3_, [M + H]^+^ 417.1175; found, 417.1164. HPLC purity: 98.17%.

#### *4-(6-Nitro-3,4-dihydro-2H-benzo[b][*1,4*]oxazin-3-yl)-N-(4-(trifluoromethyl)phenyl)aniline (33)*

The titled compound was prepared from **H_2_**(50 mg, 0.12 mmol) in a similar manner as described for compound **32**. The elution fluid of Hexane/EA (20:1–10:1) was used for chromatography. Yellow solid, 31.8 mg, 63% yield, mp 152–154 °C. ^1^H NMR (400 MHz, Chloroform-*d*) *δ* 7.65 (dd, *J* = 8.8, 2.6 Hz, 1H), 7.59 (d, *J* = 2.6 Hz, 1H), 7.52 (d, *J* = 8.4 Hz, 2H), 7.35 (d, *J* = 8.3 Hz, 2H), 7.22–7.18 (m, 2H), 7.11 (d, *J* = 8.4 Hz, 2H), 6.92 (d, *J* = 8.8 Hz, 1H), 6.02 (s, 1H), 4.52 (dd, *J* = 8.6, 3.0 Hz, 1H), 4.40 (dt, *J* = 10.8, 2.4 Hz, 1H), 4.32 (s, 1H), 4.08 (dd, *J* = 10.8, 8.6 Hz, 1H). ^13^C NMR (101 MHz, Chloroform-*d*) *δ* 148.8, 146.0, 142.1, 141.9, 134.0, 131.6, 128.3, 126.8, 126.8, 119.6, 116.4, 116.0, 115.2, 110.2, 71.2, 53.1. ^19^F NMR (565 MHz, Chloroform-d) δ −61.57. HRMS(ESI): calcd for C_21_H_17_F_3_N_3_O_3_, [M + H]^+^ 416.1222; found, 416.1218. HPLC purity: 100%.

### Determination of MIC

The antimicrobial activity of the compounds was assessed using the broth microdilution method, following the guidelines established by the CLSI^16^. The test medium was cation-adjusted Mueller-Hinton broth (CA-MHB), or brain heart infusion (BHI) broth for *Streptococci*. Serial two-fold dilutions of the compounds were performed, ranging from 256 µg/mL to 0.25 µg/mL. The bacterial cell inoculum was adjusted to approximately 5 × 10^5^ CFU per mL. After 20 h of incubation at 37 °C, the results were recorded. The MIC was defined as the lowest antibiotic concentration without visible growth. Experiments were performed in duplicates.

### Binding inhibition assay

We employed previously established procedures for protein overproduction, purification, and inhibitor testing^14^^,^^22^. In brief, C-SmBiT-CH (250 nM in PBS) was added to 96-well plates and mixed with 20 µL of the compound at various concentrations. The mixture was then incubated for 10 min at 37 °C. Subsequently, C-LgBiT-NusG (250 nM in PBS) was added to each well and incubated for another 10 min at 37 °C. Following the final incubation step, an equal volume of Promega Nano-Glo^®^ Luciferase Assay Substrate (Promega) was added to the reaction mixture. The luminescence emitted was measured using a Synergy H1 plate reader (Agilent). The experiment was performed in triplicates with technical repeats for consistent results.

### Time-kill kinetics

We evaluated the compound’s dose- and time-dependent antimicrobial effects of on *S. aureus* strains under aerobic conditions, adapting from relevant CLSI guidelines^19^. *S. aureus* cells at the logarithmic growth phase were adjusted to approximately 1.5 × 10^6^ CFU/mL (colony-forming unit per mL) in CA-MHB broth supplemented with varying concentrations of the compound. As a control, bacteria were incubated in CA-MHB broth without the compound. The cultures were grown at 37 °C with agitation at 180 rpm. At specific time intervals, 20 µL samples were collected from each treatment group and subjected to a 10-fold serial dilution in sterile phosphate-buffered saline (PBS). From each dilution, 5 µL samples were spotted on Columbia blood agar plates. The plates were then incubated overnight at 37 °C, following which the number of viable bacteria in each sample was counted and expressed as CFU/mL. The entire experiment was performed in triplicate.

### Determination of MBC

The MBCs of the compounds and antibiotics were evaluated following CLSI guidelines^19^. After determining the MIC values, bacterial suspensions were prepared at concentrations ranging from 1× MIC to 16× MIC. Two 10 µL droplets from each concentration were plated onto Columbia blood agar. The plates were then incubated at 37 °C for 24 h. Following incubation, bacterial colonies were counted to estimate viable cell numbers, expressed as CFU/mL. The MBC was defined as the lowest concentration at which the CFU/mL count was reduced to below detectable levels compared to the untreated control. All experiments were performed in duplicate.

### Live-cell fluorescence imaging

Various *B. subtilis* strains were cultivated overnight on selective LB (Lennox formulation) agar plate at 37 °C. Single colony was picked and allowed to grow in LB medium (Lennox formulation) with selective antibiotic at 37 °C with shaking at 180 rpm overnight. The culture was then diluted to OD_600_ = 0.05 and allowed to grow until OD_600_ = 0.50 at 37 °C with agitation. Control antibiotics and compound **7** at 1× MIC were then added to the culture with xylose, followed by further incubation at 37 °C with shaking for 30 min. To visualise the nucleoid, DAPI was added to a final concentration of 1 µg/mL. For microscopic analysis, 2.5 µL of cell culture was placed onto a thin, freshly prepared 1.2% agarose gel and covered with a coverslip prior to imaging. Fluorescence images were captured using a Nikon Eclipse Ti2-E Live-cell Fluorescence Imaging System equipped with a 100×/1.45 oil objective, and the GFP signal was visualised using a FITC (fluorescein isothiocyanate) (525/50 emission) filter, whereas the DAPI signal was visualised using a DAPI (460/50 emission) filter. The digital images were analysed using ImageJ version 1.53k software^47^.

### Cell-based transcription assay

A previously developed luciferase-based bacterial reporter system^28^ was employed to assess the effects of compound **7** on bacterial transcription. Briefly, an overnight culture of the bacterial strain BS2019 was diluted to an OD_600_ = 0.05 and grown at 37 °C with shaking at 180 rpm until OD_600_ = 0.40. Cells were then treated with 1.0% (w/v) xylose and compound **7** at ½× MIC. After treatment, the cells were incubated at 37 °C with shaking at 180 rpm. At 2-min intervals, three 1 ml aliquots were collected for transcription analysis, with the aliquots immediately mixed with 400 µL of stop buffer (2% phenol, 60% ethanol, 10 mM EDTA) to arrest transcription. The aliquots were then centrifuged at 5,000 × *g* at 4 °C for 5 min to harvest cell pellets, which were stored at −80 °C until further use.

Transcription analysis was performed as previously^28^. Total RNA was extracted using Monarch^®^ Total RNA Miniprep Kit (New England Biolabs), followed by reverse transcription using the High-Capacity cDNA Reverse Transcription Kit (Applied Biosystems). Before qPCR analysis, cDNA samples were diluted to 2.5 ng/µL, as determined by using the Qubit^™^ ssDNA Assay Kit (Invitrogen) with the Qubit 4 Fluorometer. qPCR analysis was conducted using the forward primer (5′-TCCTTGAACAGGGAGGTGTGT-3′) and reverse primer (5′-CGATCTTCAGCCCATTTTCAC-3′), along with PowerUp^™^ SYBR^™^ Green Master Mix (Applied Biosystems). Gene expression levels were quantified using the $2^{-\Delta C_{T}}$ method, and statistical significance between the treated and the untreated groups was assessed using an unpaired *t*-test, with significance indicated by * (*p* < 0.05), ** (*p* < 0.01), and *** (*p* < 0.001).

## Supplementary Material

G055_Manuscript_Figures_JPEG.zip

Scheme_5_2_.jpg

## Data Availability

The authors confirm that the data supporting the findings of this study are available within the article and its supplemental materials.
