# Supplementary figures and images for: Development of triaryl antimicrobials by scaffold hopping from an aminopropanol hit targeting bacterial RNA polymerase-NusG interactions
Source: J Enzyme Inhib Med Chem. 2025 Aug 18;40(1):2543923. doi: 10.1080/14756366.2025.2543923 (PMC12364105; doi:10.1080/14756366.2025.2543923)

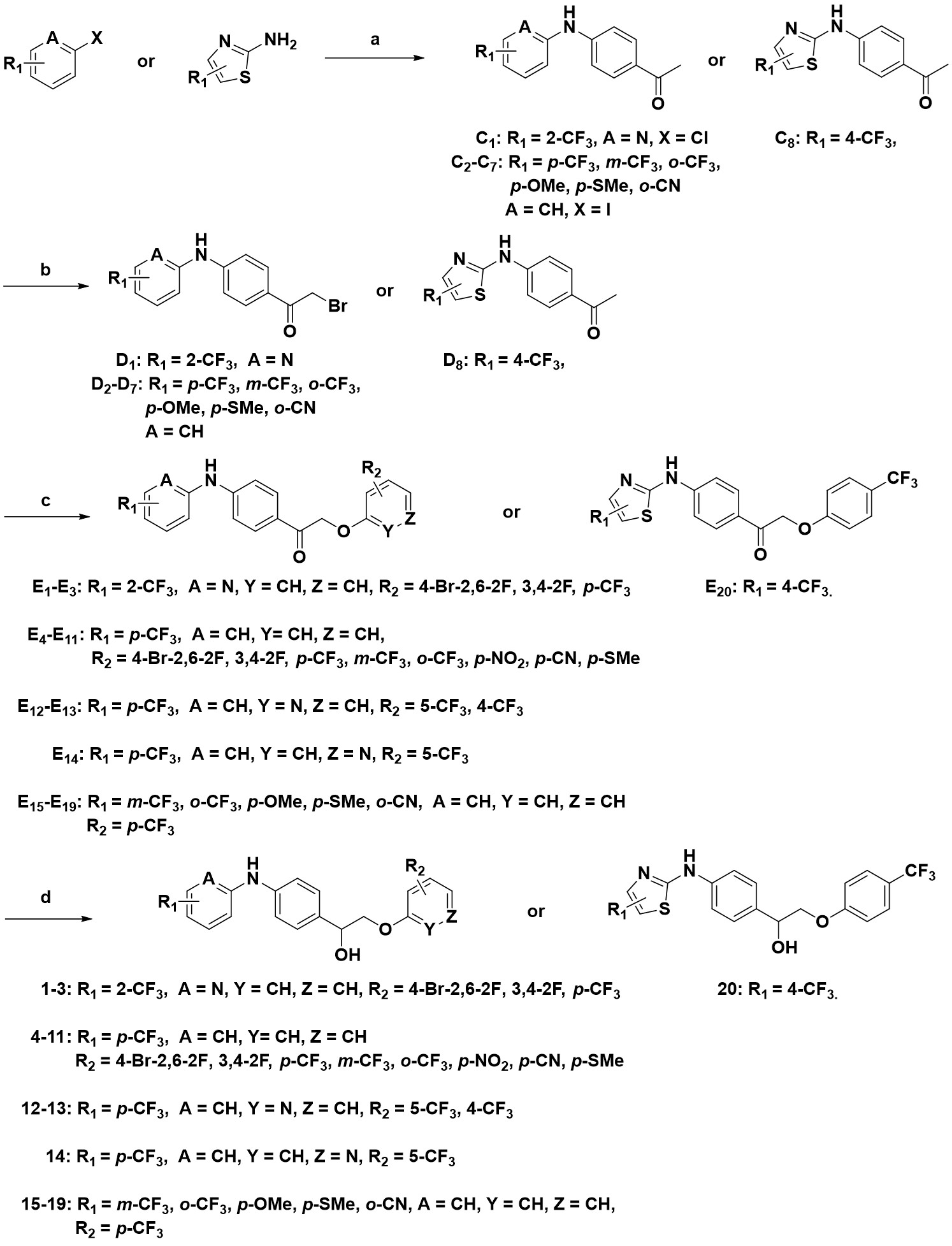

Supplement: G055_Manuscript_Figures_JPEG.zip [file IENZ_A_2543923_SM1358.zip › G055_Manuscript_Figures_JPEG_Chemistry/Scheme 1.jpg]

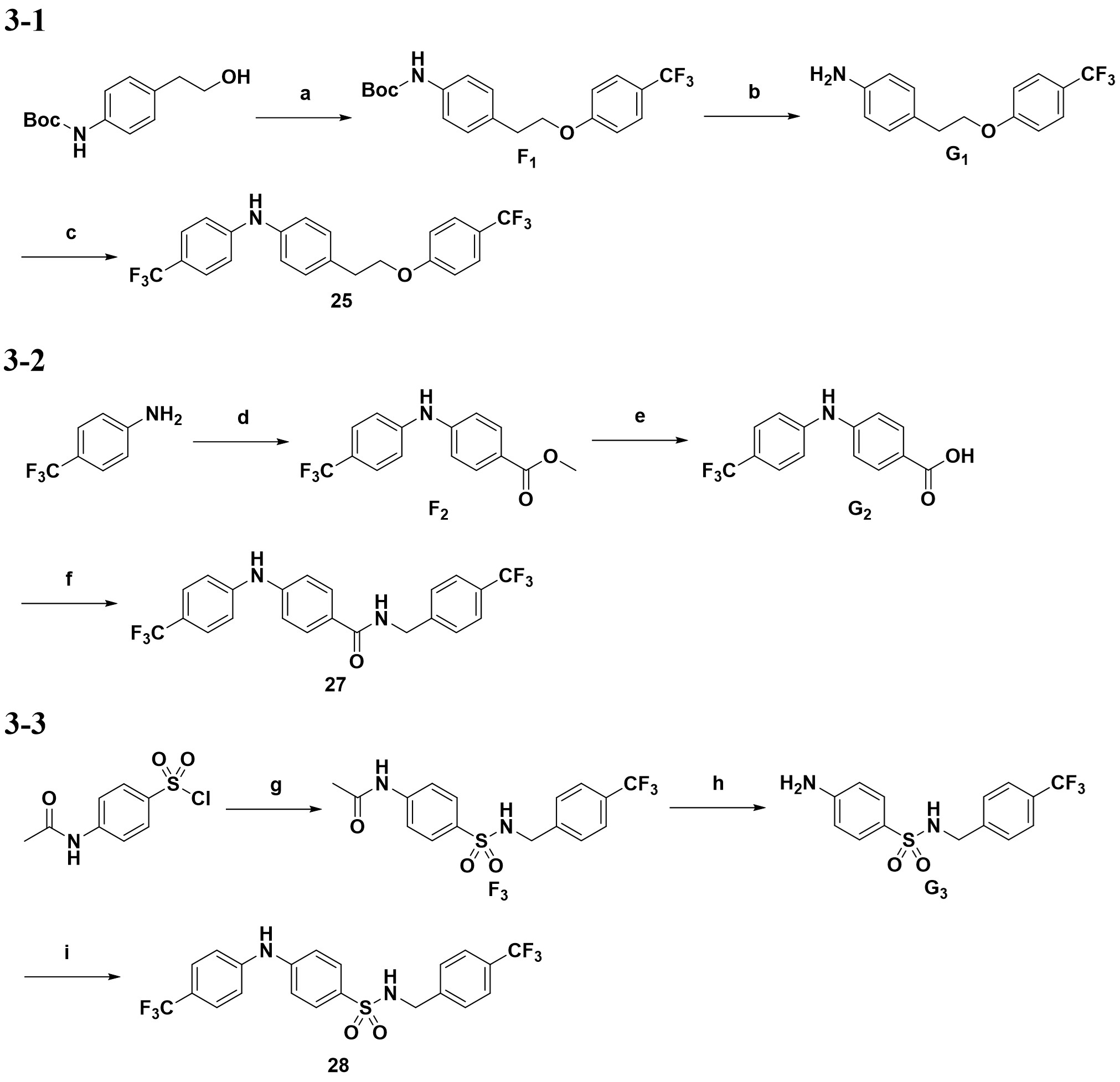

Supplement: G055_Manuscript_Figures_JPEG.zip [file IENZ_A_2543923_SM1358.zip › G055_Manuscript_Figures_JPEG_Chemistry/Scheme 3.jpg]

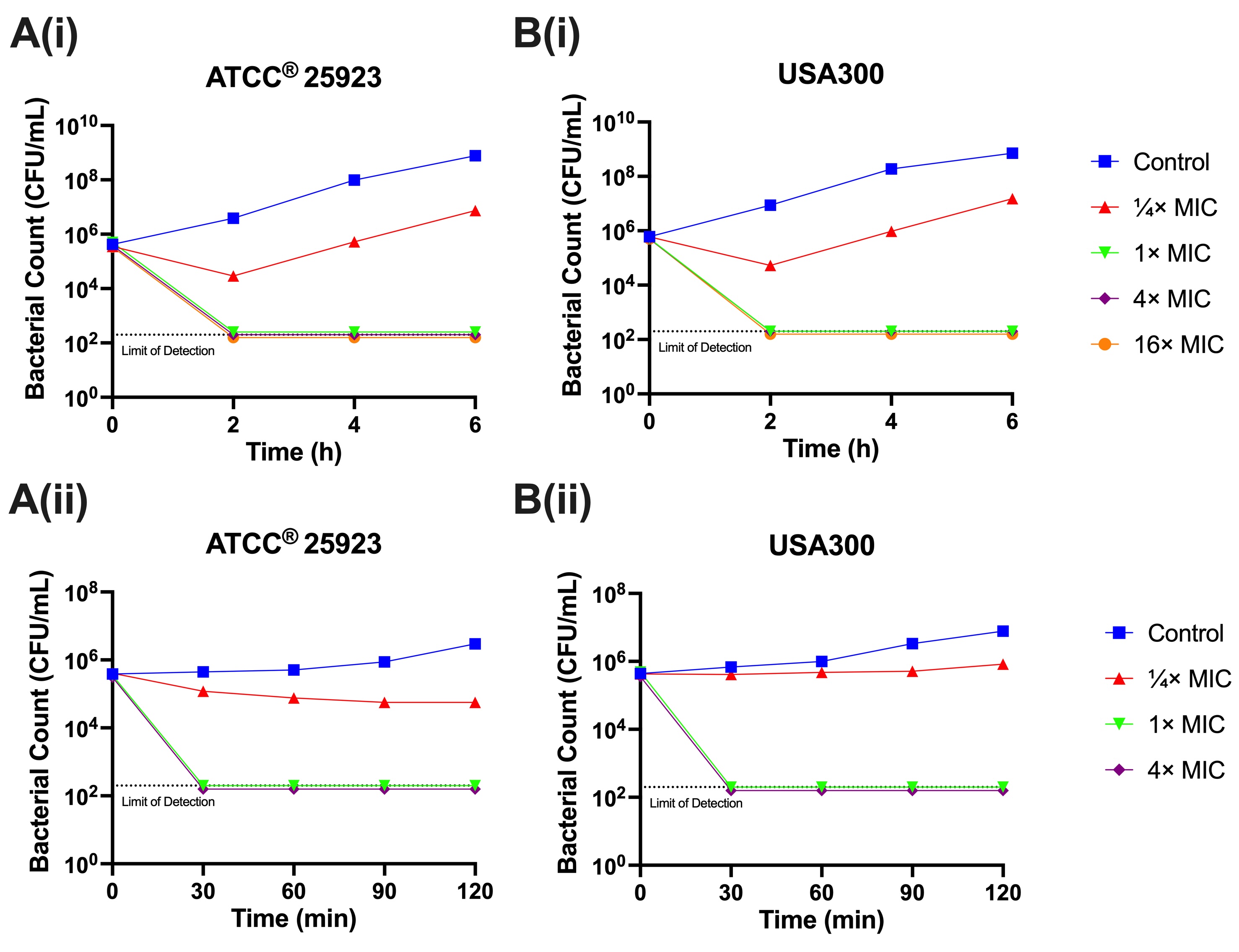

Supplement: G055_Manuscript_Figures_JPEG.zip [file IENZ_A_2543923_SM1358.zip › G055_Manuscript_Figures_JPEG_Chemistry/Figure 5.jpeg]

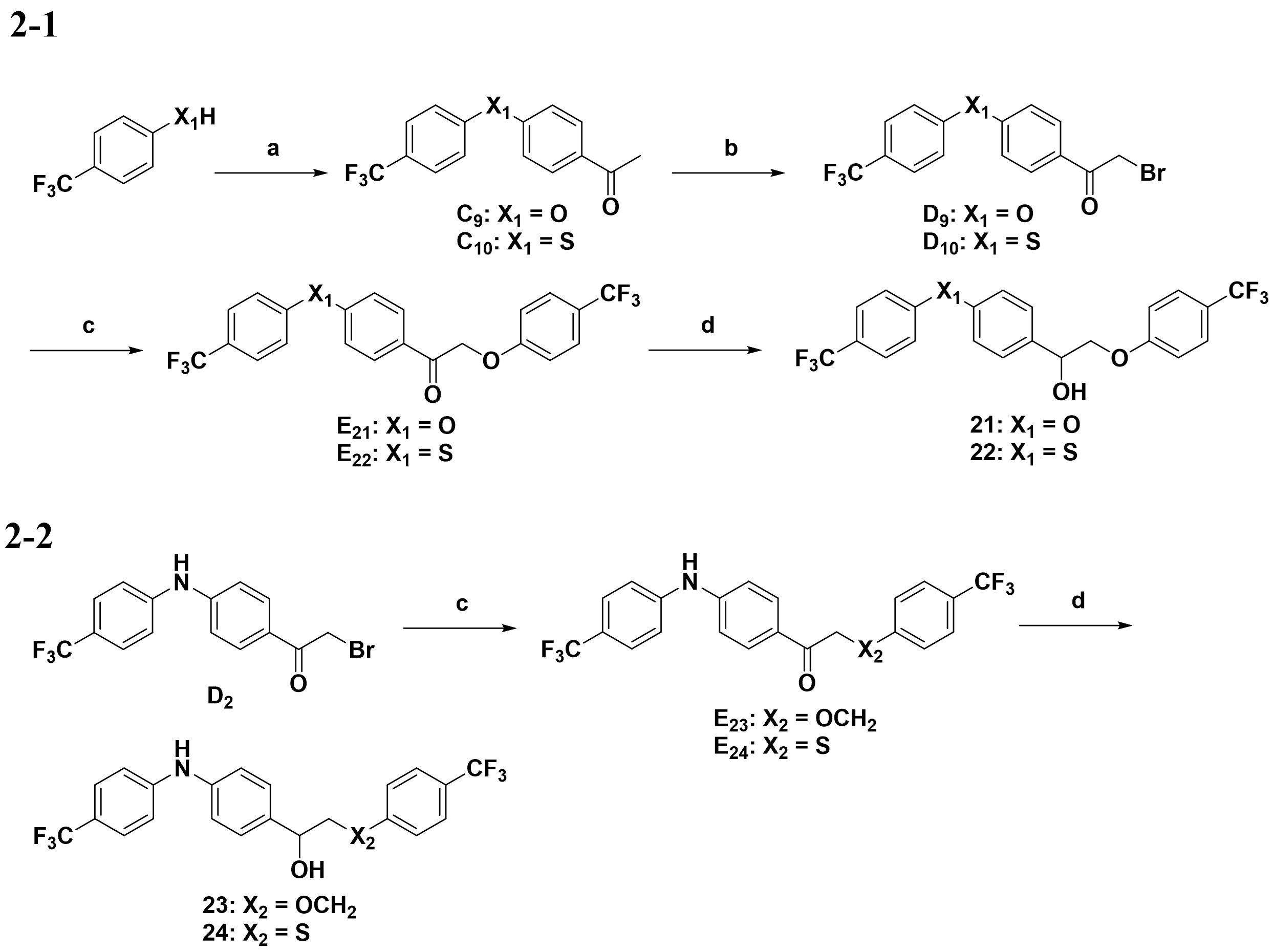

Supplement: G055_Manuscript_Figures_JPEG.zip [file IENZ_A_2543923_SM1358.zip › G055_Manuscript_Figures_JPEG_Chemistry/Scheme 2.jpg]

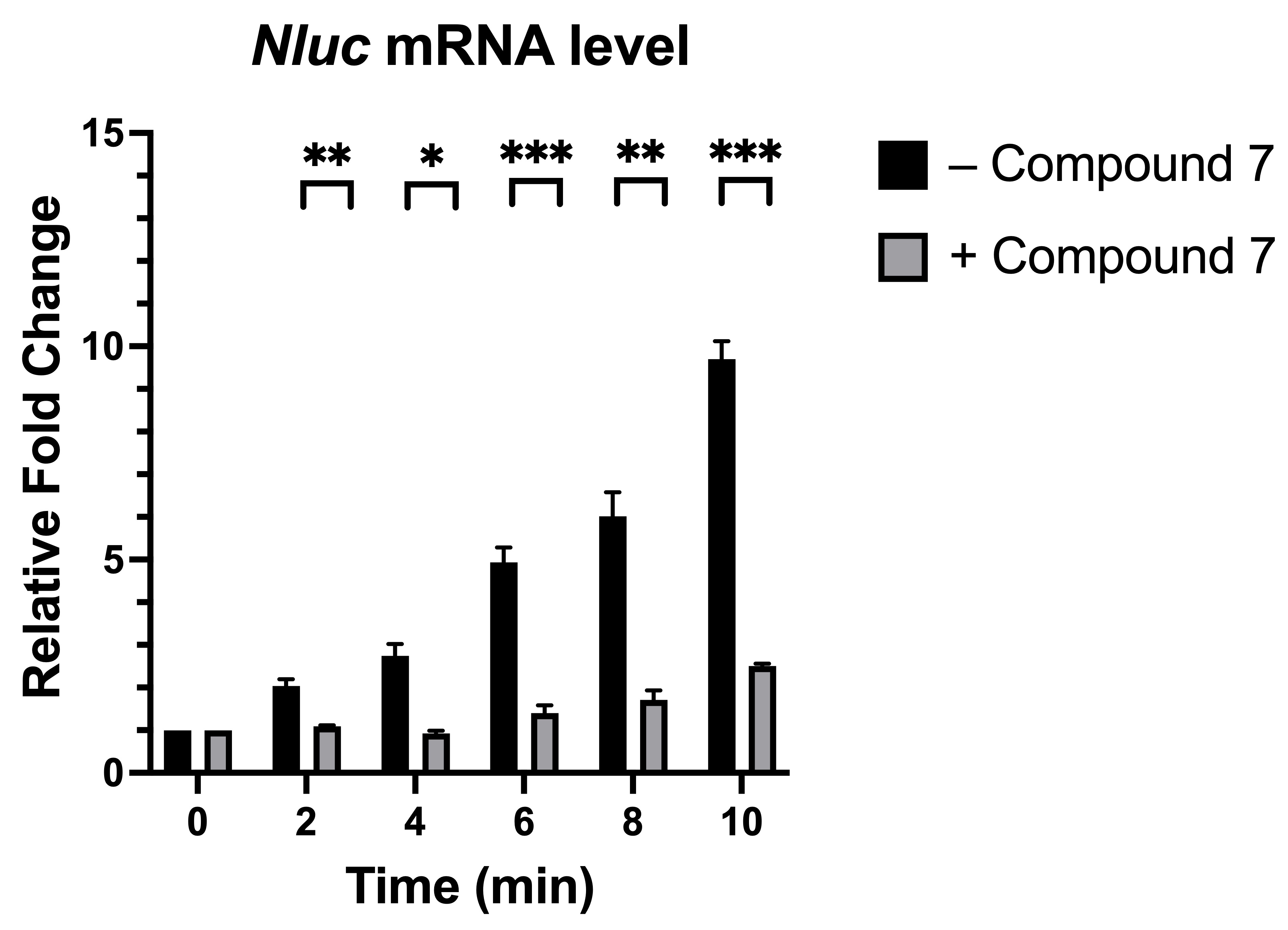

Supplement: G055_Manuscript_Figures_JPEG.zip [file IENZ_A_2543923_SM1358.zip › G055_Manuscript_Figures_JPEG_Chemistry/Figure 8.jpeg]

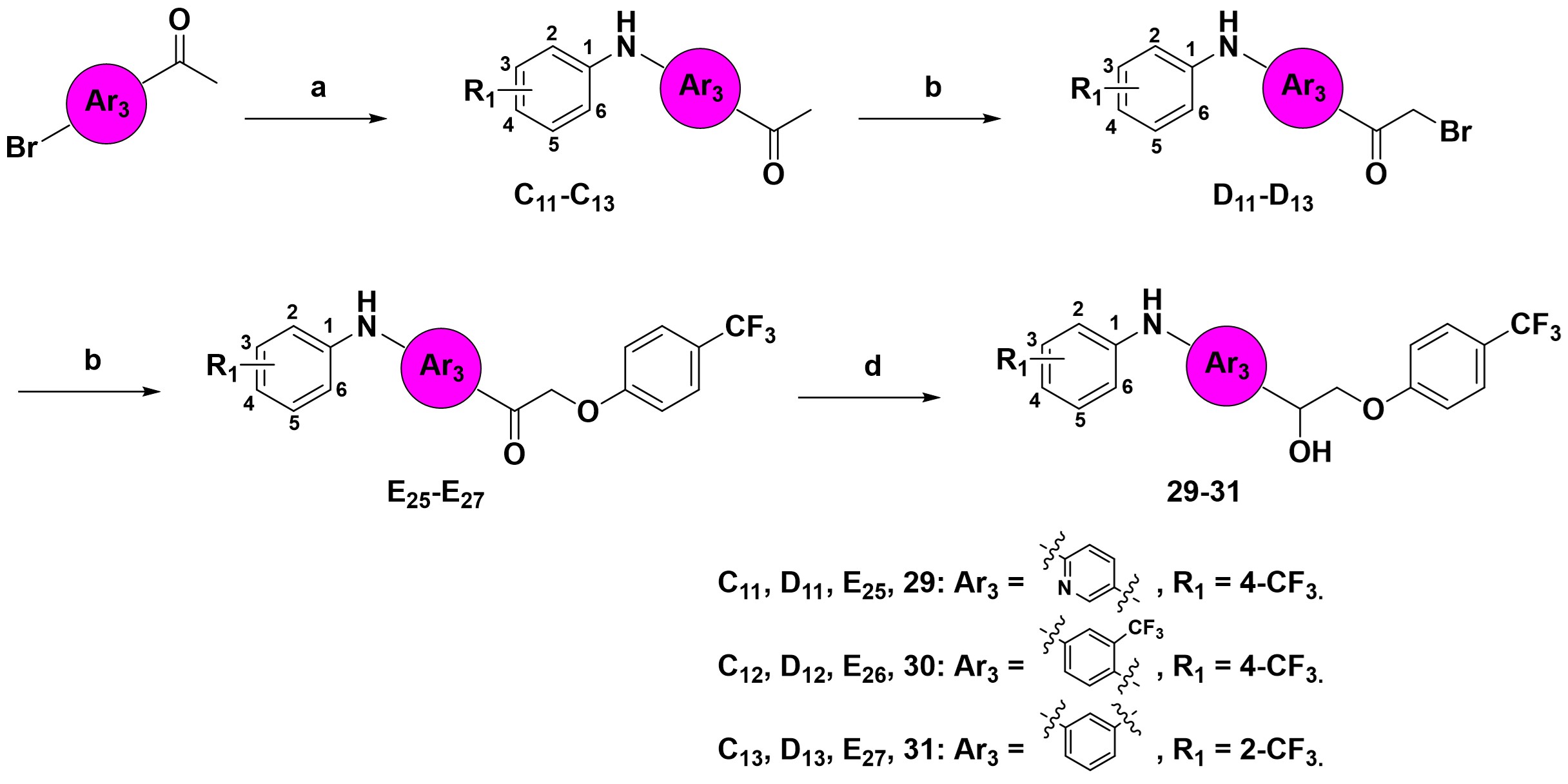

Supplement: G055_Manuscript_Figures_JPEG.zip [file IENZ_A_2543923_SM1358.zip › G055_Manuscript_Figures_JPEG_Chemistry/Scheme 4.jpg]

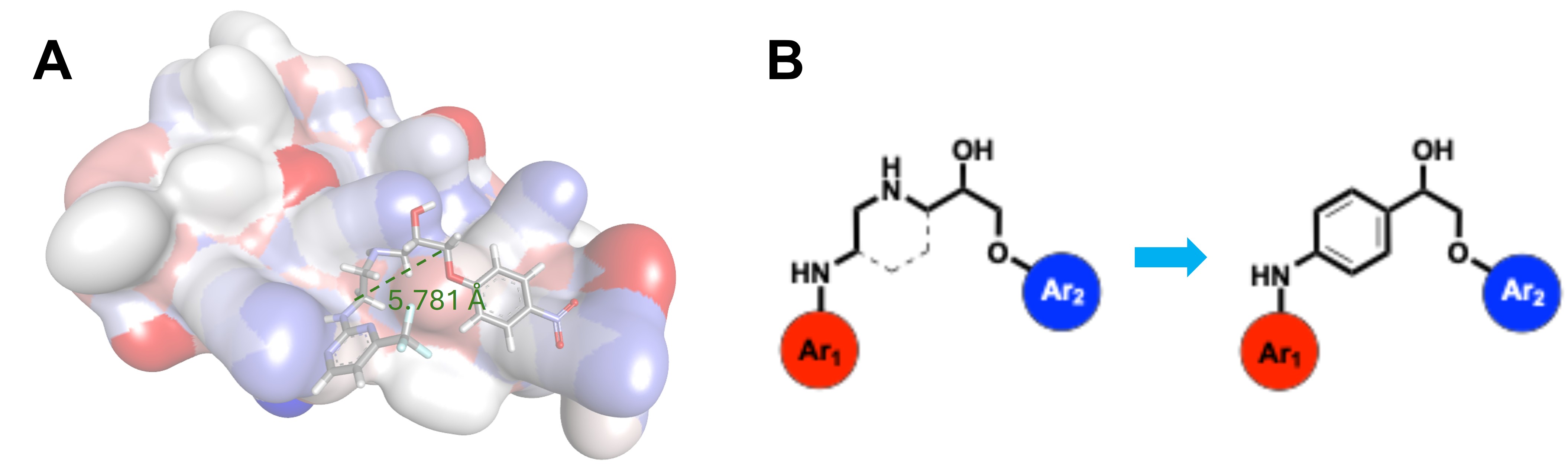

Supplement: G055_Manuscript_Figures_JPEG.zip [file IENZ_A_2543923_SM1358.zip › G055_Manuscript_Figures_JPEG_Chemistry/Figure 3.jpg]

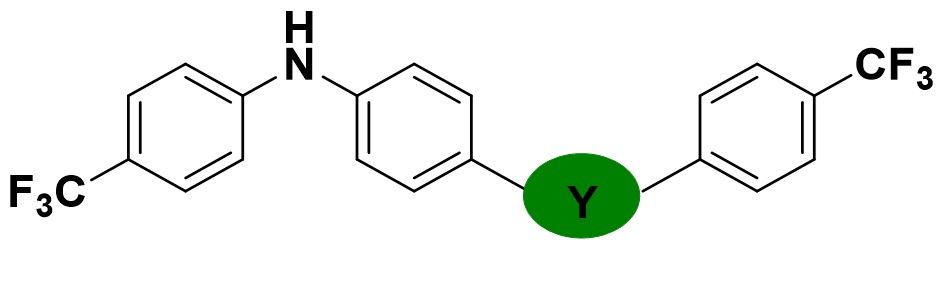

Supplement: G055_Manuscript_Figures_JPEG.zip [file IENZ_A_2543923_SM1358.zip › G055_Manuscript_Figures_JPEG_Chemistry/Table 3 Structure.jpg]

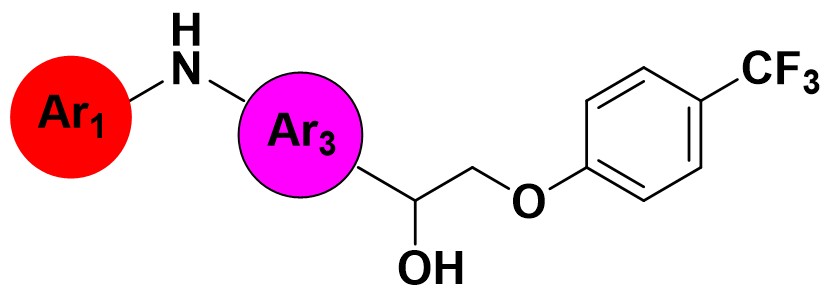

Supplement: G055_Manuscript_Figures_JPEG.zip [file IENZ_A_2543923_SM1358.zip › G055_Manuscript_Figures_JPEG_Chemistry/Table 4 Structure.jpg]

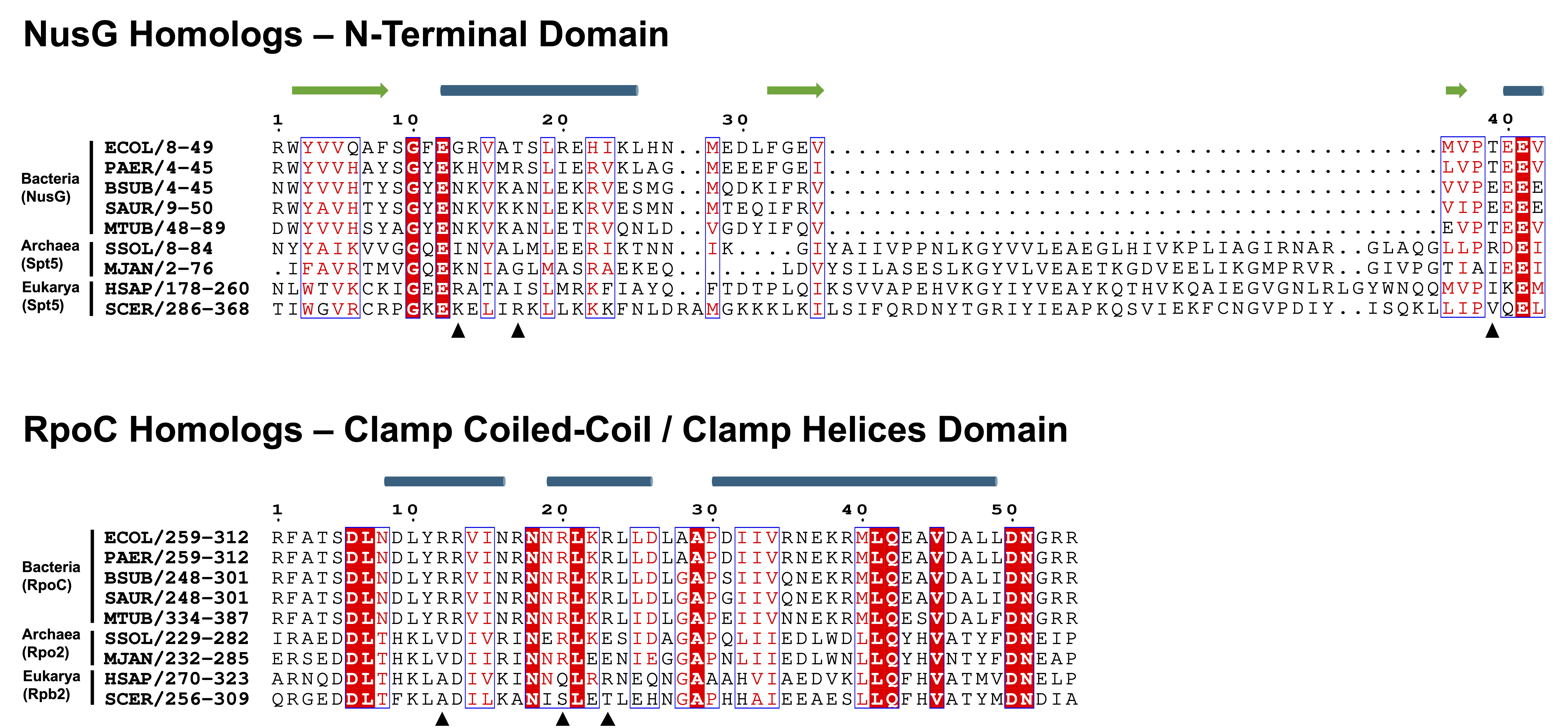

Supplement: G055_Manuscript_Figures_JPEG.zip [file IENZ_A_2543923_SM1358.zip › G055_Manuscript_Figures_JPEG_Chemistry/Figure 2.jpeg]

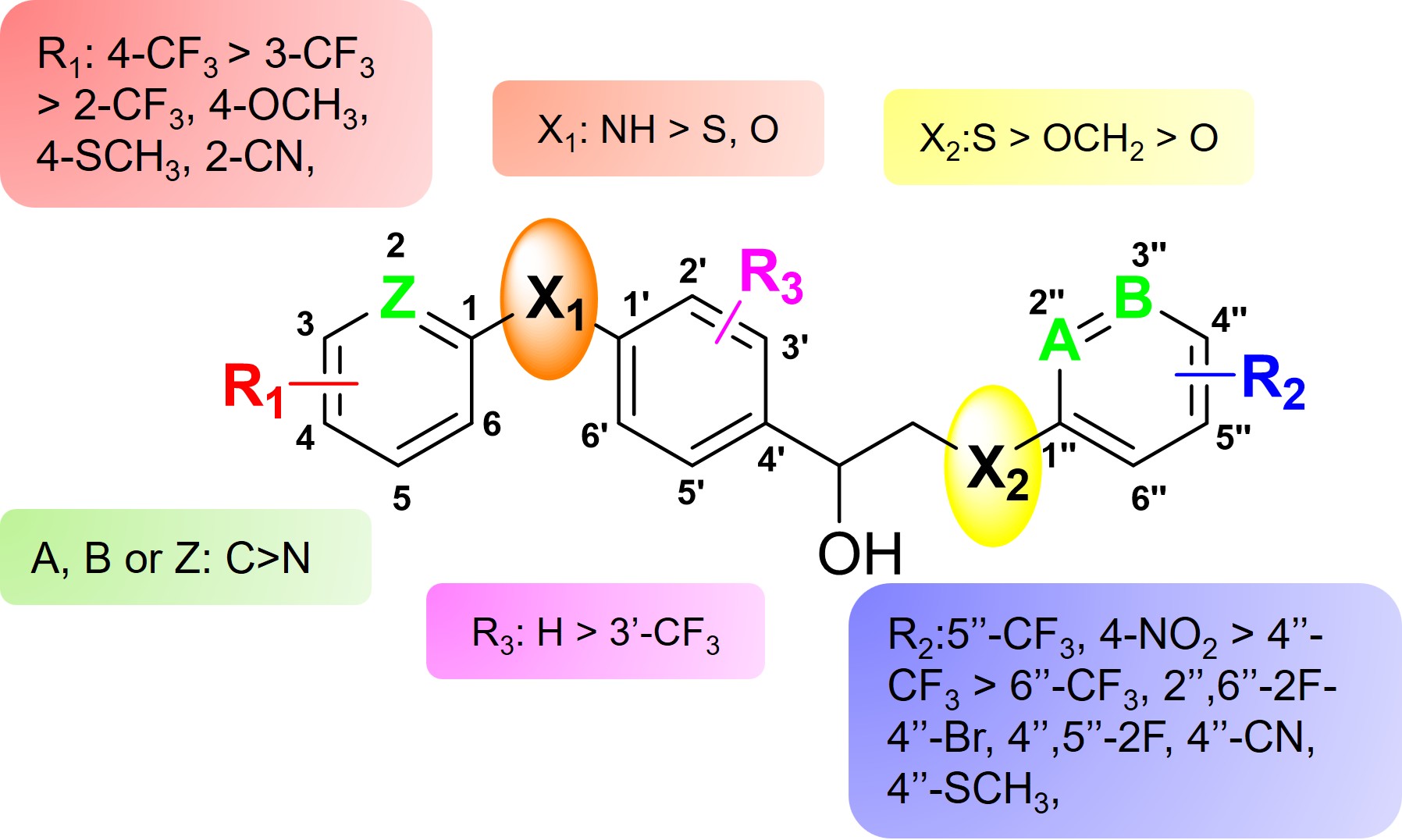

Supplement: G055_Manuscript_Figures_JPEG.zip [file IENZ_A_2543923_SM1358.zip › G055_Manuscript_Figures_JPEG_Chemistry/Figure 4.jpg]

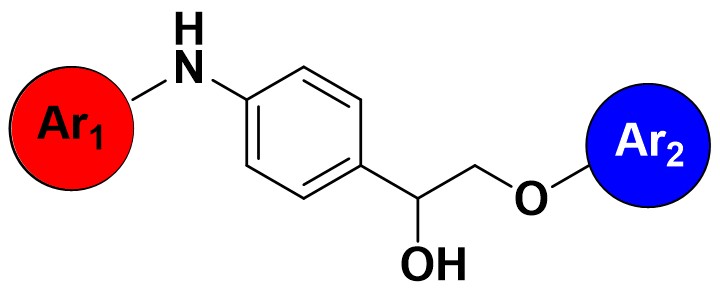

Supplement: G055_Manuscript_Figures_JPEG.zip [file IENZ_A_2543923_SM1358.zip › G055_Manuscript_Figures_JPEG_Chemistry/Table 1 Structure.jpg]

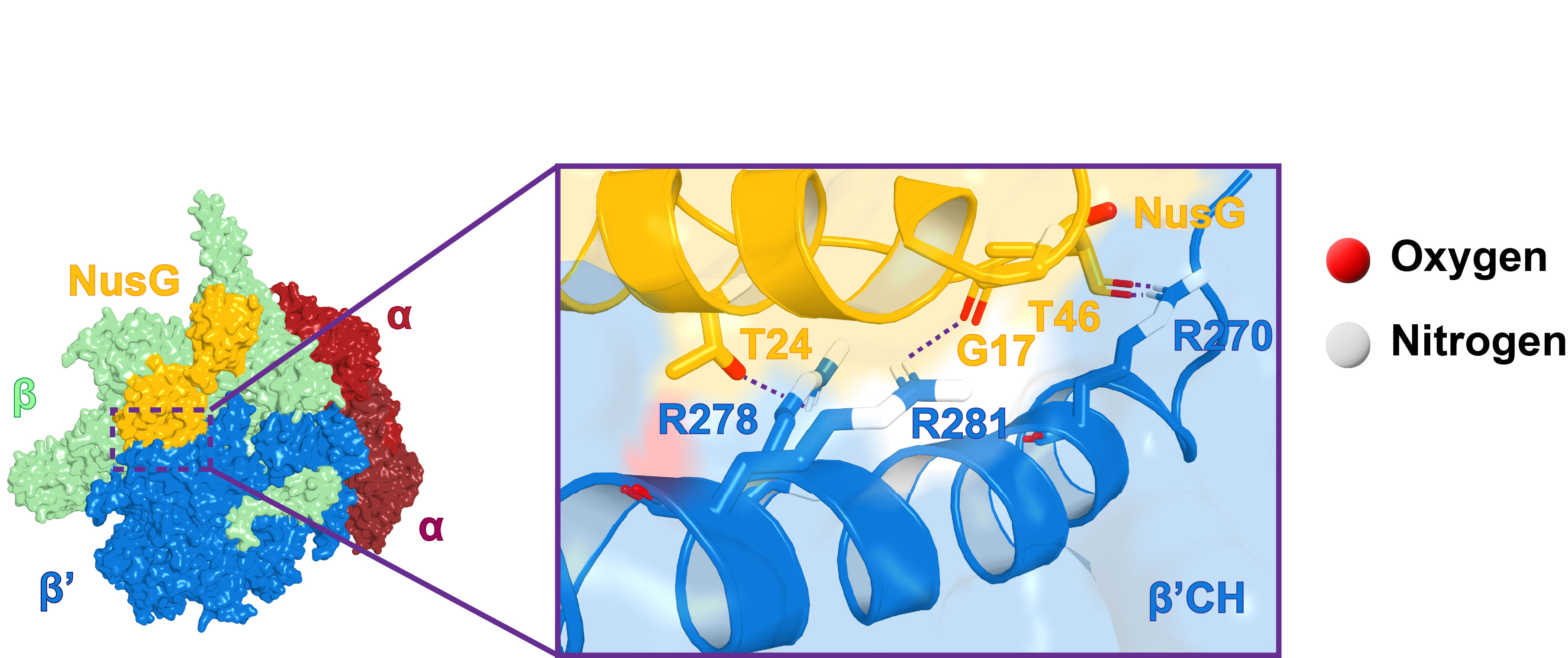

Supplement: G055_Manuscript_Figures_JPEG.zip [file IENZ_A_2543923_SM1358.zip › G055_Manuscript_Figures_JPEG_Chemistry/Figure 1.jpeg]

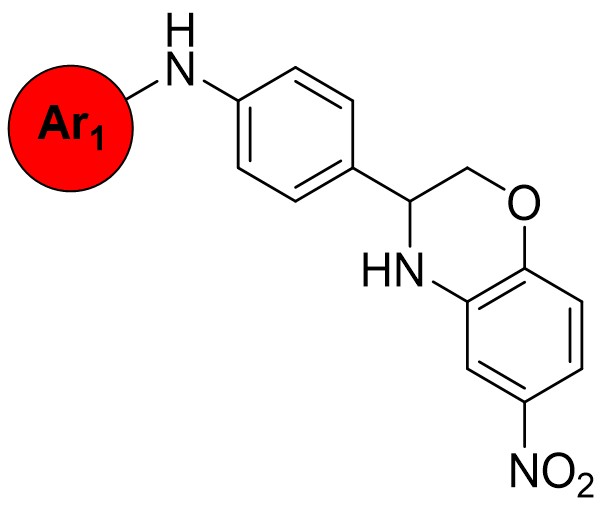

Supplement: G055_Manuscript_Figures_JPEG.zip [file IENZ_A_2543923_SM1358.zip › G055_Manuscript_Figures_JPEG_Chemistry/Table 5 Structure.jpg]

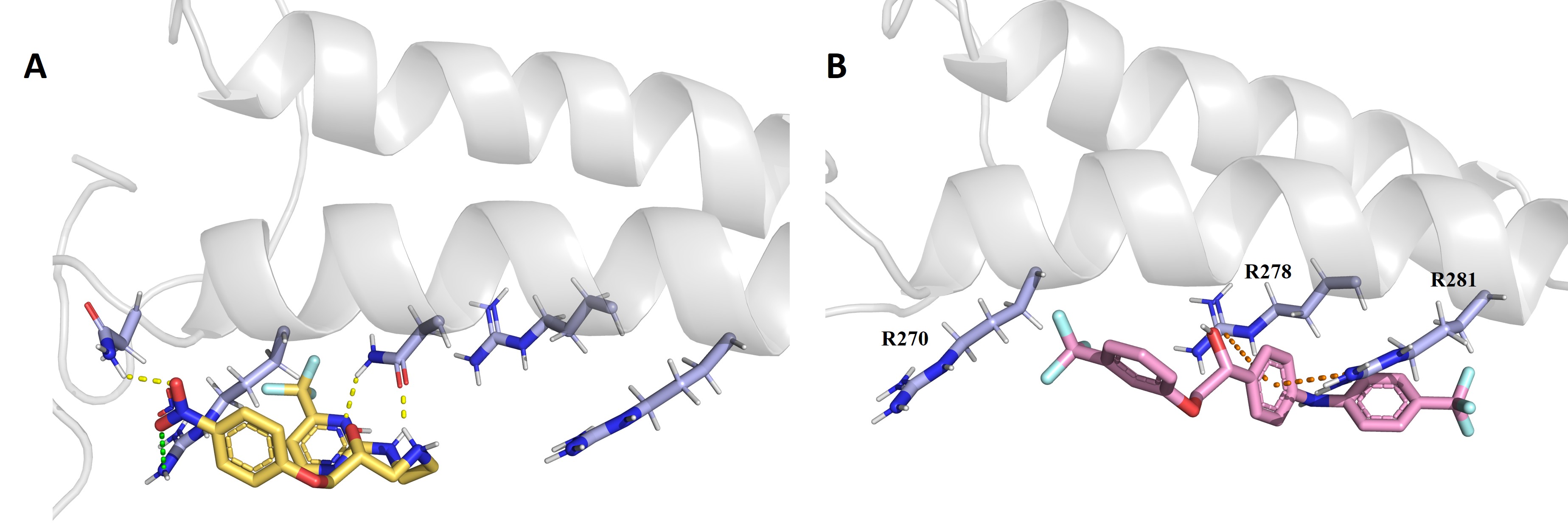

Supplement: G055_Manuscript_Figures_JPEG.zip [file IENZ_A_2543923_SM1358.zip › G055_Manuscript_Figures_JPEG_Chemistry/Figure 9.jpg]

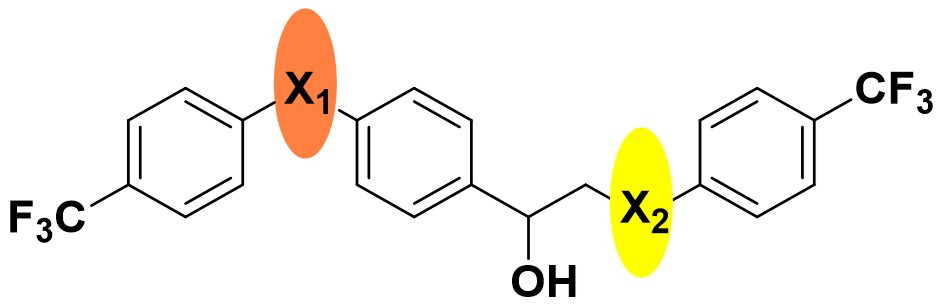

Supplement: G055_Manuscript_Figures_JPEG.zip [file IENZ_A_2543923_SM1358.zip › G055_Manuscript_Figures_JPEG_Chemistry/Table 2 Structure.jpg]

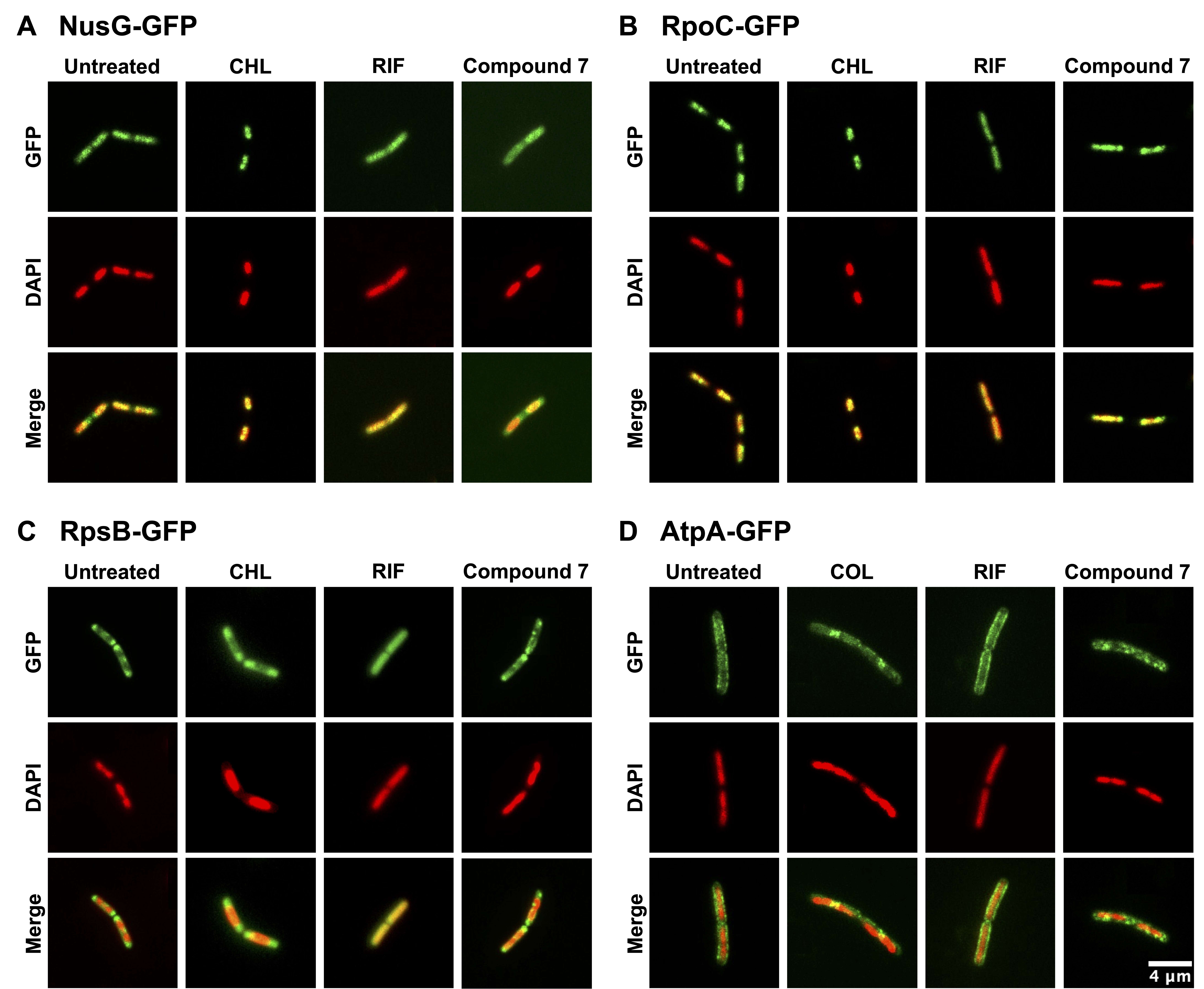

Supplement: G055_Manuscript_Figures_JPEG.zip [file IENZ_A_2543923_SM1358.zip › G055_Manuscript_Figures_JPEG_Chemistry/Figure 7.jpeg]

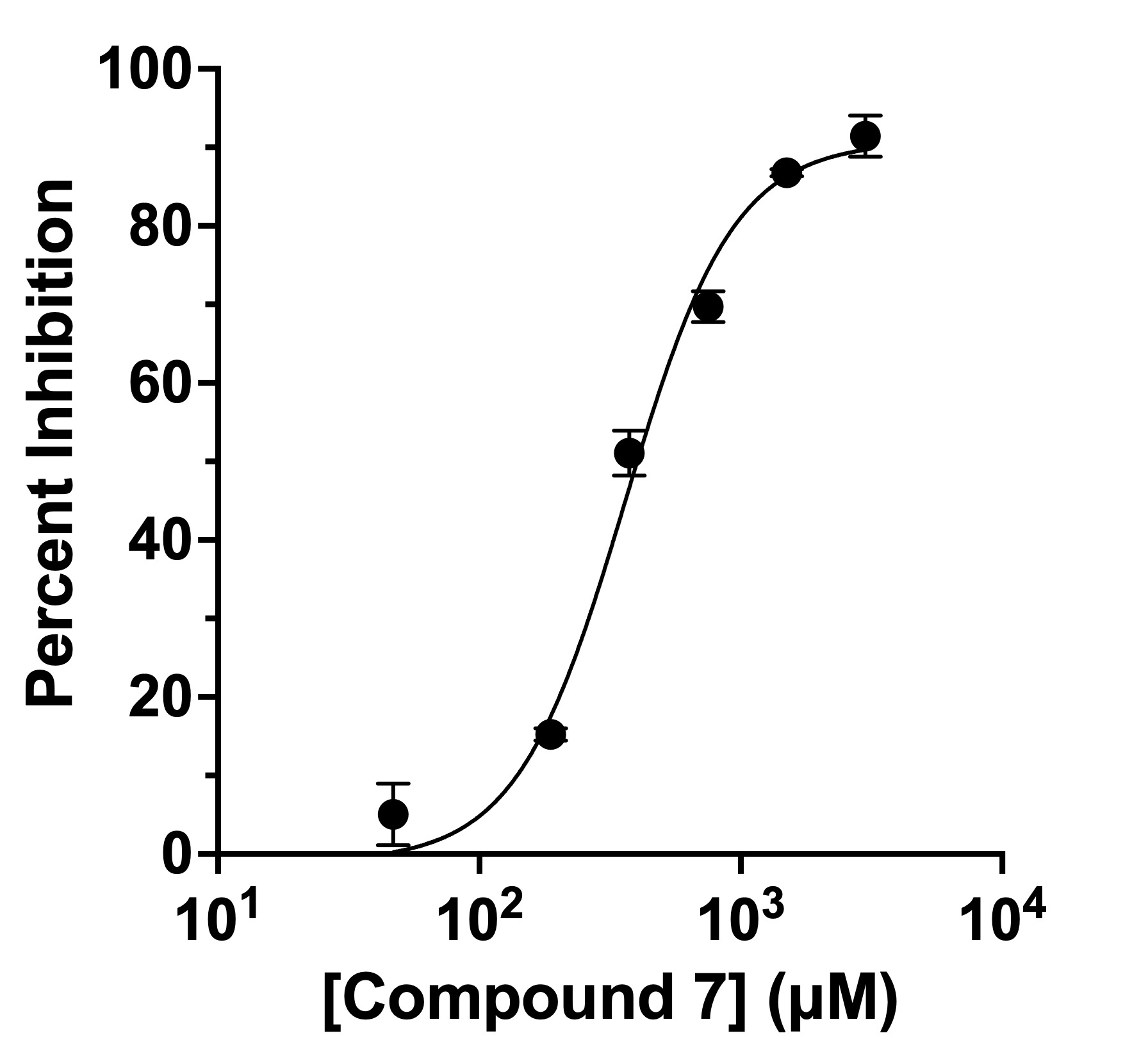

Supplement: G055_Manuscript_Figures_JPEG.zip [file IENZ_A_2543923_SM1358.zip › G055_Manuscript_Figures_JPEG_Chemistry/Figure 6.jpeg]

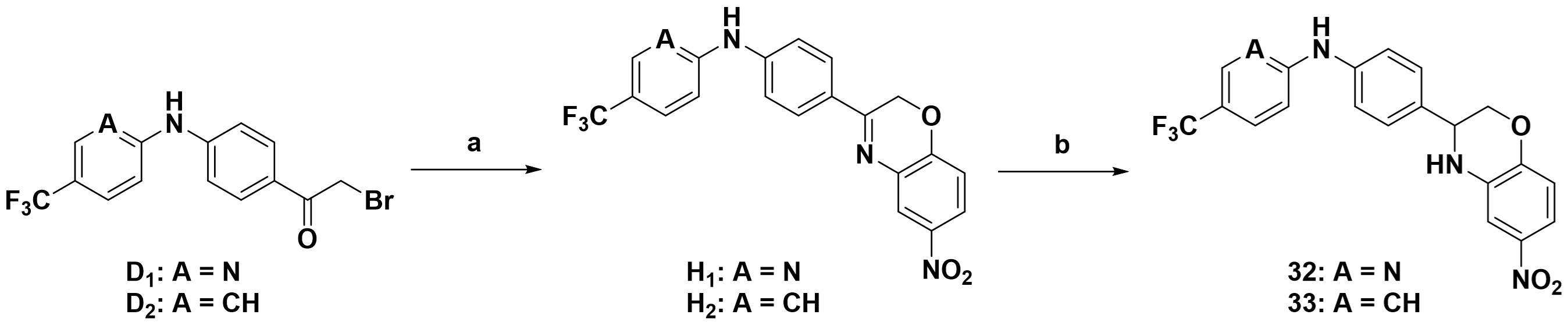

Supplement: Scheme_5_2_.jpg [file IENZ_A_2543923_SM1357.jpg]
